# LncRNA-SLC16A1-AS1 induces metabolic reprogramming during Bladder Cancer progression as target and co-activator of E2F1

**DOI:** 10.7150/thno.44176

**Published:** 2020-07-29

**Authors:** Stella Logotheti, Stephan Marquardt, Shailendra K. Gupta, Christin Richter, Berdien A.H. Edelhäuser, David Engelmann, Julia Brenmoehl, Christoph Söhnchen, Nico Murr, Michael Alpers, Krishna P. Singh, Olaf Wolkenhauer, Dirk Heckl, Alf Spitschak, Brigitte M. Pützer

**Affiliations:** 1 Institute of Experimental Gene Therapy and Cancer Research, Rostock University Medical Center, 18057 Rostock, Germany.; 2 Department Life, Light & Matter, University of Rostock, 18059 Rostock, Germany.; 3 Department of Systems Biology and Bioinformatics, University of Rostock, 18057 Rostock, Germany.; 4 Leibniz Institute for Farm Animal Biology (FBN), Institute of Genome Biology, Signal Transduction Unit, 18196 Dummerstorf, Germany.; 5 Pediatric Hematology and Oncology, Hannover Medical School, 30625 Hannover, Germany.

**Keywords:** bladder cancer, E2F1, metabolic reprogramming, RNA-protein complex, SLC16A1-AS1

## Abstract

Long non-coding RNAs (lncRNAs) have emerged as integral components of E2F1-regulated gene regulatory networks (GRNs), but their implication in advanced or treatment-refractory malignancy is unknown.

**Methods:** We combined high-throughput transcriptomic approaches with bioinformatics and structure modeling to search for lncRNAs that participate in E2F1-activated prometastatic GRNs and their phenotypic targets in the highly-relevant case of E2F1-driven aggressive bladder cancer (BC). RNA immunoprecipitation was performed to verify RNA-protein interactions. Functional analyses including qRT-PCR, immunoblotting, luciferase assays and measurement of extracellular fluxes were conducted to validate expression and target gene regulation.

**Results:** We identified E2F1-responsive lncRNA-SLC16A1-AS1 and its associated neighboring protein-coding gene, SLC16A1/MCT1, which both promote cancer invasiveness. Mechanistically, upon E2F1-mediated co-transactivation of the gene pair, SLC16A1-AS1 associates with E2F1 in a structure-dependent manner and forms an RNA-protein complex that enhances SLC16A1/MCT1 expression through binding to a composite SLC16A1-AS1:E2F1-responsive promoter element. Moreover, SLC16A1-AS1 increases aerobic glycolysis and mitochondrial respiration and fuels ATP production by fatty acid β-oxidation. These metabolic changes are accompanied by alterations in the expression of the SLC16A1-AS1:E2F1-responsive gene PPARA, a key mediator of fatty acid β-oxidation.

**Conclusions:** Our results unveil a new gene regulatory program by which E2F1-induced lncRNA-SLC16A1-AS1 forms a complex with its transcription factor that promotes cancer metabolic reprogramming towards the acquisition of a hybrid oxidative phosphorylation/glycolysis cell phenotype favoring BC invasiveness.

## Introduction

E2F1, a member of the E2F transcription factor family, controls networks of increasing importance in the context of cancer progression. Although this transcription factor activates tumor-suppressive pathways at early oncogenesis, upon disease progression unbalanced E2F1 activity is rewired to deregulated cancer networks that promote the emergence of aggressive tumor cells through the induction of epithelial-mesenchymal transition (EMT), invasiveness, resistance to therapy and metastasis 1,2. In particular, E2F1 abundance is the key event in superficial-to-invasive bladder cancer (BC) progression 3. The E2F1-governed networks underlying muscle invasive bladder cancers (MIBCs) are distinct from the ones supporting non-muscle invasive bladder cancers (NMIBCs). By integrating logic-based network modeling and gene expression profiles of cancer cell lines from E2F1-driven bladder tumors and patient cohorts displaying cancer aggressiveness, we identified tumor-type specific receptor signatures associated to EMT, where the combined action of highly expressed E2F1, TGFBR1 and FGFR1 triggers the most invasive phenotype 2,4. In addition, several other protein coding genes (PCGs), as well as miRNA-encoding genes 4-10 have been demonstrated as constituents of E2F1-activated prometastatic gene regulatory networks (GRNs). Interestingly, expression profiling in our MIBC progression model disclosed a cluster of E2F1-dependent long non-coding RNAs (lncRNAs). This observation adds a new layer of complexity to the landscape of E2F1 GRNs that warrants further investigation, especially in light of the fact that several lncRNAs have emerged as critical players of BC progression 11-15.

This class of non-coding RNAs includes all genes encoding RNA transcripts longer than 200 nucleotides, which are not translated into proteins. LncRNAs outnumber mRNA populations, present spatiotemporally-specific expression patterns and exhibit both, regulatory and structural roles via interacting with all kinds of molecules like DNA, RNA and proteins. They are frequently deregulated in many types of cancer 16, including BC 17, and can influence cancer initiation and/or progression 18,19. LncRNAs fine-tune a plethora of cellular processes at every level of cellular organization 20 by regulating genes that are either localized nearby on the same allele (cis-acting lncRNAs) or on a different chromosome (trans-acting lncRNAs) relative to the allele from which the lncRNA is transcribed 21. They show an overwhelming heterogeneity of mechanisms of action, which enables them to simultaneously regulate divergent targets and participate in heterogeneous processes. Allele-specific in cis mechanisms include, for instance, the modulation of chromatin states by acting as recruiters, tethers or scaffolds of epigenetic complexes. LncRNAs regulate mRNA transcription by acting as decoys of transcription factors, transcriptional co-regulators or RNA polymerase II inhibitors, and are structural components of nuclear bodies. They modulate alternative splicing, protein stability and/or protein subcellular localization and affect miRNomes by acting as sponges that sequester specific miRNAs away from their targets, as hosts of miRNAs genes, or as precursors of small mRNAs 22.

The combination of functional pleiotropy and high mechanistic versatility of lncRNAs decelerates their functional characterization and thus, this class remains largely enigmatic. For inferring the function(s) of a lncRNA, several rules of thumb have to be followed 23. First, the promoter region of a lncRNA gene is of paramount interest and, in terms of function, might be more important than the information encrypted in the actual lncRNA-coding region. In relation to PCGs, the primary encoding sequence of lncRNAs is less conserved than the promoter region 24. Additionally, in several cases, the act of transcription alone is sufficient for lncRNA function but the transcript itself is not necessary 25,26. Second, their subcellular localization indicates the mode of action, since nuclear lncRNAs contribute to chromatin remodeling and transcriptional regulation, while cytoplasmic-located are mainly involved in post-transcriptional processes, such as mRNA stability, miRNA function, or protein translation and signaling cascades 19,27. Third, the evolutionary patterns are taken into account, because lncRNA genes that have evolutionary conserved primary sequences, genomic positions and/or structures stand increased chances of being functional 23. Fourth, the genomic localization of lncRNAs in respect to their neighboring PCGs is relevant for their function, since genomic co-localization indicates co-functionality 28. In addition to these criteria, co-expressed PCGs that are negatively or positively associated with a lncRNA can also provide insights on the lncRNAs' role based on the guilt-by-association principle 29.

Considering these trends, we focused on the identification of E2F1-responsive lncRNAs that orchestrate BC progression and their phenotypic targets. Given that lncRNAs can act via regulating expression and/or function of nearby genes, we further hypothesized that lncRNAs in close genomic proximity with PCGs that are E2F1 targets possess a high probability of participating in E2F1-modulated GRNs. We established a pipeline consisting of high-throughput transcriptomic approaches, bioinformatics, *in silico*-based 3D structure modeling and molecular docking analyses, and functional validation, and found that SLC16A1-AS1 is a lncRNA that creates a divergent transcription unit with its associated PCG, the lactate transporter SLC16A1/MCT1. SLC16A1-AS1 is co-transcribed in conjunction with SLC16A1/MCT1 by E2F1 and subsequently, acts as co-activator of the transcription factor via direct lncRNA:protein interaction. This leads to the increase of glycolysis, mitochondrial respiration and fatty acid β-oxidation (FAO) upon SLC16A1-AS1:E2F1 complex-mediated upregulation of the metabolic effectors SLC16A1/MCT1 and PPARA. These data demonstrate that E2F1-mediated activation of the SLC16A1-AS1/MCT1 gene pair favors metabolic plasticity and reprogramming of bladder cancer cells, thereby facilitating cancer progression to invasive stages.

## Material and Methods

### Cell culture and treatments

The human BC cell lines RT-4, RT-112, T24 and UMUC-3 were purchased from the American Type Culture Collection (ATCC) and maintained as previously described 4. E2F1 activity in stably ER-E2F1-transduced cells was induced by 4-hydroxytamoxifen (4-OHT) at a final concentration of 1 μM.

### Genomic DNA extraction, RNA isolation, RT-PCR and qPCR analysis

Genomic DNA was extracted from cells using DNeasy kit (Qiagen) according to the manufacturer's protocol. RNA isolation, reverse transcription and qPCR were performed as described 10 using GAPDH or β-actin for normalization. For isolation of cytoplasmic and nuclear RNA, the Cytoplasmic & Nuclear RNA Purification Kit (Norgen Biotek Corp.) was used following the instructions. PCR primer sequences are shown in Table S1. RT-PCR was performed as described earlier 30.

### Cell lysates preparation, immunoblotting and immunofluorescence

Total cell lysates were prepared and quantified, and Western blots were performed as previously described 5. Proteins were probed with primary antibodies against E2F1 (Santa Cruz Biotechnology, sc-251 and Cell Signaling Technology, #3742), MCT1 (Santa Cruz Biotechnology, sc-365501) or β-actin (Sigma Aldrich, ac-15). The corresponding peroxidase-labeled secondary antibodies were detected using ECL Western blot reagents (Amersham Biosciences). Immunofluorescence assays were carried out as described in Goody et al. 9 using MCT1 (H-1) as primary and fluorescence-labeled anti-mouse A-488 as secondary antibody (Dianova, 715-5454-150).

### Plasmid construction and transfections

For construction of the pGL3-SLC16A1-AS1-Prom luciferase vector, a 767-bp fragment of the SLC16A1/SLC16A1-AS1 promoter containing the predicted E2F1 binding site was amplified from genomic DNA of T24 cells using the forward 5'-cggtggtaccGAACTTCCCGAGGTCACTGAACT-3' and reverse 5'-ccgtctcgagCGGCTGTTACCCAACTAACCGT-3'. The fragment was digested with *Kpn*I/*Xho*I and ligated into pGL3-basic. The insertion was verified by *Nhe*I/*Bam*H1 digestion. For the construction of promoter deletion mutants and corresponding wild-type control, SLC16A1/SLC16A1-AS1 promoter was excised from pGL3-SLC16A1-AS1-Prom luciferase vector using *KpnI* and *XhoI*, cloned into pGL4-basic and subjected to mutagenesis assays.

For generation of the pWPXL-SLC16A1-AS1 expression plasmid, the SLC16A1-AS1 gene containing the polyA-tail was synthesized *in vitro* and cloned in a pGE vector by Centic Biotec (Heidelberg). SLC16A1-AS1 fragment was excised from pGE-SLC16A1-AS1 vector using *Eco*RI and cloned in the *Eco*RI site of a pWPXL-vector. All constructs were verified by sequencing analysis (Sequence Laboratories Göttingen). The plasmids pcDNA3.1-E2F1, pcDNA3.1-E2F1 (E132) and pcDNA3.1-E2F1(-ΔTA) were described elsewhere 31. Transient transfections were performed using TurboFect^TM^ (Thermo Scientific) or Neon Transfection System (Invitrogen) according to the instructions.

### Mutagenesis and Deletions

Mutagenesis and generation of deletions of the promoter and lncRNA was performed using the QuikChange II Site-directed mutagenesis kit (Agilent) following the protocol. The primer sequences are shown in Table S1.

### Viral vectors and stable transduction

RT-4, RT-112 and T24 cells were transiently transduced with adenoviral vector Ad.ER-E2F1 1 at MOI 100. Cells stably containing E2F1 protein fused to the hormone-binding domain of the estrogen receptor (ER) were produced as described 9 and grown in medium containing 2 µg/ml puromycin (Sigma Chemical Co., St. Louis, MO). The ER-E2F1 fusion protein was activated by addition of 0.5 µM 4-hydroxytamoxifen (4-OHT). For the production of RT-4 clones expressing SLC16A1-AS1, pGE-SLC16A1-AS1 plasmid was restricted with *Xba*I/*Xho*I followed by Klenow and rSAP treatment. The released SLC16A1-AS1 insert was ligated into pLenti-Puro2AGFP 10 pre-treated with *Eco*RV. VSV-G-enveloped pseudotyped lentiviral vector was generated by cotransfecting HEK 293T cells with the expression construct and psPAX2 and pMD2.G packaging plasmids from Addgene as described previously 10. Stable clones were generated under puromycin selection.

### Generation of SLC16A1-AS1 knockout cells by CRISPR-Cas9

A pLKO5.sgRNA.Cas9.EFS.eGFP plasmid was used to stably express Cas9 in UMUC-3 as described by Richter *et al*. 10. Sequences of the guide RNA for SLC16A1-AS1 knockout were designed *in silico*: sgSLC16A1-AS1 forward: 5'-CGTTCGGGACACAACCATCG-3' and sgSLC16A1-AS1 rev: 5'-GTATTTATTTCAGGCCGGCG-3'. After annealing and phosphorylation, the oligonucleotides were cloned into the *Bsm*BI site of pLKO5.sgRNA.EFS.PAC. Following lentiviral vector production and transduction, single cell clones expressing guide RNAs were selected by puromycin to produce stable UMUC-3.CRISPR-Cas9-SLC16A1-AS1 knockout cells (UMUC-3-KO). Cas9 expression was validated by immunoblots with anti-Cas9 antibody (bd-20, sc-392737, Santa Cruz Biotechnology). SLC16A1-AS1 knockout was confirmed by PCR on genomic DNA using two primer pairs that are localized within the deletion or frame the deletion (Table S1).

### Chromatin immunoprecipitation

ChIP was performed as described 9. Protein-DNA complexes were immunoprecipitated using anti-E2F1 antibody (KH-95) or control IgG (Abcam). Input represents 10% of sheared chromatin prior to immunoprecipitation. Precipitated DNA fragments were amplified by PCR with 5'-CAGATTGCCTAGAGCTCGTCAGA-3' (forward) and 5'-CTCGTTTGCTTGTTCCAGTACCCA-3' (reverse) primers.

### RNA immunoprecipitation (RIP) assay

UMUC-3 and RT-4 cells (3×10^6^ cells per 145 mm dish) were transduced for 72 hours with adenoviral vectors expressing E2F1, GFP or shRNA against E2F1 at MOI 15; or transfected with pGE-SLC16A1-AS1 (wild-type or mutants) using TurboFect transfection reagent. RIP were either performed as native or cross-linked variant, as described previously 32,33. For native RIP, harvested cells were resuspended in lysis buffer (0.3% Triton X-100, 300 mM NaCl, 50 mM Tris-HCl pH7.5) supplemented with protease inhibitor (Roche Diagnostics), phosphatase inhibitor (Sigma-Aldrich) and RNase inhibitor (Thermo Scientific) at 4°C 32. Whole-cell extracts were collected from supernatants after centrifugation (14000 rpm, 10 min, 4°C). For immunoprecipitation, cell lysates (1 mg total protein input) were incubated overnight with anti-E2F1 (Cell signaling technology) or anti-normal rabbit IgG (Santa Cruz) followed by incubation with 40 µl Protein G Sepharose^TM^ 4 Fast Flow (GE Healthcare) for 1.5 hours at 4°C with gentle rotation. After centrifugation (600 × g, 3 min), supernatants were removed, Sepharose was washed three times with RIP buffer (150 mM KCl, 25 mM Tris-HCl, 0.5 mM DTT, 0.5% NP-40, protease, phosphatase and RNase inhibitor) and PBS. For cross-linked RIP, harvested cells (20×10^6^ cells) were cross-linked with 1% formaldehyde solution, followed by nuclear extraction and DNase digestion. For immunoprecipitation, one third of lysate was incubated overnight with anti-E2F1 or anti-normal rabbit IgG, followed by treatment with Protein G Sepharose^TM^ 4 Fast Flow (GE Healthcare) for 1.5 hours at 4°C with gentle rotation. After centrifugation, supernatants were removed, Sepharose was washed three times with immunoprecipitation buffer (150 mM NaCl, 10 mM Tris-HCL pH7.4, 1 mM EDTA, 1 mM EGTA pH8, 1% Triton X-100, 0,5% NP-40) and once with PBS 33.

Co-precipitated RNA (native and cross-linked) was eluted from the beads by proteinase K (Thermo Scientific) for 30 min at 55°C and subsequently purified using the NucleoSpin® RNA XS Kit (Macherey-Nagel). Reverse transcription was performed with First Strand cDNA Synthesis Kit and Random Hexamer Primer (Thermo Scientific). The resulting cDNA was amplified by qPCR or semi-quantitative PCR with primers specific for SLC16A1-AS1 and U6 (Table S1). The values were normalized by the fold-enrichment method, whereby the RIP signal of each E2F1-antibody-treated sample was divided through the signal of corresponding mock IP, which represents the background signal. Results are presented as fold increase in signal in E2F1 antibody-treated samples relative to the background signal 10.

### Luciferase reporter assays

To monitor promoter activity, cells were transiently transfected with indicated pcDNA3.1-E2F1 plus 1 µg pGL3-SLC16A1-AS1-Prom using Turbofect^TM^ (Thermo Scientific) according to the instructions. Luciferase activity was measured 36 hours after transfection using the Luciferase Reporter Assay System (Promega). Samples were normalized to total protein concentration in cell extracts. For mutation studies, 1×10^5^ UMUC-3 or RT-4 cells were transiently transfected with 0.4 µg or 0.8 µg pGL4-SLC16A1-AS1-Prom (BS full or BS deletion), 0.2 µg or 0.4 µg pGL4[hRluc75], 0.2 µg or 0.4 µg pcDNA3.1-E2F1 and/or pGE-SLC16A1-AS1 using Neon Transfection System (Invitrogen) according to the instructions. Luciferase activity was measured 72h post-transfection using Dual-Luciferase Reporter Assay System (Promega). Firefly-Luciferase activity (pGL4-SLC16A1-AS1-Prom) was normalized to Renilla Luciferase activity (hRLuc).

### Invasion and XTT proliferation assays

Boyden chamber assays and XTT proliferation assays were performed as described earlier 34.

### Measurement of extracellular fluxes

Cells were analyzed for glycolytic and mitochondrial function using Seahorse XF96 (Agilent; Santa Clara, CA, USA) with the corresponding kits (Glycolysis Stress Test Kit; 103020-100; Mito Stress Test Kit; 103015-100; Agilent). Ten-thousand cells were seeded into each well of an XF 96-well cell culture microplate in 80 µl culture medium and incubated overnight at 37°C in 5% CO_2_. The four corner wells were left with medium for background correction. For determination of oxidative respiration, the culture medium of cells to be investigated was replaced by 180 µl XF Base Medium containing 1 mM pyruvate, 2 mM glutamine, and 10 mM glucose, for determining glycolytic activity by 180 µl XF Base Medium without glucose and pyruvate, and incubated at 37°C for 60 minutes prior to measurement. The oxygen consumption (OCR) and extracellular acidification rates (ECAR) were measured with XF96 Extracellular Flux Analyzer. The ECAR values were determined by measuring the cells first without additives (basal measurement) and then one after the other glucose (10 mM), oligomycin (2 μM) and 2-deoxy-glucose (2-DG, 50 mM). Mitochondrial OCR values were detected in cells after basal measurement by adding oligomycin (2 μM), carbonyl cyanide 4-(trifluoromethoxy) phenylhydrazone (FCCP, 1 μM), and rotenone/antimycin A (each 0.5 μM).

The dependency, capacity and flexibility of cells oxidizing mitochondrial fuels like glucose (pyruvate), glutamine (glutamate) or long-chain fatty acids was determined by measuring mitochondrial respiration (OCR) of cells in the presence or absence of fuel pathway inhibitors (Mito Fuel Flex Test Kit; 103260-100; Agilent). Through the special inhibition of a pathway by UK5099 (glucose oxidation pathway inhibitor), BPTES (glutamine oxidation pathway inhibitor), or Etomoxir (long-chain fatty acid oxidation inhibitor), mitochondrial respiration follows via oxidation of both alternative substrates and allows calculation of the pathway dependence of cells to fulfill basal energy requirements. The substrate dependency indicates that the mitochondria of cells are unable to compensate the blocked pathway by oxidation of other fuels, while the ability to switch to other ways to promote mitochondrial respiration is expressed as fuel flexibility of the cells. Lack of flexibility indicates the mitochondrial desire of the corresponding fuel pathway to maintain basal OCR. The mitochondrial capacity in cells is calculated to cover the energy demand by inhibiting both alternative pathways. The results regarding glycolytic rate, capacity and reserve as well as the substrate dependencies were determined and shown, using Test Report Generators (Agilent).

### Microarrays

RNA from duplicates of T24.ER-E2F1, RT-4.SLC16A1-AS1 and UMUC-3-KO cell lines and respective controls was isolated and equal amounts of RNA were applied to AffymetrixGeneChip Human Transcriptome 2.0 Arrays (HTA 2.0) (Affymetrix). Background-corrected signal intensities were determined, processed and normalized using the Transcriptome Analysis Console (TAC, Affymetrix) and the SST-RMA algorithm. Significantly differentially regulated targets (p value < 0.05, |∆| ≥ 2-fold) in test samples versus corresponding controls were determined.

### Bioinformatics analyses

#### Detection of E2F1-responsive PCG/lncRNA gene pairs relevant for BC patient survival

The known human transcripts from GENCODEv27 assembly (hg38; https://www.gencodegenes.org/human/releases.html) were filtered for a transcript support level (TSL) up to 2 (TSL ≤ 2), uploaded to the Galaxy server 35 and merged (bedtools: closestBed) 36 with ChIP-seq-peaks data for E2F1 from the GTRD database (http://gtrd.biouml.org/) 37 of reprocessed and meta-clustered ChIP-seq of publically available experimental results. All PCGs and non-coding genes bearing ChIP-seq-identified binding sites of E2F1 within a promoter range of 1000 base pairs upstream up to 100 bp downstream of the transcription start site (TSS, GENCODE) were considered. These genes were compared with the differentially expressed genes obtained from T24.ER-E2F1 HTA 2.0 microarrays to identify genes (both PCGs and lncRNA genes) responsive to E2F1 activation. Detected potential candidate genes were analyzed for their topology and proximity to one another using the Galaxy server (bedtools: closestBed). Due to the ability of lncRNAs to affect nearby genes 38, their location relative to neighboring genes was also considered. To identify adjacent genes, we filtered for those that have TSSs less than 1000 bp apart from each other, which can be PCG/PCG, PCG/lncRNA and other pairs (e.g. involving pseudogenes and small non-coding RNAs). The received gene pairs were further classified according to the orientation of the transcripts as head-to-head, tail-to-tail and embedded, if located on opposite strands, and head-to-tail, overlap and embedded in case they are on the same strand 39. To estimate whether individual genes of a gene pair affect survival as an independent factor, Cox regression analysis was applied on patient expression data from TCGA database (GDC TCGA Bladder Cancer cohort (BLCA), HTSeq-FPKM data) using the 'coxph' function of the 'survival' package in R.

#### Search for SLC16A1-AS1 homologues across species

The entire nucleotide sequence of the human *SLC16A1-AS1* gene was obtained from the publicly available database NCBI's RefSeq (accession code: NR_103743). In order to detect putative *SLC16A1-AS1*-like regions in the genomes of other organisms, the database Ensembl (Release 86) 40 was queried by employing reciprocal BLASTn 41. Proximal promoter regions of the homologous* SLC16A1-AS1* genes were searched for evolutionarily conserved E2F1 binding elements by employing ConTra v2 42. Orthologous sequences corresponding to 250 base pairs upstream of the TSS of the *SLC16A1-AS1* gene from 10 species under investigation were extracted and aligned with MAFFT 43. The final multiple alignment was imported to ConTra v2 for analysis, the core and the similarity matrix threshold value was set at 0.95 and 0.85, respectively.

#### Functional analysis of SLC16A1-AS1-correlated genes

Genes whose expression levels are correlated (correlation |*r*| ≥ 0.4) with that of SLC16A1-AS1 in BC were downloaded from the TANRIC atlas 44 and subjected to GO analysis using the ClueGO plugin 45 of Cytoscape 46 as well as to Gene Set Enrichment Analysis (GSEA) 47 (Table S2).

#### MCT1 knockdown data analysis

Differential gene expression after shRNA-mediated knockdown of MCT1 was calculated using GEO2R software (https://www.ncbi.nlm.nih.gov/geo/geo2r/) using the GSE76675 study with a fold change cut-off of ±1.5 (*p* < 0.05).

#### Gene Set Enrichment Analysis

The list of differential gene expression was exported from TAC software, ranked by sign of FC divided by p value and applied to GSEA software (v.3.0) of the Broad Institute using the Hallmarks gene set.

### Structural analysis of lncRNA-E2F1-MCT1 promoter interactions

For predicting the interactions between SLC16A1-AS1, E2F1 and the MCT1 promoter, the following computational pipeline was applied:

#### Sequence retrieval and secondary structure folding of the lncRNA

The sequence of *SLC16A1-AS1* was retrieved from the NCBI's RefSeq (accession code: NR_103743). The thermodynamically stable secondary structure of the lncRNA was prepared by RNAfold server available on ViennaRNA web services 48.

#### Modeling and optimization of SLC16A1-AS1 and MCT1 promoter 3D structures

The initial 3D structure of the lncRNA was generated using 3DRNA v2.0 software tool 49. The structure was manually curated for missing interactions and bond length after applying CHARMM36 force field 50 using Biovia Discovery Studio 2017 (DS2017) software suite. The curated structure was further optimized using 'Smart Minimizer protocol' available in DS2017 for a maximum run of 20,000 steps with the 'Minimization RMS Gradient Tolerance' of 0.1 kcal / (mol x Å) to exit the minimization routine if the average gradient is less than or equal to the set cut-off. For modeling and optimization of the MCT1 promoter, we referred to the potential E2F1 binding site identified through our ChIP experiments. From the E2F1 binding site, we extracted the promoter sequence up to 25 bp in both directions. The 3D structure was prepared using DS2017 software suite.

#### Prediction and prioritization of E2F1 binding sites on the lncRNA

For prediction of possible binding sites between E2F1 and *SLC16A1-AS1*, we used catRAPID fragment module available on catRAPID omics server 51. Binding sites were prioritized based on the secondary structure conformations of protein and RNA. LncRNA fragments with large number of bases in the loop region were given priority over those having most of the bases in the stem region. Similarly, for protein, regions with high solvent accessible area were prioritized for interaction with lncRNA. The solvent accessibility of protein is calculated using NetSurfP-2.0 tool 52.

#### Molecular docking and molecular dynamics simulation of SLC16A1-AS1 and E2F1

Molecular docking of E2F1 and the most potential fragment of *SLC16A1-AS1,* as predicted by catRAPID omics server, was performed using PatchDock tool 53. Top 10 poses were refined with FireDock server 54. Further, the best docked pose was subjected to MD simulation study using GROMACS software package (4.5.3) 55 to analyze the interaction stability using AMBER force field. The entire simulations were done in the isothermal-isobaric ensemble, keeping both, the lncRNA fragment and E2F1 unconstrained throughout the simulation run. For the analysis of molecular interactions, a non-bonded cut-off was set to 10 Å and all the electrostatic interactions were calculated using particle mesh Ewald sums 56. Bonds between hydrogen and heavy atoms were constrained at their equilibrium length using LINCS algorithm 57. The production run of five nanoseconds (ns) was performed to study conformational changes during the simulation run time. All trajectories were saved after each 1 ps interval.

#### Prediction of lncRNA:E2F1 complex binding sites on the MCT1 promoter

Binding sites of the lncRNA-E2F1 complex on MCT1 promoter fragments were identified using PatchDock 53 and FireDock 54 tools. We explicitly specified E2F1 DNA binding domain residues to interact with the MCT1 promoter fragment. Based on the best pose, we further screened for lncRNA nucleotides interacting with the MCT1 promoter. Results were cross-validated using LongTarget tool predicting lncRNA DNA binding motifs in the promoter region based on the potential base pairing rules 58.

#### *In silico* RNA mutagenesis

Computational mutagenesis experiments were performed by deleting the RNA sequences corresponding to the loop-forming regions and the top binding sites between E2F1 and lncRNA. For each of the *in silico* mutants, potential binding sites between E2F1 and lncRNA were predicted by the catRAPID fragment module available on catRAPID omics server 51. Due to the long sequence of SLC16A1-AS1 (1522 bases) which is computationally very expensive, we used partial lncRNA (nts 141-612) sequence and structure for the interaction analyses with E2F1. For comparing the binding energy of docked complexes, we kept the length of lncRNA similar in all *in silico* mutagenesis studies by including additional nucleotides. The mutated lncRNA sequences were refolded using the RNAfold server available on ViennaRNA web services. For all lncRNA mutants, tertiary structures were prepared using 3DRNA v2.0 software tool 49 and optimized with 'Smart Minimizer protocol' available in DS2017. The optimized structures were used for molecular docking analysis with E2F1 using PatchDock and FireDock tools. We further analyzed and compared the binding pattern of top poses between E2F1 amino acid residues involved in the interaction with mutated lncRNAs.

### Statistical analysis

Statistical analysis of the differences between two groups was determined by paired Student's t test. P values less than 0.05 were considered as significant. (* *p* values < 0.05, ** *p* values < 0.01, *** *p* values < 0.001). All statistical test used in this study were two-sided.

## Results

### Identification of E2F1-responsive lncRNAs and their nearby PCGs in progressive bladder cancer

**Figure 1:**
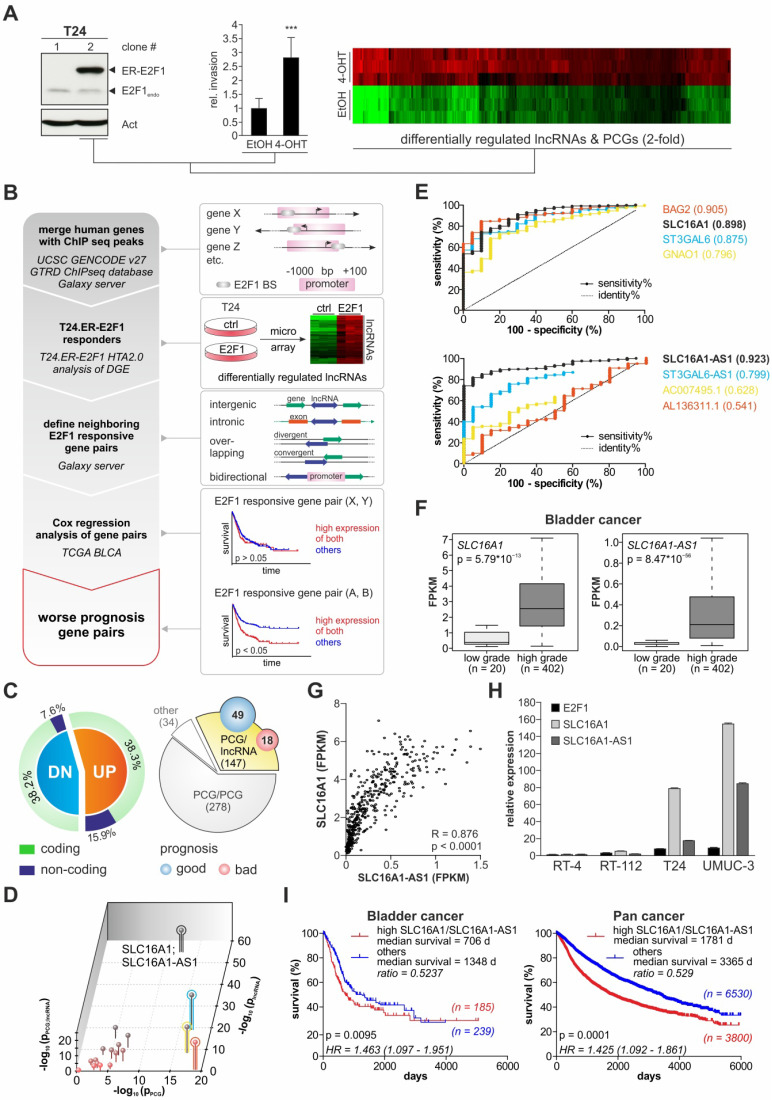
** Identification of E2F1-responsive lncRNAs and their nearby PCGs in progressive bladder cancer.** (**A**) Left: Immunoblot from two T24 clones depicting expression of the ER-E2F1 fusion protein. Endogenous E2F1 levels are also indicated. Center: Invasion assays of 4-OHT-treated versus ethanol-treated T24.ER-E2F1 clone 2 (n=3). Right: Corresponding heatmap of HTA 2.0 arrays showing differentially regulated (2-fold) lncRNAs and protein coding genes. (**B**) Workflow to identify E2F1-responsive lncRNAs and their neighbouring PCGs that are clinically relevant for BC patient survival. (**C**) Percentages of coding and non-coding (including lncRNA) genes upregulated (UP) or downregulated (DN) upon E2F1 induction (left panel). Number of PCG/lncRNA pairs, in which expression of any of the genes has an effect on BC prognosis based on Cox regression analysis (right panel). (**D**) Three dimensional plot showing the p values (x-axis: PCG alone, y-axis: lncRNA alone, z-axis: PCG/lncRNA) of the 18 gene pairs to classify TCGA patients into high and low grade BC by chance. (**E**) ROC curves of single genes from the best four separating gene pairs (see D) with indicated area under the curve (AUC). (**F**) Box plots of SLC16A1 and SLC16A1-AS1 expression in low versus high grade tumors in BC TCGA patients. High grade tumors show a significantly increased expression of both genes. (**G**) Spearman correlation of SLC16A1 versus SLC16A1-AS1 expression in BC patients indicating regulation by a common mechanism. (**H**) qPCR analysis shows higher expression levels of E2F1, SLC16A1 and SLC16A1-AS1 in invasive versus non-invasive bladder cancer cells. The levels are calculated relative to RT-4 cells. GAPDH or actin levels were used as normalization controls. (**I**) Kaplan-Meier analyses from TCGA data of the bladder cancer (left panel) and the Pan Cancer cohort (right panel) showing that patients with high SLC16A1/SLC16A1-AS1 co-expression (red line) have almost half the median survival time compared to all other patients (blue line). Number of patients in each group, log-rank test p values and Hazard ratios are depicted in the survival plots. Bar graphs are represented as means ± SD.

E2F1 promotes the invasiveness of urothelial bladder carcinoma 3,4,9,10. To study the role of this transcription factor in bladder cancer progression, we utilized a human tissue culture model consisting of various cell lines that differ in their invasive potential according to the endogenous E2F1 expression status. Inducible T24 clones for example, that stably express the ER-E2F1 fusion proteins show an increased invasive capacity following E2F1 activation by 4-OHT addition in comparison to the controls (Figure 1A). These clones were subjected to high-throughput HTA 2.0 transcriptome arrays to determine lncRNA genes and PCGs that are responsive to E2F1 upregulation (Figure 1A, right panel). In order to filter for lncRNAs that control BC progression in response to E2F1, we established the pipeline depicted in Figure 1B.

In detail, E2F1 ChIP-seq data from the GTRD database were used to identify putative genes that bind E2F1 via their promoter regions. These genes capable of binding E2F1 were filtered with the differentially expressed candidates derived from the T24.ER-E2F1 arrays to select for direct transcriptional targets of E2F1. While the majority (76.5%) of E2F1-regulated genes are PCGs, 23.5% are non-coding genes including lncRNAs (Figure 1C, left panel). These lncRNAs might be intergenic, intronic (sense or antisense), overlapping (divergent or convergent) or bidirectional relative to their neighbouring protein coding genes 59. Due to the importance of the genomic localization of lncRNAs in respect to nearby PCGs 28, we were particularly interested in those that are located in close genomic proximity (less than 1 kbp) to E2F1-responsive PCGs as highly probable to be involved in E2F1-governed GRNs. Overall, we identified 147 PCG/lncRNA gene pairs (Figure 1C, right panel, available at GitHub). Next, we performed Cox regression analysis on data of BC patients from the TCGA database to estimate if one or both genes of these PCG/lncRNA pairs can be associated with patient survival. The result showed that the expression of 18 gene pairs is associated with worse survival, whereas 49 of them were linked to a higher survival rate (Figure 1C, right panel). Furthermore, the majority of E2F1-responsive lncRNAs and related PCGs in these pairs are positioned in a head-to-head antisense direction. Given that such a genomic arrangement implies putative regulation via a shared promoter region, we postulated that the lncRNA is transcribed along with its corresponding PCGs upon E2F1 activation and, as a result, co-expression of each gene pair might have a mutual cooperative effect on patient survival. Therefore, we analyzed the potential of the 18 single genes and that of the nine corresponding gene pairs to classify patients into high and low grade BC tumors based on their expression levels (Figure 1D). Of those, four gene pairs had superior separation potential (z axis) compared to all others and moreover, revealed a role as potential prediction markers after analyzing ROC curves of these candidates. As best performing gene pair SLC16A1/SLC16AS-AS1 was identified, which is reflected by the separation of patients based on their expression levels (Figure 1F). As demonstrated by Figure 1G, SLC16A1-AS1 expression is also strongly correlated with that of SLC16A1/MCT1 in BC tumor patients, suggesting a common regulation. In agreement, qPCR in a panel of BC cell lines showed that SLC16A1-AS1 and SLC16A1/MCT1 are endogenously co-upregulated in high-E2F1 expressing T24 and UMUC-3 cells versus low-E2F1 RT-4 and RT-112 cells (Figure 1H).

Interestingly, Kaplan-Meier analysis revealed that SLC16A1/SLC16AS-AS1 co-expression is significantly associated with patient survival not only in BC but also across several other cancer types as evidenced in Pan Cancer cohorts (Figure 1I). Overall, these results suggest a role for the combined expression and possibly interaction between SLC16A1-AS1 and SLC16A1/MCT1 in bladder cancer aggressiveness and poor patient outcomes.

### SLC16A1-AS1 and SLC16A1/MCT1 induce cell invasiveness in bladder cancer

**Figure 2:**
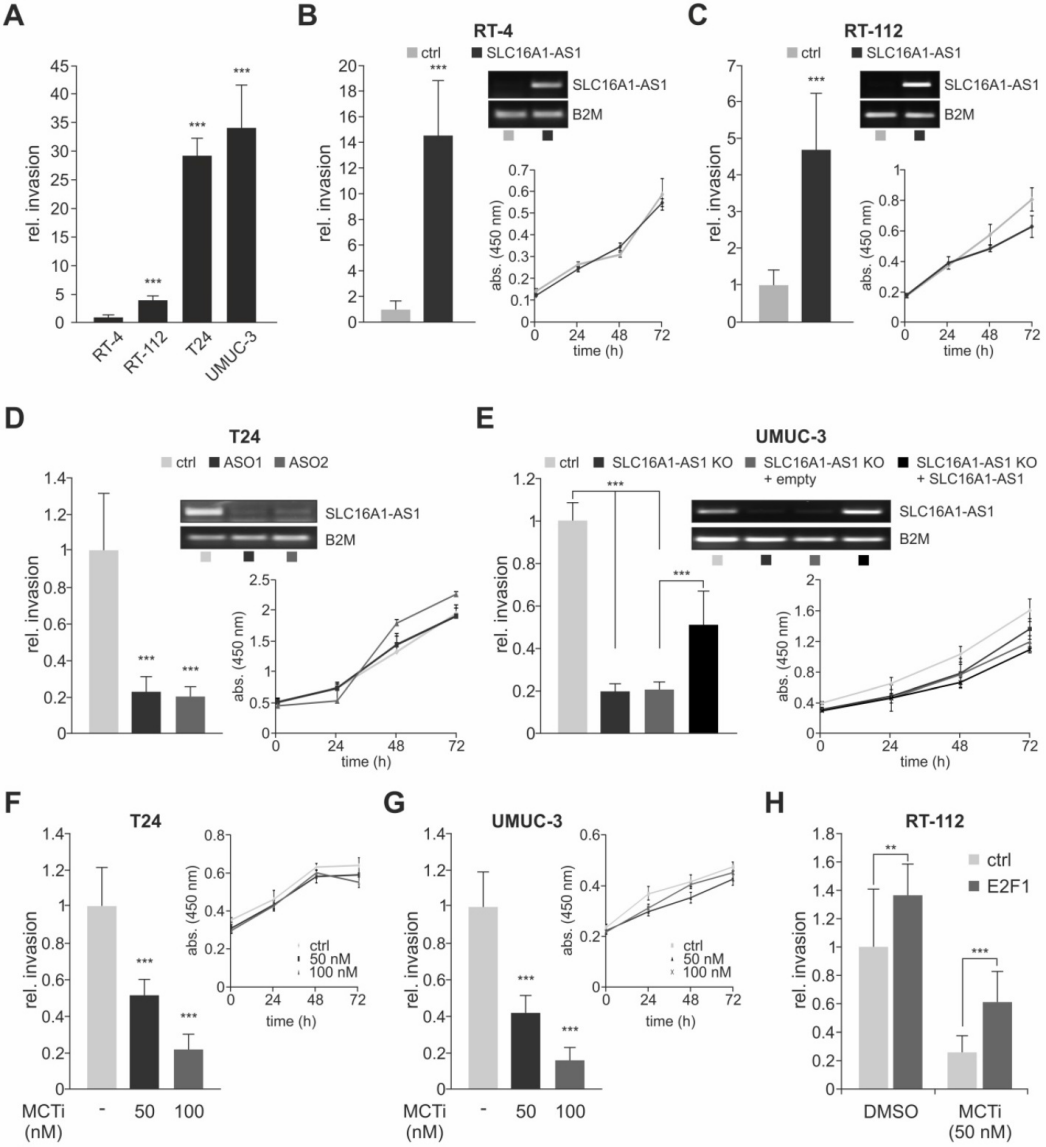
** SLC16A1-AS1 and SLC16A1 induce BC cell invasiveness.** (**A**) Invasion assays of RT-4, RT-112, T24 and UMUC-3 cells (n=3). (**B**) Left diagram: invasion assays in RT-4 cells transfected with SLC16A1-AS1-expressing plasmid relative to mock-transfected RT-4 controls. Right diagram: XTT assays in the same cells (n=3). SLC16A1-AS1 overexpression in these cells is verified with PCR (upper panel), while β2-microglobulin (B2M) was used as a normalization control. (**C**) Same as (B) for RT-112 cells transfected with SLC16A1-AS1 (n=3). (**D**) Same as (B) for T24 cells transfected with two different ASOs against SLC16A1-AS1 (n=3). (**E**) Left diagram: invasion assays in UMUC-3.Cas9 cells and their SLC16A1-AS1 knockout counterparts. Transient transfection of the knockout cells with an SLC16A1-AS1-expression plasmid, but not with the empty vector control rescued the invasive phenotype of UMUC-3-KO cells. Right diagram: XTT assays in the same cells (n=3). SLC16A1-AS1 expression in these cells is verified with PCR (upper panel) using B2M as a normalization control. (**F-G**) Matrigel assays (left diagram) and XTT assay (right diagram) in T24 (F) and UMUC-3 (G) cells treated with 50 or 100 nM of AR-C155858 inhibitor as compared to the DMSO-treated counterparts (n=3). (**H**) Matrigel assays in RT-112 cells transiently transfected with pcDNA3.1-E2F1 expression plasmid or empty pcDNA3.1 and subsequently treated with either AR-C155858 or DMSO (n=3). Bar graphs are represented as means ± SD.

The function of lncRNA-SLC16A1-AS1 in the context of bladder cancer is unknown. Although a recent study has attributed an anti-proliferative role to this lncRNA in lung cancer 60, they can have a diversified range of functions which is highly context-dependent 61,62. To shed light on its effects during bladder cancer progression, we used two low-invasive (RT-4 and RT-112) and two high-invasive (T24 and UMUC-3) BC cell lines (Figure 2A and Figure S1A) and monitored whether overexpression or knockdown of SLC16A1-AS1 affects their invasion and proliferation capacity. Addition of SLC16A1-AS1 to RT-4 (Figure 2B and Figure S1B) and RT-112 (Figure 2C and Figure S1C) tumor cells leads to a strong increase of invasiveness. In contrast, XTT assays in the same cells revealed that proliferation is unaffected by this lncRNA (Figure 2B and 2C, right panels). Vice versa, treatment of invasive T24 cells with antisense oligonucleotides (ASO) against SLC16A1-AS1 decreased invasiveness without altering cell proliferation (Figure 2D and Figure S1D). A similar effect was achieved upon CRISPR-Cas9-mediated knockout of the lncRNA in aggressive, high SLC16A1-AS1-expressing UMUC-3. Notably, subsequent transient transfection of knockout cells with a SLC16A1-AS1-expression plasmid rescued the invasive phenotype, but did not significantly affect their proliferative potential (Figure 2E and Figure S1E).

Unlike the poorly characterized lncRNA-SLC16A1-AS1, SLC16A1/MCT1 is a well-known lactate transporter that exhibits oncogenic behavior 63-65. A main metastasis-promoting process in which SLC16A1/MCT1 is involved includes, but is not limited to, aerobic glycolysis known as the Warburg effect 66. During aerobic glycolysis, the glucose-derived pyruvate, instead of entering mitochondria to be further catabolized via the tricarboxylic acid cycle (TCA), is converted to lactate in the cytoplasm independent of the oxygen availability. SCL16A1/MCT1 is one of the main transporters that handle lactate excess, leading to acidification of the tumor microenvironment 67. Treatment of high malignant T24 and UMUC-3 with MCT1 inhibitor AR-C155858 which blocks lactate export 68 results in a reduction of invasiveness that is dose-dependent (Figure 2F,G and Figure S1F,G), supporting recent findings in other BC cell lines 69. In this setting, proliferation rates remain unaffected by blockage of MCT1 transporter, in agreement with studies showing that MCT1 silencing does not affect cancer cell growth 66. We further demonstrate that the effect of SLC16A1/MCT1 in bladder cancer is E2F1-dependent. As shown in Figure 2H, Boyden-chamber assays of RT-112 cells transfected with pcDNA3.1-E2F1 and subsequently treated with AR-C155858 revealed a decreased invasive growth in response to the MCT1 inhibitor compared with the DMSO treated control (Figure S1H). These data indicate that inhibition of SLC16A1/MCT1 severely impairs E2F1-mediated invasiveness in BC cells. In conclusion, both lncRNA-SLC16A1-AS1 and SCL16A1/MCT1 have the potential to regulate bladder cancer towards an invasive phenotype.

### SLC16A1-AS1 and SLC16A1/MCT1 are co-transcribed by a conserved bidirectional E2F1-regulated promoter

**Figure 3:**
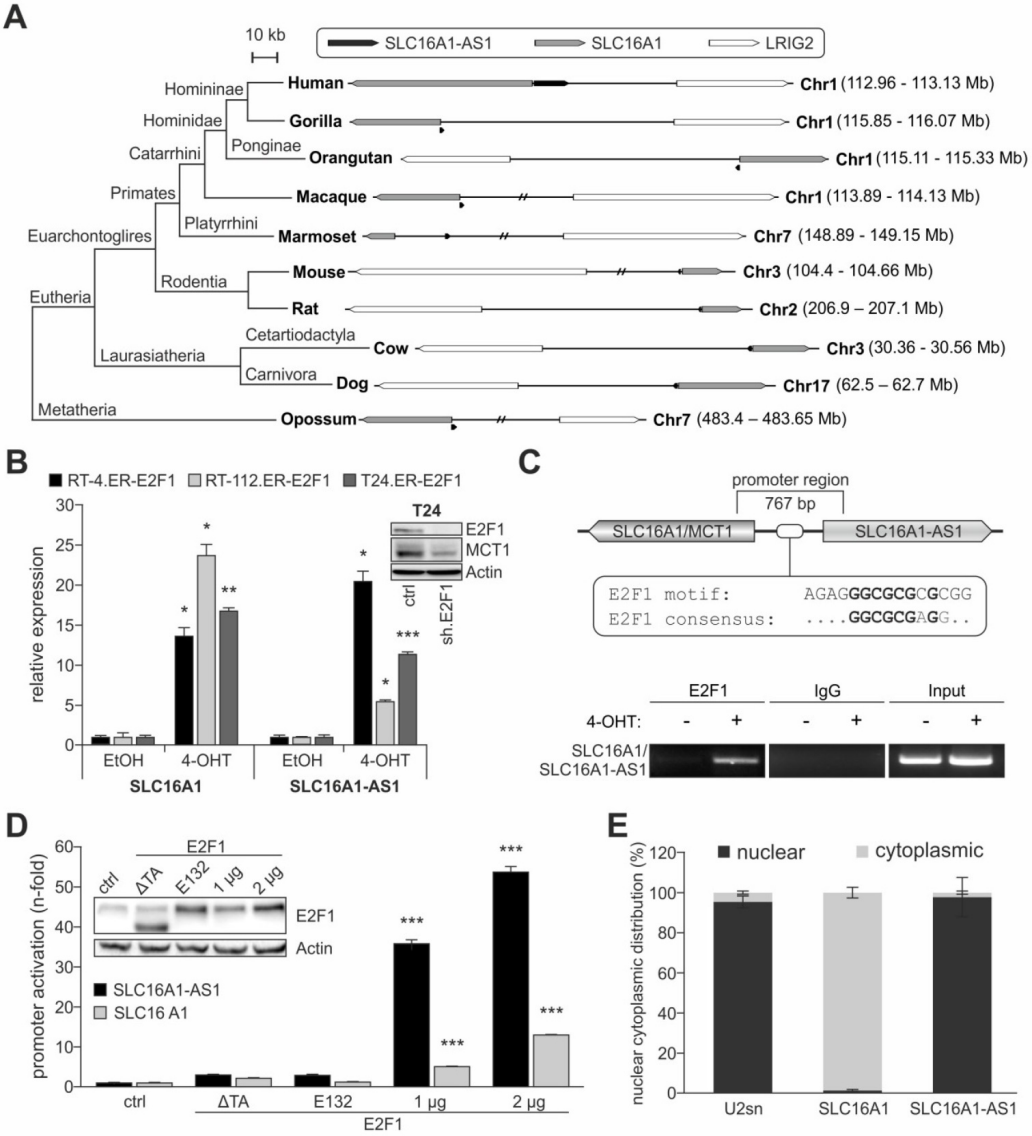
** E2F1 transactivates the SLC16A1-AS1/SLC16A1 gene pair.** (**A**) Identification of SLC16A1-AS1 homologs and phylogenomic analysis of the SLC16A1-AS1/SLC16A1 gene pair across vertebrates. LRIG2 functions as relative genomic anchor point. (**B**) qPCR to evaluate SLC16A1/MCT1 and SLC16A1-AS1 transcript levels in RT-4, RT-112 and T24 cells after 4-OHT-induced expression of E2F1, as compared to their ethanol-treated counterparts (n=3). Top right: Western blot of E2F1 and MCT1 after E2F1 knockdown. Actin was used as a loading control. (**C**) Top: Diagram of the promoter region shared between SLC16A1/MCT1 and SLC16A1-AS1. The predicted E2F1 binding site is indicated. Bottom: ChIP assay in T24.ER-E2F1 cells treated with 4-OHT (+) or EtOH control (-), in presence or absence of anti-E2F1 antibody. IgG was used as negative control. The input corresponds to 10% of the total amount of sonicated chromatin. (**D**) Relative luciferase activities after cotransfection of pGL3-SLC16A1-AS1-Prom luciferase vector with 1 or 2 µg of wild-type E2F1 in T24 cells. The pcDNA3.1-E2F1 (E132) and pcDNA3.1-E2F1 (ΔTA) plasmids which express transactivation-deficient forms of E2F1, as well as empty pcDNA3.1 were used as negative controls (n=3). Fold changes were calculated relative to controls (set as 1). E2F1 expression was confirmed by immunoblot with actin as loading control. (**E**) Relative SLC16A1/MCT1 and SLC16A1-AS1 mRNA levels in nuclear and cytoplasmic extracts of T24 cells. U2sn was used as nuclear localization control. Bar graphs are represented as means ± SD.

The human lncRNA-SLC16A1-AS1 gene is located in antisense direction from the SLC16A1/MCT1 gene on chromosome 1, at 1p13.2-p12. Several lncRNAs are positionally conserved with their nearby PCGs. Characterization of these lncRNA and protein coding genes has shown that they regulate each other's expression and influence the aggressive phenotype of cancer cells *in vitro* in a similar fashion 28. Given that positional conservation of lncRNA genes to their associated PCGs implies a functional connection 28, we investigated if this divergent head-to-head arrangement of both genes is conserved among species. Sequence-based comparisons in accordance to current rules used for identifying lncRNA homologues across species 70,71 revealed the existence of putative SLC16A1-AS1 homologues in primates (chimpanzee, macaque, marmoset), rodents (mouse, rat), eutheria (cow, dog, horse) and metatheria (opossum) (Figure S2). Interestingly, the putative lncRNA homologues have a conserved position and orientation with respect to SLC16A1/MCT1, and their upstream promoters are bidirectional to SLC16A1 (Figure 3A). These common promoters bear putative E2F1-responsive elements (Figure S3; predicted transcription factor binding sites are marked yellow), which is also suggesting that the cross-talk between E2F1 and the non-coding and protein-coding DNA sequences is evolutionary conserved. Overall, the gene arrangement between SLC16A1-AS1 and SLC16A1/MCT1 and its putative E2F1-responsive promoter shows high similarity across mammals.

The conservation of this genetic arrangement could serve a need for these molecules to be expressed simultaneously by E2F1. In this respect, we analyzed whether SLC16A1-AS1 and SLC16A1/MCT1 are co-transcribed in bladder cancer cells. Induction of E2F1 in RT-4, RT-112 and T24 led to a significant elevation of lncRNA and lactate transporter transcript, suggesting that they are co-upregulated via a common promoter (Figure 3B). ChIP assays shown in Figure 3C and luciferase reporter assays done in T24 cells (Figure 3D) further confirmed the ability of E2F1 to bind to the shared promoter region, thereby inducing head-to-head transcription of both genes. We then examined where SLC16A1-AS1 and SLC16A1/MCT1 localize in tumor cells and observed that SLC16A1/MCT1 mRNA and protein both reside in the cytoplasm, while the lncRNA remains in the nucleus (Figure 3E and Figure S4).

These results demonstrate common conservation, expression and regulation profiles for lncRNA-SCL16A1-AS1 and its associated PCG that are indicative for their functional connection. However, considering their distinct subcellular localization, we excluded the possibility that SLC16A1-AS1 exerts its function(s) via physical association with MCT1. Rather, its nucleus-restricted expression implies a possible role in transcriptional regulation or chromatin remodeling 19,27.

### LncRNA-SLC16A1-AS1 physically interacts with E2F1 and enhances SLC16A1/MCT1 expression

LncRNAs have been characterized as biochemically versatile polymers due to their potential to participate in a multitude of functions through divergent mechanisms. One of the main mechanisms is that they act as transcriptional regulators 61. Notably, it was recently shown that E2F1 controls the expression of PCGs whose products in turn, physically associate with E2F1 to enhance transcription of prometastatic targets 6,9,72. With this in mind, we hypothesized that this could be also the case for the nucleus-localized lncRNA-SLC16A1-AS1, which may interact with E2F1 to fine-tune transcription of E2F1-regulated targets.

**Figure 4:**
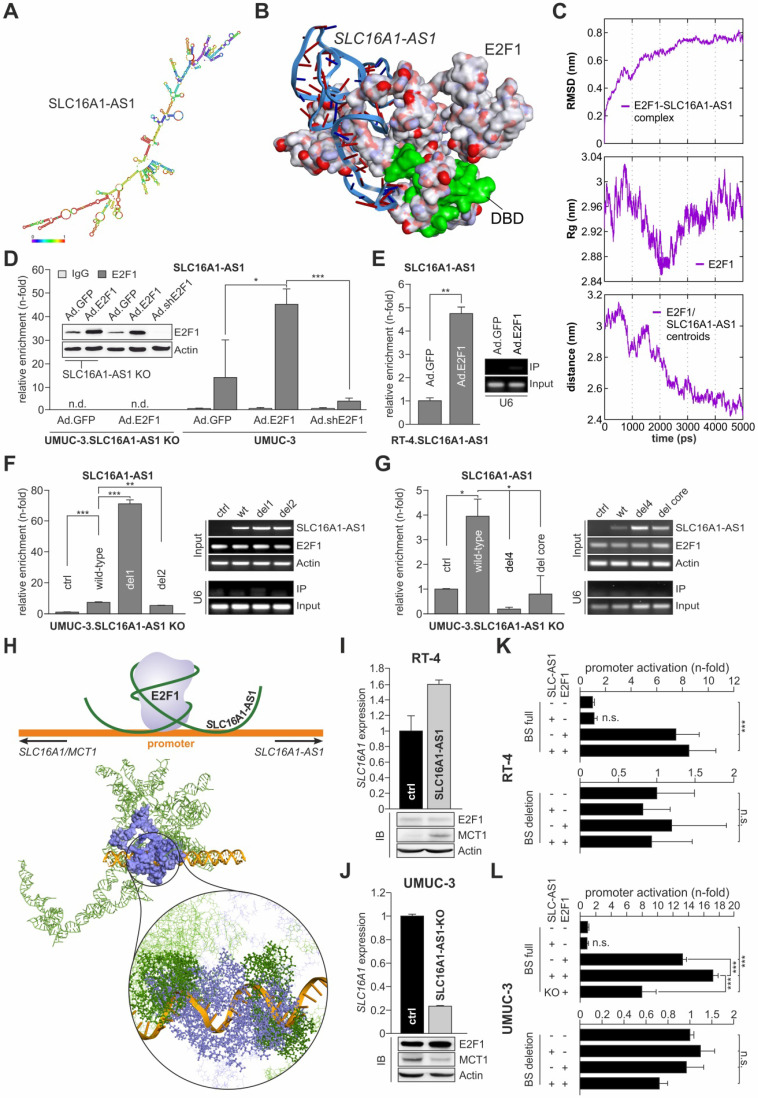
** E2F1 interacts with SLC16A1-AS1 and enhances MCT1 expression.** (**A**) Thermodynamically most stable secondary structure of full-length SLC16A1-AS1. Nucleotides are colored based on base pair probability scale. (**B**) Best docking pose of the SLC16A1-AS1 lncRNA fragment (ribbon model) with E2F1 (surface model). The DNA binding domain (DBD) of E2F1 is shown in green. (**C**) Molecular dynamics simulation graphs of the SLC16A1-AS1:E2F1 complex over a time period of 5 ns. Left: The root mean square deviation (RMSD) profile of the backbone atoms indicates that the SLC16A1-AS1:E2F1 complex is stable after ~3 ns compared to the initial structures. Center: Simulation of the overall compactness (radius of gyration, Rg) representing the stability of E2F1 in the complex. In the last 2 ns E2F1 recovers its native conformation in the complex. Right: The graph shows the decreasing distance between E2F1 and SLC16A1-AS1 centroid over time implying a stable complex formation of E2F1 and the lncRNA. (**D**) RIP assay of UMUC-3 cells transduced with Ad.E2F1-, Ad.shE2F1- or Ad.GFP. The E2F1:RNA complexes were immunoprecipitated with either anti-E2F1 antibody or control IgG antibody (n.d.; not detected). qPCR showed a SLC16A1-AS1 enrichment in the UMUC-3-E2F1 transfected cells, but not in the UMUC-3.shE2F1- or mock-transfected cells. (**E**) RIP assay of RT-4 cells transduced with Ad.E2F1 or Ad.GFP. (**F**) RIP-assay on deletion mutants (del1 and del2) versus wild-type (wt) SLC16A1-AS1, using anti-E2F1 antibody in UMUC-3-KO cells. Left diagram: qPCR enrichment of RNA sequences. Right, upper panel: PCR detection of lncRNA sequences and E2F1 in the RIP input. Actin was used as a loading control. Right, lower panel: PCR detection of U6 in the anti-E2F1 immunoprecipitate. U6 was used as a control for specificity of E2F1-lncRNA binding (**G**) Same as (F), for del4 and del core lncRNA mutants. (**H**) Top: Graphical sketch of the E2F1:SLC16A1-AS1-complex interacting with the composite binding site on the SLC16A1/SLC16A1-AS1 promoter region. Bottom: Model of the most stable interaction pose between the E2F1:SLC16A1-AS1 complex (receptor) and the MCT1 promoter (ligand), suggesting that the lncRNA (green) stabilizes the binding of E2F1 (blue) by interacting with both, the protein and the promoter (orange). (**I-J**) RNA and protein levels of SLC16A1/MCT1 in (I) RT-4 cells stably overexpressing SLC16A1-AS1 and (J) UMUC-3-KO cells versus their corresponding controls. Actin was used as loading control and bar graphs are represented as means ± SD. (**K-L**) Luciferase assay of wild-type (BS full) or mutated (BS deletion) MCT1 promoter constructs co-transfected with E2F1 and SLC16A1-AS1 expression plasmids (SLC16A1-AS1) in RT-4 (K) and UMUC-3 (L). Fold changes were calculated relative to untransfected controls (set as 1). KO; SLC16A1-AS1 knockout, n.s.; not significant. Experiments were performed at least twice.

To investigate potential interaction sites between SLC16A1-AS1 and E2F1, we performed computational secondary and tertiary structure modeling of the lncRNA followed by molecular docking studies with E2F1. The thermodynamically most stable secondary structure of SLC16A1-AS1 (Figure 4A, minimum free energy (MFE) -525.60 kcal/mol) was designed using RNAfold server available on ViennaRNA web services. Probable E2F1 binding sites on SLC16A1-AS1 were predicted using catRAPID fragment tool, which first divides protein and lncRNA into small sequences and then predicts the binding propensity between them in an iterative manner. Interestingly, all identified SLC16A1-AS1 binding regions (151-323 bp) are mainly interacting with amino acid residues between the dimerization and transactivation domain of E2F1 (Table S3). Moreover, we used parameters from secondary structures to prioritize binding sites of the lncRNA-E2F1 complex. In particular, loop regions were given priority over stems in the best secondary structure conformation for the lncRNA, whereas the solvent accessibility of amino acid residues was considered for E2F1. Our results predict a loop region of SLC16A1-AS1 presenting most nucleotides for binding between base pairs 172-233, while the E2F1 fragment encompassing amino acid residues 312-363 exerts the maximum solvent accessibility (absolute solvent accessibility (ASA) = 92.69) (Table S4) as best interacting sites among all possible fragments.

To strengthen our prediction, we analyzed the interactions between SLC16A1-AS1 and E2F1 at a tertiary structure level. For this, the initial 3D template of the complex was generated using 3DRNA v2.0 software tool 49, which is based on a fragment assembly approach to build RNA 3D structures utilizing the information from the secondary structure folding. The initial 3D structure was manually cross-checked for bond length, missing interactions, and additionally optimized using 'Smart Minimizer protocol' available in DS2017 using CHARMM force field 73 (Figure S5). Due to the limitation of molecular docking software to operate with ligand molecules, we extracted a small 3D fragment (172-233 bp) from the full-length SLC16A1-AS1 structure according to the identified best probable lncRNA:E2F1 interaction from the previous analysis (Table S4). This sequence was extended ten bases in both directions and by applying our earlier published 3D model of E2F1 6,9,72 molecular docking of lncRNA and protein fragments was performed using PatchDock tool.

The top 50 poses obtained between the SLC16A1-AS1 fragment and E2F1 were refined and analyzed for their interaction patterns. We found an interesting match between the most favouring thermodynamic pose of the E2F1-lncRNA complex (ΔG= -17.81 kcal/mol) and the binding sites predicted by catRAPID server on the E2F1 surface based on sequence information (Figure 4B). To verify complex stability, molecular dynamics simulation was performed with GROMACS software package (version 4.5.3) and AMBER force field, widely used for protein and nucleic acid simulation. We calculated the root mean square deviation (RMSD) of the back bone atoms with reference to their initial conformation, radius of gyration (Rg) of the protein, and the distance between lncRNA fragment and E2F1 over a 5 ns simulation run (Figure 4C). Our results predicted that lncRNA-SLC16A1-AS1 forms a stable complex with E2F1. To experimentally validate this predicted physical interaction, we conducted RIP assays (Figures 4D and E). As shown in Figure 4D, UMUC-3 cells were transduced with E2F1, shE2F1 or GFP expressing adenoviral vectors and E2F1-RNA complexes were immunoprecipitated with anti-E2F1 antibody. RNA was purified from the precipitates and amplified by qPCR with primers specific for SLC16A1-AS1. Using the fold-enrichment method 10, we found, in comparison to IgG, a clear SLC16A1-AS1 enrichment (14-fold) in the E2F1 precipitates of control-transduced UMUC-3 (Ad.GFP), which is indicative for a direct interaction of endogenously expressed SLC16A1-AS1 and E2F1. Addition of E2F1 in UMUC-3 (Ad.E2F1) is accompanied by an increased enrichment of the lncRNA (45-fold), whereas shRNA-mediated E2F1 depletion (Ad.shE2F1) shows reduced amounts of SLC16A1-AS1 (4-fold) compared to the Ad.GFP control. In contrast, we could not detect any lncRNA in UMUC-3-KO cells neither in Ad.GFP nor Ad.E2F1 transduced cells. In a similar manner, transduction of low E2F1-expressing RT-4 cells with Ad.E2F1 led to a more than 4-fold enrichment of SLC16A1-AS1 compared with controls (Figure 4E). These data underline a stable complex formation with E2F1.

Next, we attempted to identify the specific lncRNA regions that mediate binding to E2F1. To this end, we generated several *in silico SLC16A1-AS1* deletion mutants based on our computational interaction model. One series of larger deletions (> 60 bp) encompass the previously top-predicted binding regions between E2F1 and the lncRNA: *del1* (∆172-233), *del2* (∆262-323), and *del3* (∆172-233 + ∆262-323). In a second step, we focused on smaller loop-forming sequences within the identified E2F1-interacting lncRNA fragment (172-233 bp), namely *del4* (∆193-209), *del5* (∆172-175), and *del6* (∆172-175 + ∆193-209) (Figure S6A). To estimate the effect of the deletions on complex formation, first, we refolded the 2D structures of the lncRNA mutants and calculated their minimum free energy (MFE). In all cases, we found structural changes accompanied by alterations in the number of hairpins, interior- and multi-loops, and a general reduction of the MFE, indicating an impairment of binding affinity of lncRNA to E2F1 (Figure S6B, Table S5). In a more detailed view, we performed, as described above, molecular docking studies of the mutated lncRNA fragments with E2F1 and analysed the top interaction poses. In this way, we could compare on one hand, the RNA-interacting amino acid residues and on the other hand, the binding energy of wild-type SLC16A1-AS1 and the generated deletion mutants (Figure S7, Table S5). Most interestingly, we found that although *del1* did not show any interaction with amino acid residues from E2F1, it could form a thermodynamically more stable complex (ΔG_wild-type_= -35.68 kcal/mol vs ΔG_del1_= -43.67 kcal/mol). On the contrary, deletion of a single stem-loop (*del4*) within this region showed few interactions with amino acids similar to the wild-type, but a potentially weaker complex formation (ΔG= -33.47 kcal/mol). Yet, removal of four nucleotides at the end of the top-interacting SLC16A1-AS1 fragment (*del5*) was predicted to maintain most E2F1-associating RNA structures and result to a more stable complex (ΔG= -57.29 kcal/mol), accordingly. To validate the computational data, we performed RIP assays after transfections of UMUC-3-KO cells with wild-type SLC16A1-AS1 or the *del1*, *del2* and *del4* mutants (Figure 4F, G). Importantly, *del1* was strongly enriched in the immunoprecipitates, confirming a stronger complex formation. Our analysis suggests that deletion of the region between nucleotides 172-233 may result in the binding of lncRNA to a very distinct site of E2F1 compared to the wild-type. The binding of *del2* to E2F1 was only slightly decreased (Figure 4F), while in the case of the smaller stem-loop mutant *del4*, it was strongly reduced. Additionally, we generated a deletion representing the stem-loop core (*del core,* ∆198-203) which could partially recover the loss of bound SLC16A1-AS1 (Figure 4G), indicating an important function of this lncRNA stem-loop as a binding component for E2F1. Taken together, these data not only confirm the predictions based on the binding energies, but also demonstrate that the association of E2F1 with SLC16A1-AS1 depends primarily on the structure of this lncRNA.

Given that SLC16A1-AS1 physically associates with E2F1 and MCT1 is essentially an E2F1 target, we wondered if SLC16A1-AS1 functions in a cis-acting manner to further regulate transcription of its PCG. For this purpose, we generated in a first step a three dimensional conformation from a part of the above described SLC16A1/SLC16A1-AS1 promoter (Figure 3C), comprised of 25 additional nucleotides in each direction around the E2F1 motif using the 'Build and Edit Nucleic Acid' protocol in DS2017. Further, the best interaction pose of E2F1 and the SLC16A1-AS1 fragment (Figure 4B) was superimposed on the full-length lncRNA structure to create a complete receptor molecule. Subsequently, we predicted the interactions of the SLC16A1-AS1:E2F1 complex (receptor) with the MCT1 promoter fragment (ligand) using PatchDock and FireDock tools. After retrieving and evaluating the top docking poses, we observed that the E2F1 DNA binding domain interacts with the previously verified TF binding site on the SLC16A1/SLC16A1-AS1 promoter. More interestingly, we also found that SLC16A1-AS1 interacts with various nucleotides on both sites of the E2F1 motif. Accordingly, we applied the LongTarget tool 58 to identify potential lncRNA-promoter binding sites with the possibility to form DNA-RNA-triplexes depending on the base pairing rules (Table S6). We observed a convincing accordance between the results at sequence and structure level and beyond, corroborating the promoter binding of SLC16A1-AS1 at a 3D molecular interaction level. In this context our results suggest that SLC16A1-AS1 stabilizes the binding of the transcription factor to the SLC16A1/SLC16A1-AS1 promoter through the establishment of DNA-RNA-triplexes close to the E2F1 motif (Figure 4H) and supporting a function as transcriptional co-regulator. To verify this conclusion, SLC16A1 levels were measured in RT-4 and UMUC-3 cells after ectopic expression or knockout of SLC16A1-AS1, respectively. The results in Figure 4I and 4J demonstrate clearly enhanced MCT1 mRNA and protein levels upon SLC16A1-AS1 overexpression in RT-4 and reduction in UMUC-3-KO cells compared to their corresponding controls. These results correlated with the activity of the SLC16A1 promoter in these cell lines. In particular, luciferase assays showed enhanced promoter activation upon co-expression of E2F1 and SLC16A1-AS1, whereas lncRNA knockout in UMUC-3 significantly reduced promoter activity. In contrast, no changes were detectable after deletion of the E2F1-SLC16A1-AS1 binding site (Figure 4K, L). In summary, our findings suggest that SLC16A1-AS1 interacts with E2F1 to enhance MCT1 expression.

### SLC16A1-AS1 induces metabolic reprogramming in bladder cancer

**Figure 5:**
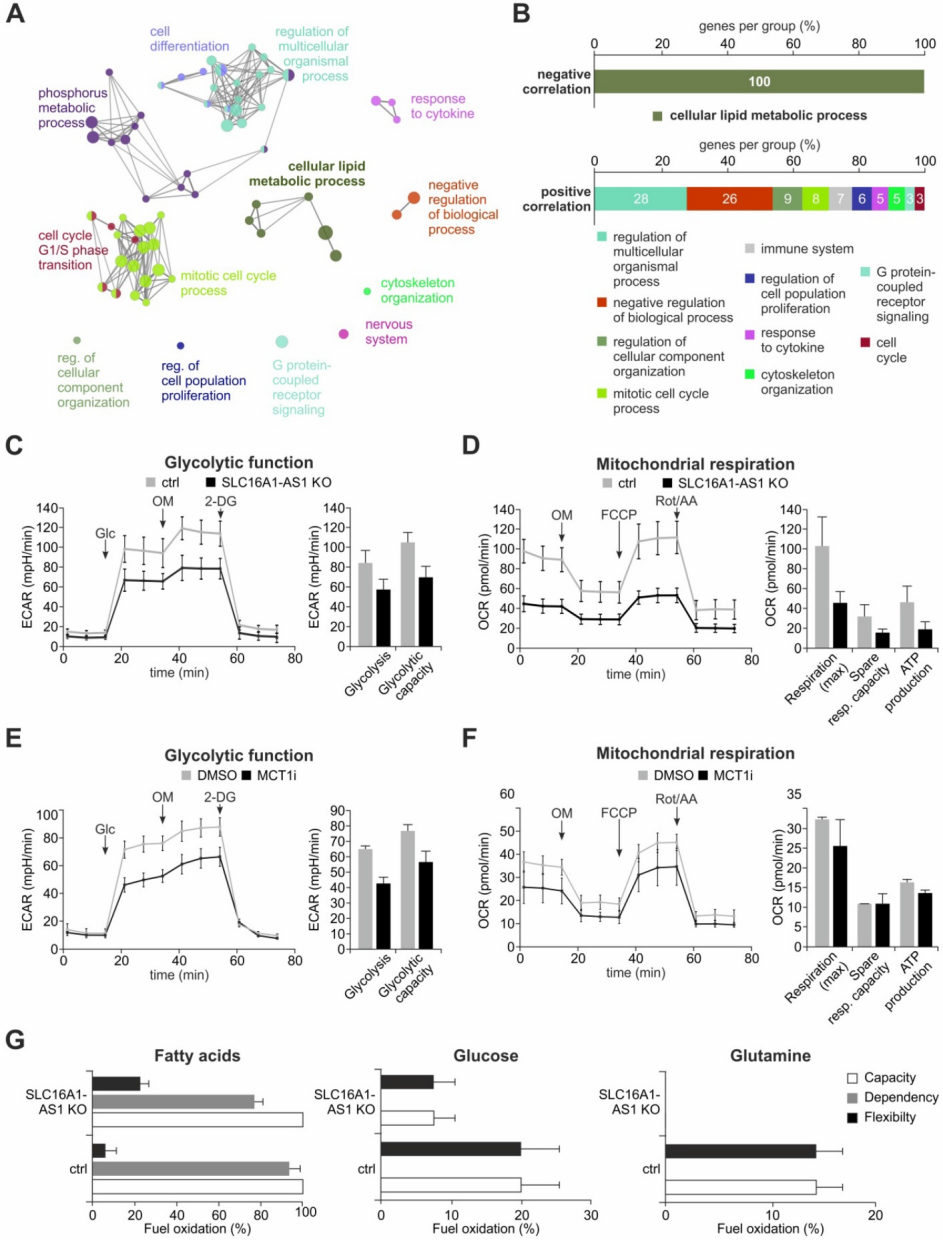
** SLC16A1-AS1 induces metabolic reprogramming of BC cells by enhancing glycolysis, OXPHOS and FAO.** (**A**) Cytoscape GO analysis of genes that show a positive or negative correlation with SLC16A1-AS1 expression in BC patients. (**B**) Percentages of clustered genes associated with each process. Negatively correlated genes exclusively cluster with lipid metabolism, while the positively correlated genes are implicated in divergent processes. (**C**) Seahorse XF Glycolysis Stress Test in UMUC-3-KO cells in comparison with UMUC-3.Cas9 control. Measurements were performed in triplicates (left diagram). Mean ECAR ± SD values for glycolysis and glycolytic capacity are depicted (right diagram). (**D**) Seahorse XF Cell Mito Stress Test in UMUC-3-KO versus UMUC-3.Cas9 cells. Measurements were performed in triplicates. Mean OCR ± SD values for maximal respiration, spare respiration capacity and ATP production are shown. (**E, F**) Glycolysis (E) and Mito Stress Test (F) in UMUC-3 cells treated with either 100 nM inhibitor AR-C155858 or DMSO as control. Measurements were performed in triplicates. (**G**) Seahorse XF Mito Fuel Flex Test on UMUC-3-KO versus UMUC-3.Cas9 cells. Percent capacity, dependency and flexibility values for fatty acids (left diagram), glucose (center diagram) and glutamine (right diagram) were measured in triplicates. Glc; glucose, OM; oligomycin A, 2-DG; 2-deoxy-glucose, Rot/AA; rotenone/antimycin A.

To gain further insights on the potential biological functions of SLC16A1-AS1 in bladder cancer, we applied a guilt-by-association *in silico* approach 29 to identify genes whose expression correlates with the levels of SLC16A1-AS1. To this end, we used data available from TANRIC atlas to retrieve a list of PCGs that are either positively or negatively correlated (|r| ≥ 0.4, n = 2236 genes) with SLC16A1-AS1 in BC patients and subjected them to GO-analysis using ClueGO in Cytoscape. The results obtained demonstrate that the genes connected to SLC16A1-AS1 expression are implicated in a variety of cellular processes such as mitotic cell cycle, cell differentiation and phosphorus-metabolic process (e.g. regulation of protein phosphorylation or signal transduction) (Figure 5A, B; Figure S8A). Interestingly, all negatively correlated genes have been clustered exclusively with lipid metabolism (Figure 5B; Table S2 and Figure S8B).

The observed association of SLC16A1-AS1 expression with metabolism-related genes (Figure 5A, B) suggests that it might influence cancer metabolic reprogramming. This hypothesis is also encouraged by the fact that SLC16A1-AS1 coactivates SLC16A1/MCT1 transcription. To examine if SLC16A1-AS1 affects the metabolic phenotype of tumor cells, we performed Seahorse extracellular flux analysis in high versus low SLC16A1-AS1 expressing bladder cancer cells. Specifically, the most invasive UMUC-3 cells, where the SLC16A1-AS1 gene was effectively CRISPR/Cas9-knocked out were estimated for their bioenergetic profile and metabolic demands versus controls. The data from Seahorse glycolysis stress tests indicated that loss of SLC16A1-AS1 significantly decreased the ECAR-associated with both glycolysis and glycolytic capacity in UMUC-3 cells (Figure 5C). In addition, as determined by Seahorse Mito stress testing, SLC16A1-AS1 depletion caused attenuation of mitochondrial respiration and a markedly reduced adenosine triphosphate (ATP) production (Figure 5D). These experiments show that SLC16A1-AS1 increases aerobic glycolysis in parallel with mitochondria respiration and ATP production.

In order to assess whether SLC16A1-AS1 modifies metabolic phenotypes similarly to SLC16A1/MCT1, we comparatively analysed the energetic phenotypes of high MCT1-expressing UMUC-3 treated with 100 nM AR-C155858 versus DMSO-treated controls. Notably, AR-C155858 treatment deactivates MCT1 without affecting the SLC16A1/MCT1 or SLC16A1-AS1 levels (Figure S9). As expected 69, the MCT1 inhibitor suppressed glycolysis and glycolytic capacity (Figure 5E). In contrast, the mitochondrial function was not drastically affected by pharmacological inhibition (Figure 5F), which is in agreement with recent studies 66,74. Thus, both lncRNA-SLC16A1-AS1 and SLC16A1/MCT1 exert a similar effect on glycolysis, but SLC16A1-AS1 has an additional function with respect to the regulation of mitochondrial respiration and ATP production, not shared by SLC16A1/MCT1 inhibition.

Considering that SLC16A1-AS1 enhances ATP production in mitochondria, we asked if it can also alter the usage of major energy sources that fuel the TCA cycle. Therefore, a Mito fuel flex test was conducted to monitor the dependency of mitochondrial respiration on glucose, glutamine or fatty acids in the presence or absence of SLC16A1-AS1. We found that mitochondrial respiration depends exclusively on fatty acids in UMUC-3 cells (Figure 5G, grey bars). Although there is no dependency on glucose or glutamine, we noticed an increased flexibility for the usage of these substrates in lack of fatty acids. Both the dependency on fatty acids (Figure 5G, grey bars) as well as the flexibility for using other substrates (Figure 5G, black bars) was reduced upon knockout of SLC16A1-AS1. In agreement with the effect on fatty acid β-oxidation (FAO), the mRNA levels of other lipid metabolism markers, such as CD36 and carnitine palmitoyltransferase I (CPT1) 75,76, were found responsive to SLC16A1-AS1 (data not shown). The cell-surface antigen CD36 uptakes lipids from the extracellular environment 76. Following break-down of fatty acids to long-chain acyl-CoAs, the mitochondria membrane-localized CPTI, acting as a rate limiting enzyme of FAO, converts acyl-CoAs into acylcarnitines which can be further processed to acyl-coA and enter the TCA cycle 75.

In conclusion, the lncRNA-SLC16A1-AS1 not only increases aerobic glycolysis in functional relation to its associated PCG, but also enhances mitochondria respiration. This increased oxidative phosphorylation (OXPHOS) is fueled by fatty acids in SLC16A1-AS1-overexpressing bladder cancer cells, while these cells also maintain the ability to support mitochondrial respiration by usage of alternative fuels.

### Characterization of SLC16A1-AS1-induced targets and pathways associated with bladder cancer aggressiveness

**Figure 6:**
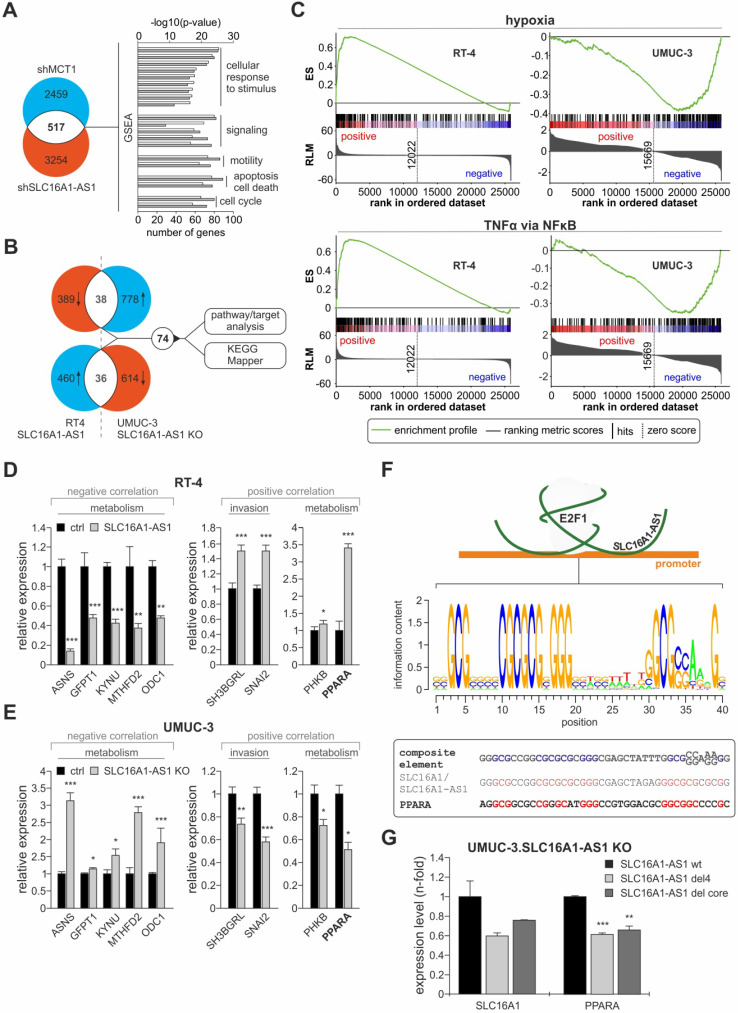
** Identification of targets and pathways associated with SLC16A1-AS1 in invasive bladder cancer.** (**A**) Several differentially expressed genes upon knockout of SLC16A1-AS1 are in common with those altered upon shRNA-mediated knockdown of MCT1, and are enriched for similar functions. (**B**) Venn diagrams of (top) downregulated genes in RT-4.SLC16A1-AS1 cells versus upregulated genes in UMUC-3-KO cells, and (bottom) upregulated genes in RT-4.SLC16A1-AS1 cells versus downregulated genes in UMUC-3-KO cells revealed that 74 genes are commonly affected upon SLC16A1-AS1-mediated invasiveness in bladder cancer cells. Specific/distinct genes of overrepresented pathways were identified using KEGG Mapper. (**C**) GSEA analysis of genes differentially expressed in RT-4.SLC16A1-AS1 or UMUC-3-KO mRNA microarrays show that hypoxia (top) and TNFα signaling via NFκB (bottom) are activated upon SLC16A1-AS1 addition, but suppressed upon SLC16A1-AS1 KO in bladder cancer (**D-E**) qPCR of representatives of the 74 genes that are commonly affected upon SLC16A1-AS1-mediated cancer progression confirmed alterations in key metabolic effectors and mediators of bladder cancer invasiveness. Expression of the same SLC16A1-AS1-negatively correlated genes is enhanced upon SLC16A1-AS1 addition and reduced upon SLC16A1-AS1 knockout (left panels). Vice versa, expression of the same SLC16A1-AS1-positively correlated genes is enhanced upon SLC16A1-AS1 addition and reduced upon SLC16A1-AS1 knockout (right panels, compare D with E) (n=3). (**F**) Top: Scheme of the lncRNA:E2F1 responsive element on the MCT1 promoter. Center: Calculated PWM of the composite binding site. Bottom: Comparison between the composite element of SLC16A1-AS1:E2F1 and the SLC16A1-AS1 and PPARA promoter regions. In the composite element nucleotides essential for lncRNA/E2F1:DNA interaction are highlighted in blue. In the promoters of SLC16A1-AS1 and PPARA nucleotides identical to the composite element are highlighted in red. (**G**) qPCR levels of SLC16A1 and PPARA following transfection of UMUC-3-KO cells with plasmids expressing wild-type SLC16A1-AS1, del4 or del core mutants. Bar graphs are represented as means ± SD.

The E2F1-induced activation of the SLC16A1/SLC16A1-AS1 could lead to changes in downstream genes that mediate their effects on metabolism and tumor aggressiveness. With respect to the functional and mechanistic connection of SLC16A1-AS1 and SLC16A1/MCT1, we wondered whether the putative targets of lncRNA-SLC16A1-AS1 are, to a certain extent, overlapping with the downstream effectors of MCT1, which has a well-established role in cancer invasiveness, in a cancer type-independent manner. This evidence would further support that, by co-activating SLC16A1/MCT1, SLC16A1-AS1 enhances MCT1 expression and augments the glycolytic and invasive phenotypes. To examine this aspect, we subjected UMUC-3 cells ablated for lncRNA and their corresponding control to HTA2.0 transcriptomics analysis and compared the profiles of differentially expressed genes with the expression pattern upon shRNA-mediated knockdown of MCT1 in cancer cells (GSE76675). We found 517 genes that are responsive to both, MCT1 knockdown and SLC16A1-AS1 knockout. Further Gene Set Enrichment Analysis (GSEA) indicated that these genes are involved in cellular response to endogenous stimuli and to lipids, metabolism and cell migration, supporting a role of the SLC16A1/SLC16A1-AS1 gene pair in the coordinated regulation of these processes (Figure 6A, Table S7).

Additionally, based on the 'guilt-by-association' analysis, SLC16A1-AS1 is implicated in divergent processes (Figure 5A), leading to the assumption that it may not merely act on SLC16A1/MCT1 induction and Warburg effect, but also exert pleiotropic effects on additional pathways either through the identified or a distinct mechanism. To identify gene targets by which SLC16A1-AS1 mediates its functions, we postulated that the same targets/pathways that are upregulated when non-invasive RT-4 cells are transferred into an invasive state upon SLC16A1-AS1 addition will be downregulated when invasive UMUC-3 cells acquire reduced invasiveness due to SLC16A1-AS1 depletion. Likewise, targets/pathways that are downregulated during non-invasive to invasive transition in response to SLC16A1-AS1 expression should be upregulated in lncRNA-knockout cells. Analyses of differentially expressed genes revealed 74 genes that are commonly altered (2-fold) in RT-4.SLC16A1-AS1 and UMUC-3.SLC16A1-AS1 KO clones versus the parental controls, which are mainly in-trans relative to SLC16A1-AS1 (Figure 6B, upper panel; Table S8). GSEA analysis of the array data demonstrated that hypoxia and TNFα-via-NFκB gene sets are upregulated in lncRNA-depleted UMUC-3 cells, but downregulated in RT-4.SLC16A1-AS1 (Figure 6C). Additionally, using KEGG mapper 77, we found that several SLC16A1-AS1 targets are involved in pathways not only related with metabolism (amino acid metabolism, glutamate metabolism, glucagon signaling and insulin resistance), but also with innate immune system response (NOD-like signaling), (Table S9). Verification of representative target genes by qPCR showed that metabolic effectors ASNS, GFPT1, KYNU, MTHFD2, and ODC1 are downregulated in RT-4 with high SLC16A1-AS1 and upregulated in UMUC-3-KO cells. Moreover, SLC16A1-AS1 enhances the glucagon signaling pathway effectors PPARA and PHKB, as well as genes that have been shown to mediate bladder cancer invasiveness, such as SNAI2 78 and SH3BGRL 79 (Figure 6D, E).

Among the validated targets, peroxisome proliferator-activated receptor alpha (PPARA), which is a key mediator of fatty acid oxidation 80, emerged as an attractive candidate. Since SLC16A1-AS1-induced overexpression can be correlated with the enhanced FAO phenotype in UMUC-3 compared to the KO cells (Figure 5G), we examined whether PPARA is also regulated through a composite E2F1/lncRNA responsive element (Figure 4H). First, we searched for the presence of this element across promoters of genes that are deregulated upon either SLC16A1-AS1 addition in RT-4 or UMUC-3-KO cells (Figure 6B). In detail, a position weight matrix (PWM) was composed of the SLC16A1-AS1 binding sequence and E2F1 consensus PWM shown in Figure 3C, which derived from all three described PWMs (MA.00024.1, -2, and -3, Table S10). We confirmed that the validated E2F1 binding site on the MCT1 promoter shows high homology to this consensus motif. Next, we screened for the existence of the calculated composite E2F1:SLC16A1-AS1 binding site on SLC16A1-AS1-responsive genes obtained from our array analyses (Figure 6B) using the Galaxy server (bedtools: closestBed) 36 and enhanced the stringency of the analysis by filtering the results with ChIP-seq validated E2F1 binding sites based on GTRD database. We found that the PPARA promoter harbors a highly similar E2F1:SLC16A1-AS1 motif (Figure 6F), implying that PPARA transcription may also be susceptible to the E2F1:SLC16A1-AS1 interaction. Indeed, addition of SLC16A1-AS1 mutants harboring deletions in the identified E2F1 binding regions in UMUC-3-KO cells led to reduced transcription of both PPARA and SLC16A1, as compared to addition of the wild-type SLC16A1-AS1 (Figure 6G). Overall, the fact that the composite element and the transcriptional response of PPARA is highly similar to the validated gene target MCT1 of the E2F1:lncRNA complex, clearly highlights that it can be induced by the E2F1:SLC16A1-AS1 interaction.

Overall, SLC16A1-AS1-induced cancer progression is not mediated merely by SLC16A1 upregulation, but also orchestrated via systemic changes in additional targets and pathways, of which PPARA is a SLC16A1-AS1 responsive gene that can follow a mechanism of transactivation similar to the one identified for SLC16A1.

## Discussion

In this study, we demonstrated that in conjunction with PCGs, E2F1 induces expression of lncRNAs in bladder cancer, which are relevant for disease progression. We show that E2F1 transactivates the aerobic glycolysis regulator SLC16A1/MCT1 in parallel with its antisense lncRNA-SLC16A1-AS1 via a shared E2F1-responsive promoter, an event that is particularly associated with tumor progression to muscle-invasive stages and poor patient outcomes. As predicted by our computational simulations and experimentally validated, the newly-synthesized, nuclear-localized SLC16A1-AS1, in turn, creates a lncRNA-protein complex with E2F1 which facilitates E2F1 binding to the SLC16A1/MCT1 promoter. We identified a composite E2F1/lncRNA regulatory element on the common promoter, to which the SLC16A1-AS1:E2F1 complex is able to bind and co-activate SLC16A1/MCT1, thus creating a coherent feedforward loop which ultimately leads to enhanced aerobic glycolysis. In addition, SLC16A1-AS1 upregulates oxidative phosphorylation in mitochondria. This is associated with SLC16A1-AS1-induced FAO and upregulation of its key regulator PPARA, also bearing the E2F1/lncRNA motif. The SLC16A1-AS1-expressing BC cells are more dependent on fatty acids for their mitochondria function, while they present enhanced flexibility for other nutrients, such as glucose and glutamine, in case of fatty acids deprivation. As a result, transactivation of the SLC16A1/SLC16A1-AS1 gene pair establishes a hybrid glycolysis/OXPHOS metabolic phenotype, which is accompanied by increased cell invasiveness.

It is becoming more and more evident that lncRNAs adopt higher order tertiary structures which are important for their mechanisms of action and functions 81. However, unveiling structural aspects of lncRNAs is a particularly challenging task, since the poor conservation of their primary sequence in combination with their variable and long lengths put obstacles in the prediction of their secondary and tertiary structures using existing models 82,83. Herein, we established a computational model focusing on predicting and simulating structure-based RNA-protein and RNA-protein-DNA interactions, followed by robust experimental validation. The generated workflow not only prepares the secondary and tertiary structure of the full-length SLC16A1-AS1, but also reliably predicts the interacting surfaces between E2F1 and SLC16A1-AS1, as well as the distinct DNA binding sites of the lncRNA:protein complex on target gene promoters. To our knowledge, this is the first computational pipeline to understand the mechanistic insights of lncRNA in stabilizing transcription factor on the promoter of downstream genes at the structural level. Notably, this approach also enabled us to demonstrate a case where the lncRNA:protein interaction depends more on the structure of the SLC16A1-AS1 and less on its primary sequence, recapitulating the emerging notion that, for lncRNAs, structural conservation rather than nucleotide sequence conservation seems to be crucial for maintaining their function 81. In this regard, our model may hold promise to aid characterization of structural domains of the RNA interactome and shed light on the enigmatic link between lncRNA structure and function.

The Warburg effect has been regarded as the dominant metabolic phenotype in cancer cells for almost one century, rapidly supplying energy and biosynthetic intermediates for quick proliferation. It was originally thought that this preference for glycolysis was due to defects in the mitochondria of cancer cells. However, advances in understanding cancer metabolism have weakened this hypothesis, counter suggesting that mitochondria are not only functional in tumor cells, but also orchestrate aggressive and metastatic phenotypes 84-86. The mitochondrial function can be retained in malignant cells through oxidation of alternative nutrients, such as fatty acids and glutamine, which enter the TCA cycle via conversion to citrate and α-ketoglutarate. This way, cancer cells ensure the production of ATP, TCA intermediates and building blocks (amino acids and pentoses) to meet their energy and biosynthetic demands 87-89. Mitochondrial respiration is a metabolic trait that characterizes dormant or circulating cancer cells 90,91, and also facilitates clonogenic survival and resistance to radiation treatment 92. Hence, instead of an exclusively glycolytic phenotype, invasive/metastatic cancer cells may exhibit a hybrid metabolic phenotype, showing both glycolysis and oxidative phosphorylation 91,93.

The hybrid glycolysis / OXPHOS metabolic state is particularly advantageous for aggressive tumors. It offers (a) increased metabolic flexibility, since cancer cells are able to utilize various kinds of available nutrients in response to fluctuating microenvironmental conditions, (b) redundancy in the production of energy and anabolic intermediates, (c) maintenance of reactive oxygen species (ROS) at a moderate level, a fact that protects them from detrimental effects of excessive ROS production and promotes mutagenic events that stimulate tumorigenesis and metastasis, (d) increased capability for EMT-dependent metastasis, which is accompanied by enhanced glycolytic and OXPHOS activities, and (e) resistance to anticancer therapy 91. In light of these findings, our data strongly suggest that, via supporting the acquisition of a hybrid glycolysis/OXPHOS phenotype, E2F1-triggered activation of the SLC16A1/SLC16A1-AS1 gene pair promotes bladder cancer progression by mediating metabolic plasticity.

We also found that expression of SLC16A1-AS1 in aggressive BC cells increases their dependency of mitochondrial function on FAO. Consistently, PPARA was identified as lncRNA trans-regulated target that contains responsive elements for the SLC16A1-AS1:E2F1 complex and is upregulated during BC progression to invasive stages. Our results are in line with current studies proposing a tumor-promoting role for mitochondrial fatty acid β-oxidation. In particular, abnormal FAO activity has been implicated in cancer initiation and progression, while tumor cells rely on this major catabolic process in cell fate control, such as proliferation, survival, stemness, metastatic spread or drug resistance 94,95. Metabolomics analyses in clinical samples from BC patients demonstrated that perturbations of FAO go along with alterations in glycolysis, TCA cycle and amino acid metabolism 96.

Besides, as predicted by our target/molecular pathway simulations, SLC16A1-AS1 influences additional cellular processes, for instance hypoxia pathways and the NFκB-mediated response to pro-inflammatory TNFα, and was shown to affect expression of additional metabolic and invasive targets. A hypothetical scenario to comprehensively explain the observed alterations on the aforementioned signaling components is that SLC16A1-AS1 lncRNA may have broader, systemic functions at the crossroads among metabolism, hypoxia and inflammation. Based on this, it would also be plausible to suspect that SLC16A1-AS1 is capable of controlling the interplay between immunological and metabolic processes to shape cancer immunometabolism. However, these possible functional connections remain to be investigated. High-throughput RNA-centric approaches to dissect SLC16A1-AS1-associated proteins and chromatin DNA 97 together with, for example, 3D chromatin structures from chromosome conformation capture techniques 98 could identify protein partners and/or mechanisms through which this lncRNA contributes to gene regulation.

Our data underscore that, beyond its well-known role in controlling cell cycle, E2F1 reprograms cancer metabolism to support metastatic characteristics. In addition to orchestrating the Warburg effect via transactivating several glycolysis-related targets (reviewed in 99), it also increases metabolic flexibility of invasive versus non-invasive cancer cells via physical association with a lncRNA. More than merely sustaining the Warburg phenotype, the establishment of a coherent feedforward loop within the SLC16A1-AS1:E2F1/SLC16A1/MCT1 regulatory axis rewires E2F1 with lipid and mitochondrial metabolism, i.e. two metabolic traits that are emerging as essential factors of tumor progression 100. These hints also highlight that, mechanistically, there are metabolic differences between early and late cancer stages, which should be considered when designing personalized anti-metastatic therapies. The functions of E2F1 on normal or early tumor versus advanced cancer stages are dynamic and context-specific. For instance, E2F1 suppresses OXPHOS in normal tissues 101, whereas it reactivates this process via SLC16A1-AS1 in advanced-stage tumors. The feedforward loop which is identified herein as a component of the E2F1-governed prometastatic GRN enables us to extrapolate that a mere inhibition of glycolysis may be suboptimal to eradicate an invasive tumor, since these cells could still be able to survive through producing energy and building blocks via fatty acid-fuelled OXPHOS.

From the cancer therapeutics' point of view, our findings provide new insights on potential mechanisms underlying acquired resistance of cancer cells to MCT1 inhibitors. In particular, blockade of lactate transport by inhibition of MCT1 is used as attractive treatment strategy 63,102. MCT1 inhibition causes intracellular accumulation of lactate, decrease of intracellular pH, suppression of glycolysis and tumor shrinkage, while it negatively affects tumor cells that depend on lactate import to fuel OXPHOS under conditions of limited glucose availability 102. Although several MCT1 inhibitors lead to a decrease of tumor growth *in vivo* and have entered phase I/II clinical trials 68, they fail to achieve complete regression 102. Resistance to MCT1 inhibitors is acquired via activation of mitochondrial respiration 68. Our results indicate that MCT1 is inevitably co-transcribed and -expressed with the mitochondrial respiration activator SLC16A1-AS1 in a high-E2F1 cancer cell context. SLC16A1-AS1 lncRNA is still abundant even after MCT1 deactivation, thereby theoretically offering cancer cells a selective advantage for evading the tumor suppressing effect of MCT1 inhibitors through enhancement of respiration. Future studies will demonstrate if and how SLC16A1-AS1 is implicated in cases of acquired resistance to pharmacological MCT1 inhibition. If SLC16A1-AS1 indeed favors resistance to MCT1 inhibitors via OXPPHOS, concomitant transactivation of the SLC16A1/SLC16A1-AS1 pair imparts an ab initio resistance to these specific drugs. Even when a SLC16A1/MCT1-expressing tumor is initially responsive to these drugs, the persistent co-upregulation of SLC16A1-AS1 will provide it a means to become refractory through alternative metabolic pathways. Such a scenario implicates that MCT1 inhibition cannot ensure durable therapeutic responses, unless routinely combined with inhibitors of mitochondrial respiration.

It is becoming increasingly evident that lncRNAs fine-tune cancer metabolic reprogramming, and several have been proposed as appealing therapeutic targets 103-105. However, translation of these findings into therapeutic solutions should be considered with caution. This is mainly because heterogeneity of cancer cells is inconsistent with the existence of a universal cancer cell metabolic map 89. When considering metabolism-targeting therapies, one should essentially keep in mind that a tumor might consist of heterogeneous cell populations with varying metabolic profiles. Moreover, the metabolic phenotype is not necessarily uniform across different tumors or even within tumors of the same type 91. The landscape gets even more complicated by realizing that metastasis-initiating cells differ from their non-metastatic counterparts in terms of metabolic plasticity 100. These differences inevitably reflect to a high diversity in metabolic traits of tumors across patient populations, posing limitations on these therapeutic approaches. For example, FAO has been shown to orchestrate (95,96 and this study) also inhibit 106 malignant progression in different bladder cancer patient cohorts. In this regard, metabolic heterogeneity rather creates transient and location-dependent phenotypes, which may not constitute suitable targets, unless largely predominant 89. Furthermore, due to the emerging links between nutrition and cancer risk 107, it is intriguing to postulate that cancer metabolic reprogramming is further shaped by dietary habits which largely vary within a population, although this plausible hypothesis requires future rigorous investigation. Such intra- and intertumoral metabolic complexity combined with an incomplete characterization of the lncRNA landscape pose challenges on the fast-track development of lncRNA-based therapeutics to manage cancer metabolism 104. A deeper understanding of the role of lncRNAs in cancer metabolism, especially in relation with parameters such as tumor heterogeneity and intrapopulation variation in the diet, is mandatory for their value as monitoring tools and therapy targets, in order to improve personalized precision cancer medicine.

## Conclusion

In our study we identified a new gene regulatory program initiated by the E2F1-mediated expression of lncRNA-SLC16A1-AS1 in aggressive BC. We showed that SLC16A1-AS1 forms an RNA-protein complex with its own transcription factor E2F1 to promote cancer metabolic reprograming and an invasive phenotype. Importantly, the complex alters the expression of the target genes SLC16A1/MCT1 and PPARA that contribute to BC malignancy, through binding to a composite SLC16A1-AS1:E2F1-responsive element. Based on these findings, we provide new functional and mechanistic insights on the effect of E2F1-regulated lncRNAs in cancer metabolism.

## Supplementary Material

Supplementary figures and tables.

## Abbreviations

4-OHT: 4-hydroxytamoxifen
ECAR: extracellular acidification rate
EMT: epithelial-mesenchymal transition
FAO: fatty acid β-oxidation
GRN: gene regulatory network
GSEA: gene set enrichment analysis
MIBC: muscle invasive bladder cancer
NMIBC: non-muscle invasive bladder cancer
OCR: oxygen consumption rate
OXPHOS: oxidative phosphorylation
PCGs: protein coding genes
PWM: position weight matrix
