# Supplementary material for: LncRNA-SLC16A1-AS1 induces metabolic reprogramming during Bladder Cancer progression as target and co-activator of E2F1
Source: Theranostics. 2020 Jul 29;10(21):9620–43. doi: 10.7150/thno.44176 (PMC7449907; doi:10.7150/thno.44176)
Supplement: Supplementary file 1 — Supplementary figures and tables. [file thnov10p9620s1.pdf]

Supplementary Material

LncRNA-SLC16A1-AS1 induces metabolic reprogramming during bladder cancer progression as target and co-activator of E2F1

Stella Logotheti[1,†], Stephan Marquardt[1,†], Shailendra K. Gupta[3,†], Christin Richter[1,†], Berdien A.H. Edelhäuser[1], David Engelmann[1], Julia Brenmoehl[4], Christoph Söhnchen[1], Nico Murr[1], Michael Alpers[1], Krishna P. Singh[3], Olaf Wolkenhauer[3], Dirk Heckl[5], Alf Spitschak[1], and Brigitte M. Pützer[1,2,*]

[1]Institute of Experimental Gene Therapy and Cancer Research, Rostock University Medical Center, 18057 Rostock, Germany

[2]Department Life, Light & Matter, University of Rostock, 18059 Rostock, Germany

[3]Department of Systems Biology and Bioinformatics, University of Rostock, 18057 Rostock, Germany

[4]Leibniz Institute for Farm Animal Biology (FBN), Institute of Genome Biology, Signal Transduction Unit, 18196 Dummerstorf, Germany

[5]Pediatric Hematology and Oncology, Hannover Medical School, 30625 Hannover, Germany

*Corresponding author. Tel: +49 381 494 5066/68; Fax: +49 381 494 5062; Email: brigitte.puetzer@med.uni-rostock.de

[†]These authors contributed equally to this work.

Tables

Table S1: Primers used for PCR and mutagenesis

primer name	sequence (5´→3´)
**Actin_FWD**	CGGGAAATCGTGCGTGACATTA
**Actin_RVS**	ACCGCTCATTGCCAATGGTGAT
**ASNS_FWD**	CGACCAAAAGAAGCCTTCAG
**ASNS_RVS**	CCACTTGGGCATCCAGTAAT
**GAPDH_FWD**	ATCGTGGAAGGACTCATGACCACA
**GAPDH_RVS**	AAGGCCATGCCAGTGAGCTTC
**GFPT1_FWD**	CAAAGGCCTTCAGAGACTGG
**GFPT1_RVS**	AGGACTGGGTTCTCCATGTG
**E2F1_FWD**	GCTGGACCACCTGATGAA
**E2F1_REV**	GGAGGGGCTTTGATCACC
**KYNU_FWD**	CAAGAGAGGGGGAAGAAACC
**KYNU_RVS**	TAACAACCCTTCGCTTGTCC
**MTHFD2_FWD**	TTGACCTCCTGACCTCTGCT
**MTHFD2_RVS**	TTCCAAGGGATACTGCCAAG
**ODC1_FWD**	CAAAGTTGGTTTTGCGGATT
**ODC1_RVS**	CGAAGGTCTCAGGATCGGTA
**PHKB_FWD**	ACGTGAGCATGAGGTTTTCC
**PHKB_RVS**	TGTCTGGCCTTCCAGAGAGT
**PPARA_RVS**	GATCTTGGCATTCGTCCAA
**PPARA-FWD**	GAGCATTGAACATCGAATGTAG
**SH3BGRL_FWD**	TTGCATCTTCCTCTGGCTCT
**SH3BGRL_RVS**	CTGGCTTCAAAGAAGGCATC
**SLC16A1_FWD**	TCAGGCTGTGGCTTGATTGC
**SLC16A1_RVS**	GCCAATGGTCGCCTCTTGTAGAA
**SLC16A1-AS1 KO1_FWD**	AGAGGTCCCATGTGCTTTCCAA
**SLC16A1-AS1 KO1_RVS**	CAATGACCCGAAATGCCTTCCCTT
**SLC16A1-AS1 KO2_FWD**	AGCTACCACGCCCTGCCAGACT
**SLC16A1-AS1 KO2_RVS**	TTGCCCAGGCTGGAGTGCAATGAT
**SLC16A1-AS1_FWD**	GGGAGACTTAGGCACAAATTAACC
**SLC16A1-AS1_RVS**	ATGTTGGTGTGCTTGAAATCTTCC
**SNAI2_FWD**	ATGAGGAATCTGGCTGCTGT
**SNAI2_RVS**	TGAATTCCATGCTCTTGCAG
**MCT1 mut-Prom FWD**	ACATCCGAACTGCCCTCCCCGCCACG
**MCT1 mut-Prom REV**	CGTGGCGGGGAGGGCAGTTCGGATGT
**SLC16A1-AS1_del1-FWD**	CCGAGAGGCCTGACCCAGCATTTGAATGGATG
**SLC16A1-AS1_del1-REV**	CATCCATTCAAATGCTGGGTCAGGCCTCTCGG
**SLC16A1-AS1_del2-FWD**	CGGGGAATGGGCAGCATTTCATGGGCTGGG
**SLC16A1-AS1_del2-REV**	CCCAGCCCATGAAATGCTGCCCATTCCCCG
**SLC16A1-AS1_del4-FWD**	GGGGGAGATGCGCGCGACTGAGGAGC
**SLC16A1-AS1_del4-REV**	GCTCCTCAGTCGCGCGCATCTCCCCC
**SLC16A1-AS1_del core-FWD**	GATGCGCGTGCGCGGGAAGCGACT
**SLC16A1-AS1_del core-REV**	AGTCGCTTCCCGCGCACGCGCATC

**U6-FWD**	CTCGCTTCGGCAGCACATATAC
**U6-REV**	AACGCTTCACGAATTTGCGTGTC

Table S2: GSEA of genes from TANRIC atlas positively (n = 1245) and negatively (n = 991) correlated (|k| ≥ 0.4) with SLC16A1-AS1 expression in TCGA-BC.

**TANRIC - positively correlated**						
**Gene Set Name**	**# Genes in Gene Set (K)**	**# Genes in Overlap (k)**	**k/K**	**p-value**	**FDR q-value**	**$-log_{10}$ (p-value)**
GO_CELL_CYCLE	1847	217	0.1175	3.56E-63	2.64E-59	62.45
GO_MITOTIC_CELL_CYCLE	1009	155	0.1536	5.37E-60	1.99E-56	59.27
GO_CELL_CYCLE_PROCESS	1383	181	0.1309	2.44E-59	6.01E-56	58.61
HALLMARK_E2F_TARGETS	200	65	0.325	1.79E-46	3.32E-43	45.75
GO_PROTEIN_PHOSPHORYLATION	1967	185	0.0941	1.5E-39	2.22E-36	38.82
GO_REGULATION_OF_RESPONSE_TO_STRESS	1497	154	0.1029	2.75E-37	3.4E-34	36.56
GO_CELL_CYCLE_PHASE_TRANSITION	621	95	0.153	1.29E-36	1.36E-33	35.89
GO_RESPONSE_TO_CYTOKINE	1192	134	0.1124	1.95E-36	1.8E-33	35.71
GO_REGULATION_OF_CELL_CYCLE	1212	135	0.1114	2.78E-36	1.97E-33	35.56
HALLMARK_G2M_CHECKPOINT	200	56	0.28	2.89E-36	1.97E-33	35.54
GO_REGULATION_OF_IMMUNE_SYSTEM_PROCESS	1631	160	0.0981	2.94E-36	1.97E-33	35.53
GO_CYTOSKELETON_ORGANIZATION	1298	140	0.1079	4.3E-36	2.65E-33	35.37
GO_DEFENSE_RESPONSE	1709	164	0.096	5.19E-36	2.96E-33	35.28
GO_APOPTOTIC_PROCESS	1980	176	0.0889	1.94E-34	1.03E-31	33.71
GO_DNA_METABOLIC_PROCESS	850	107	0.1259	2.08E-33	1.02E-30	32.68
GO_IMMUNE_EFFECTOR_PROCESS	1253	130	0.1038	8.01E-32	3.7E-29	31.10
GO_CHROMOSOME_ORGANIZATION	1207	127	0.1052	1.09E-31	4.76E-29	30.96
GO_REGULATION_OF_PROTEIN_MODIFICATION_PROCESS	1843	162	0.0879	4.34E-31	1.78E-28	30.36
GO_POSITIVE_REGULATION_OF_IMMUNE_SYSTEM_PROCESS	1166	123	0.1055	8.38E-31	3.26E-28	30.08
GO_REGULATION_OF_CELL_DIFFERENTIATION	1863	162	0.087	1.49E-30	5.51E-28	29.83

TANRIC- negatively correlated						
**Gene Set Name**	**# Genes in Gene Set (K)**	**# Genes in Overlap (k)**	**k/K**	**p-value**	**FDR q-value**	**$-\log_{10}$ (p-value)**
GO_LIPID_METABOLIC_PROCESS	1241	139	0.112	4.59E-49	3.39E-45	48.34
GO_CELLULAR_LIPID_METABOLIC_PROCESS	885	113	0.1277	2.31E-45	8.55E-42	44.64
GO_SMALL_MOLECULE_METABOLIC_PROCESS	1688	146	0.0865	3.5E-38	8.63E-35	37.46
GO_ORGANIC_ACID_METABOLIC_PROCESS	903	94	0.1041	1.26E-30	2.33E-27	29.90
GO_MONOCARBOXYLIC_ACID_METABOLIC_PROCESS	475	65	0.1368	4.38E-28	6.48E-25	27.36
GO_LIPID_CATABOLIC_PROCESS	325	51	0.1569	4.71E-25	5.81E-22	24.33
GO_FATTY_ACID_METABOLIC_PROCESS	257	45	0.1751	2.82E-24	2.98E-21	23.55
GO_LIPID_BIOSYNTHETIC_PROCESS	669	71	0.1061	8.05E-24	7.45E-21	23.09
GO_CELLULAR_LIPID_CATABOLIC_PROCESS	207	40	0.1932	2.2E-23	1.81E-20	22.66
GO_OXIDATION_REDUCTION_PROCESS	970	86	0.0887	2.82E-23	2.09E-20	22.55
GO_ORGANOPHOSPHATE_METABOLIC_PROCESS	532	61	0.1147	2.47E-22	1.66E-19	21.61
GO_ORGANIC_ACID_CATABOLIC_PROCESS	250	42	0.168	5.01E-22	3.09E-19	21.30
GO_SMALL_MOLECULE_CATABOLIC_PROCESS	396	50	0.1263	2.5E-20	1.42E-17	19.60
HALLMARK_ESTROGEN_RESPONSE_EARLY	200	35	0.175	3.59E-19	1.90E-16	18.44
GO_INTRACELLULAR_TRANSPORT	1825	111	0.0608	5.94E-17	2.93E-14	16.23
GO_MONOCARBOXYLIC_ACID_CATABOLIC_PROCESS	122	26	0.2131	8.81E-17	4.07E-14	16.06
GO_FATTY_ACID_CATABOLIC_PROCESS	103	24	0.233	1.52E-16	6.63E-14	15.82
GO_CARBOHYDRATE_DERIVATIVE_METABOLIC_PROCESS	940	72	0.0766	3.21E-16	1.32E-13	15.49
GO_LIPID_MODIFICATION	235	34	0.1447	4.80E-16	1.87E-13	15.32
GO_LIPID_OXIDATION	101	23	0.2277	1.12E-15	4.14E-13	14.95

Table S3 (related to Figure 4B): Top 20 potential interaction sites between E2F1 and SLC16A1-AS1 predicted by catRAPID. Discriminative Power (DP) ranges from 0% (unpredictability) to 100% (predictability). DP values above 50% indicate that the interaction is likely to take place, whereas DPs above 75% represent high-confidence predictions.

E2F1 region (amino acides)	SLC16A1-AS1 region (base pairs)	discriminative power (%)
**312-363**	241-302	63
**51-102**	241-302	59
**87-138**	241-302	59
**162-213**	241-302	59
**276-327**	241-302	59
**312-363**	262-323	57
**87-138**	262-323	54
**212-263**	241-302	54
**251-302**	241-302	54
**301-352**	241-302	54
**51-102**	262-323	52
**276-327**	262-323	52
**51-102**	232-293	50
**162-213**	232-293	50
**162-213**	262-323	50
**201-252**	241-302	50
**276-327**	232-293	50
**312-363**	151-212	50
**312-363**	172-233	50
**312-363**	211-272	50

Table S4 (related to Figure 4B): Secondary structure level analysis of top 20 potential interaction sites between E2F1 and SLC16A1-AS1. Surface accessibility is calculated using NetSurfP-2.0. RSA, Relative Surface Accessibility; ASA, Absolute Surface Accessibility. RSA reflects the degree to which a residue interacts with the solvent molecule. Accessible surface area or Absolute Surface Accessibility is the surface area of a biomolecule that is accessible to a solvent. RSA=ASA/max ASA. The asterisk marks the best combination of the E2F1:SLC16A1-AS1 complex based on the secondary structure parameters.

complex binding site (no.)	protein region (amino acids)	average RSA	average ASA	lncRNA region (base pairs)	number of stem forming bases (SLC16-A1-AS1)	number of loop forming bases (SLC16-A1-AS1)
**1**	312-363	0.623423077	92.69209615	241-302 (62)	45 (72.6%)	17 (27.4%)
**2**	51-102	0.574923077	86.88392308	241-302 (62)	45 (72.6%)	17 (27.4%)
**3**	87-138	0.539538462	86.16046154	241-302 (62)	45 (72.6%)	17 (27.4%)
**4**	162-213	0.372019231	60.44236538	241-302 (62)	45 (72.6%)	17 (27.4%)
**5**	276-327	0.484346154	72.46588462	241-302 (62)	45 (72.6%)	17 (27.4%)
**6**	312-363	0.623423077	92.69209615	262-323 (62)	48 (77.4%)	14 (22.6%)
**7**	87-138	0.539538462	86.16046154	262-323 (62)	48 (77.4%)	14 (22.6%)
**8**	212-263	0.377961538	62.41503846	241-302 (62)	45 (72.6%)	17 (27.4%)
**9**	251-302	0.299269231	47.28023077	241-302 (62)	45 (72.6%)	17 (27.4%)
**10**	301-352	0.634346154	90.76119231	241-302 (62)	45 (72.6%)	17 (27.4%)
**11**	51-102	0.574923077	86.88392308	262-323 (62)	48 (77.4%)	14 (22.6%)
**12**	276-327	0.484346154	72.46588462	262-323 (62)	48 (77.4%)	14 (22.6%)
**13**	51-102	0.574923077	86.88392308	232-293 (62)	47 (75.8%)	15 (24.2%)
**14**	162-213	0.372019231	60.44236538	232-293 (62)	47 (75.8%)	15 (24.2%)
**15**	162-213	0.372019231	60.44236538	262-323 (62)	48 (77.4%)	14 (22.6%)
**16**	201-252	0.407384615	68.73494231	241-302 (62)	45 (72.6%)	17 (27.4%)
**17**	276-327	0.484346154	72.46588462	232-293 (62)	47 (75.8%)	15 (24.2%)
**18**	312-363	0.623423077	92.69209615	151-212 (62)	39 (62.9%)	23 (28.1%)
**19***	312-363	0.623423077	92.69209615	172-233 (62)	36 (58.1%)	26 (41.9%)
**20**	312-363	0.623423077	92.69209615	211-272 (62)	48 (77.4%)	14 (22.6%)

Table S5 (related to Figure 4F and G): Overview of the minimum free energies (MFE) of the mutants of SLC16A1-AS1 and binding energies of the corresponding complex structures with E2F1.

In silico mutagenesis	MFE (Kcal/mol) of the lncRNA (RNAfold)	Binding energy (Kcal/mol) of the E2F1:lncRNA complex (FireDock)
**wild-type**	-525.6	-35.68
del1	-492.3	-43.67
del2	-480.7	-28.51
del3	-449.7	-49.61
del4	-518.5	-33.47
del5	-524.8	-57.29
del6	-517.3	-24.66

Table S6 (related to Figure 4H): Sequence based identification of lncRNA-DNA triplex formation sites on the MCT1 promoter using the LongTarget tool (http://lncrna.smu.edu.cn/show/DNATriplex)

	QueryStart (SLC16A1-AS1)	QueryEnd (SLC16A1-AS1)	StartInSeq (MCT1 promoter)	EndInSeq (MCT1 promoter)	Direction	Mean Stability	Mean Identidy (%)	Strand	Rule	Score	base pairs	Class	Mid Point	Center
**1**	1077	1130	-262	-315	L	1.49	61.11	AntiPlus	17	1.5	54	1	1103	1106
**2**	1059	1125	-253	-315	L	1.71	61.19	AntiPlus	14	1.15	67	1	1092	1106
**3**	1065	1129	-315	-253	R	2.73	60.61	ParaPlus	2	1.09	66	1	1097	1106
**4**	1075	1133	-252	-313	L	1.83	65.08	AntiPlus	14	1.29	63	1	1104	1106
**5**	1079	1142	-252	-315	L	1.91	63.08	AntiPlus	14	1.31	65	1	1110	1106
**6**	1091	1142	-255	-307	L	1.88	66.04	AntiPlus	14	1.72	53	1	1116	1106
**7**	1064	1130	-314	-252	R	2.67	61.19	ParaPlus	5	1.15	67	1	1097	1106
**8**	1080	1130	-314	-262	R	2.74	64.15	ParaPlus	5	1.55	53	1	1105	1106
**9**	1091	1142	-313	-265	R	2.8	61.54	ParaPlus	2	1.31	52	1	1116	1106
**10**	1079	1142	-252	-315	L	1.9	62.69	AntiPlus	8	0.93	67	1	1110	1106
**11**	1078	1129	-265	-315	L	1.95	63.46	AntiPlus	5	1.48	52	1	1103	1106
**12**	1077	1133	-255	-311	L	1.84	61.4	AntiPlus	5	1.53	57	1	1105	1106
**13**	1073	1132	-312	-252	R	2.58	62.3	ParaPlus	2	1.41	61	1	1102	1106
**14**	1075	1142	-252	-315	L	1.88	62.32	AntiPlus	2	1.09	69	1	1108	1106
**15**	1054	1115	-260	-315	L	1.81	66.67	AntiPlus	14	1.24	63	2	1084	1089
**16**	1052	1115	-251	-315	L	1.99	61.54	AntiPlus	2	1.35	65	2	1083	1089
**17**	1059	1120	-313	-252	R	2.57	60.0	ParaPlus	2	1.03	65	2	1089	1089
**18**	1059	1110	-303	-252	R	2.87	64.15	ParaPlus	2	1.32	53	2	1084	1089
**19**	1055	1113	-259	-315	L	1.87	60.0	AntiPlus	2	1	60	2	1084	1089
**20**	1470	1521	-253	-302	L	1.1	63.46	AntiPlus	9	1.48	52	3	1495	1495
**21**	617	679	-311	-251	R	1.85	60.32	ParaPlus	4	1.05	63	4	648	648
**22**	575	625	-262	-312	L	1.77	63.46	AntiPlus	10	1.25	52	5	600	600
**23**	251	313	-315	-253	R	1.96	60.0	ParaPlus	4	0.85	65	6	282	282
**24**	157	207	-268	-315	L	1.77	62.75	AntiPlus	10	1.18	51	7	182	182

Table S6 (related to Figure 4H): continued

	TFO sequence	TTS sequence
1	CCUCCCUUCCAGGACUCACCCCCUCACUUACCCUCCCCCUCACUUACCCUCCCC	TCCACACGCTTTCAGCCGCGCGCGCCCTCTAGCTCGCCCGCGCGCGCCGGCGCC
2	CCCUUCCCCUCGUGUCUUCCUCCCUUCCAGGACUCACCCCCUCACUUACCCUCCCCCUCACUUACCC	GCCCGCGCCTCCACACGCTTTCAGCCGCGCGCGCCCTCTAGCTCGCCCGCGCGCGCCGGCGCC
3	CCCUCGUGUCUUCCUCCCUUCCAGGACUCACCCCCUCACUUACCCUCCCCCUCACUUACCCUCCC	GGCGCCGGCGCGCGCGGGCGAGCTAGAGGGCGCGCGCGGCTGAAAGCGTGTGGAGGCGCGGGC
4	UUCCUCCCUUCCAGGACUCACCCCCUCACUUACCCUCCCCCUCACUUACCCUCCCCCUC	AGCCCGCGCCTCCACACGCTTTCAGCCGCGCGCGCCCTCTAGCTCGCCCGCGCGCGCCGGCG
5	UCCCUUCCAGGACUCACCCCCUCACUUACCCUCCCCCUCACUUACCCUCCCCCUCAAUUCUCAC	AGCCCGCGCCTCCACACGCTTTCAGCCGCGCGCGCCCTCTAGCTCGCCCGCGCGCGCCGGCGCC
6	CUCACCCCCUCACUUACCCUCCCCCUCACUUACCCUCCCCCUCAAUUCUCAC	CCGCGCCTCCACACGCTTTCAGCCGCGCGCGCCCTCTAGCTCGCCCGCGCGCG
7	CCCCUCGUGUCUUCCUCCCUUCCAGGACUCACCCCCUCACUUACCCUCCCCCUCACUUACCCUCCCC	GCGCCGGCGCGCGCGGGCGAGCTAGAGGGCGCGCGCGGCTGAAAGCGTGTGGAGGCGCGGGCT
8	CCCUUCCAGGACUCACCCCCUCACUUACCCUCCCCCUCACUUACCCUCCCC	GCGCCGGCGCGCGCGGGCGAGCTAGAGGGCGCGCGCGGCTGAAAGCGTGTGGA
9	CUCACCCCCUCACUUACCCUCCCCCUCACUUACCCUCCCCCUCAAUUCUCAC	CGCCGGCGCGCGCGGGCGAGCTAGAGGGCGCGCGCGGCTGAAAGCGTGT
10	UCCCUUCCAGGACUCACCCCCUCACUUACCCUCCCCCUCACUUACCCUCCCCCUCAAUUCUCAC	AGCCCGCGCCTCCACACGCTTTCAGCCGCGCGCGCCCTCTAGCTCGCCCGCGCGCGCCGGCGCC
11	CUCCCUUCCAGGACUCACCCCCUCACUUACCCUCCCCCUCACUUACCCUCCC	ACACGCTTTCAGCCGCGCGCGCCCTCTAGCTCGCCCGCGCGCGCCGGCGCC
12	CCUCCCUUCCAGGACUCACCCCCUCACUUACCCUCCCCCUCACUUACCCUCCCCCUC	CCGCGCCTCCACACGCTTTCAGCCGCGCGCGCCCTCTAGCTCGCCCGCGCGCGCCGG
13	UCUUCCUCCCUUCCAGGACUCACCCCCUCACUUACCCUCCCCCUCACUUACCCUCCCCCU	GCCGGCGCGCGCGGGCGAGCTAGAGGGCGCGCGCGGCTGAAAGCGTGTGGAGGCGCGGGCT
14	UUCCUCCCUUCCAGGACUCACCCCCUCACUUACCCUCCCCCUCACUUACCCUCCCCCUCAAUUCUCAC	AGCCCGCGCCTCCACACGCTTTCAGCCGCGCGCGCCCTCTAGCTCGCCCGCGCGCGCCGGCGCC
15	CCCUGCCCUUCCCCUCGUGUCUUCCUCCCUUCCAGGACUCACCCCCUCACUUACCCUCCCCC	CCTCCACACGCTTTCAGCCGCGCGCGCCCTCTAGCTCGCCCGCGCGCGCCGGCGCC
16	GACCCUGCCCUUCCCCUCGUGUCUUCCUCCCUUCCAGGACUCACCCCCUCACUUACCCUCCCCC	AGCCCGCGCCTCCACACGCTTTCAGCCGCGCGCGCCCTCTAGCTCGCCCGCGCGCGCCGGCGCC
17	CCCUUCCCCUCGUGUCUUCCUCCCUUCCAGGACUCACCCCCUCACUUACCCUCCCCCUCACU	CGCCGGCGCGCGCGGGCGAGCTAGAGGGCGCGCGCGGCTGAAAGCGTGTGGAGGCGCGGGCT
18	CCCUUCCCCUCGUGUCUUCCUCCCUUCCAGGACUCACCCCCUCACUUACCCU	CGCGGGCGAGCTAGAGGGCGCGCGCGGCTGAAAGCGTGTGGAGGCGCGGGCT
19	CCUGCCCUUCCCCUCGUGUCUUCCUCCCUUCCAGGACUCACCCCCUCACUUACCCUCCC	GCCTCCACACGCTTTCAGCCGCGCGCGCCCTCTAGCTCGCCCGCGCGCGCCGGCGCC
20	AAACUUAAAGUGUAACAAUAAUAAAAUUUUUUAAAAAAAAAAAAAAAAAAA	GCCCGCGCCTCCACACGCTTTCAGCCGCGCGCGCCCTCTAGCTCGCCCGCG
21	GCCGGACGCUUAUGGACGAUGCAUAUGUGGGGCCGGAUAGAAUUGUGGCAGGCGGCGCUGGGG	CCGGCGCGCGCGGGCGAGCTAGAGGGCGCGCGCGGCTGAAAGCGTGTGGAGGCGCGGGCT
22	GGGGUGUGCACCUCGGCCACGUGGAAAAGCGAGAAUGCAGAGGCCGGACGC	TCCACACGCTTTCAGCCGCGCGCGCCCTCTAGCTCGCCCGCGCGCGCCGGC
23	GGGUGCCGCUGGCACCCGGGAAGACGCUGGGGGCCGGCGCUGUAGAGCCGGGCAUGGGCUGGG	GGCGCCGGCGCGCGCGGGCGAGCTAGAGGGCGCGCGCGGCTGAAAGCGTGTGGAGGCGCGGGC
24	CGAGAGGCCUGACCCUGACGGGUGGGGGAGAUGCGCGUGCGGAGUAGCGGG	CGCTTTCAGCCGCGCGCGCCCTCTAGCTCGCCCGCGCGCGCCGGCGCC

Table S7 (related to Figure 6A): GSEA of genes responsive (up- or downregulated, |fold change| ≥ 1.5) to MCT1 knockdown in SUM149 cell line (from GSE76675) and to SLC16A1-AS1 knockout in UMUC-3.

GSEA of SUM149.shMCT1(up, down) vs. UMUC-3-KO (up, down)						
**Gene Set Name**	**# Genes in Gene Set (K)**	**# Genes in Overlap (k)**	**k/K**	**p-value**	**FDR q-value**	**$-log_{10}$ (p-value)**
GO_RESPONSE_TO_ENDOGENOUS_STIMULUS	1648	84	0.051	3.67E-26	2.72E-22	25.44
GO_NEGATIVE_REGULATION_OF_RESPONSE_TO_STIMULUS	1655	82	0.0495	9.87E-25	3.65E-21	24.01
GO_RESPONSE_TO_OXYGEN_CONTAINING_COMPOUND	1616	80	0.0495	4.19E-24	1.03E-20	23.38
GO_APOPTOTIC_PROCESS	1980	89	0.0449	6.26E-24	1.16E-20	23.20
GO_CELLULAR_RESPONSE_TO_ENDOGENOUS_STIMULUS	1384	72	0.052	6.11E-23	9.04E-20	22.21
GO_LOCOMOTION	1943	86	0.0443	1.12E-22	1.39E-19	21.95
HALLMARK_MTORC1_SIGNALING	200	29	0.145	1.81E-21	1.92E-18	20.74
GO_REGULATION_OF_CELL_DEATH	1723	78	0.0453	3.78E-21	3.5E-18	20.42
GO_CELL_CYCLE	1847	80	0.0433	1.59E-20	1.31E-17	19.80
GO_CELL_MOTILITY	1719	76	0.0442	5.11E-20	3.78E-17	19.29
GO_CELLULAR_RESPONSE_TO_OXYGEN_CONTAINING_COMPOUND	1126	59	0.0524	4.85E-19	3.26E-16	18.31
GO_RESPONSE_TO_NITROGEN_COMPOUND	975	54	0.0554	1.54E-18	9.49E-16	17.81
GO_NEGATIVE_REGULATION_OF_SIGNALING	1394	65	0.0466	2.49E-18	1.35E-15	17.60
GO_RESPONSE_TO_ORGANIC_CYCLIC_COMPOUND	917	52	0.0567	2.56E-18	1.35E-15	17.59
GO_REGULATION_OF_CELL_POPULATION_PROLIFERATION	1708	72	0.0422	7.20E-18	3.55E-15	17.14
GO_RESPONSE_TO_HORMONE	977	53	0.0542	7.95E-18	3.67E-15	17.10
GO_POSITIVE_REGULATION_OF_MOLECULAR_FUNCTION	1756	73	0.0416	8.73E-18	3.80E-15	17.06
GO_RESPONSE_TO_DRUG	1018	54	0.053	1.00E-17	4.13E-15	17.00
GO_PROTEIN_PHOSPHORYLATION	1967	77	0.0391	2.82E-17	1.10E-14	16.55
GO_RESPONSE_TO_EXTRACELLULAR_STIMULUS	523	38	0.0727	4.08E-17	1.51E-14	16.39

Table S8 (related to Figure 6B): Common differentially regulated genes (|fold change| ≥ 2) upon SLC16A1-AS1 overexpression in RT-4 cell line or knockout in UMUC-3 cell line.

RT-4 up & UMUC-3 down	RT-4 down & UMUC-3 up
**ARRDC3**	ACTL6A
**ATL1**	ADGRL2
**C5orf15**	ARCN1
**CARF**	ASNS
**COMMD7**	AURKA
**CYBRD1**	BAZ1A
**DPY19L4**	CDC20P1
**DRAM1**	COPG1
**ERAP2**	CSE1L
**GBP3**	FGD6
**KATNAL1**	GFPT1
**LPCAT2**	GNL2
**NRIP1**	HMGB3
**PHKB**	HSPA4L
**PPARA**	INTS4
**RAB23**	KIF2C
**RBBP9**	KYNU
**RP11-472B18.2**	LINC00958
**SAMD9L**	MCM2
**SCAMP1**	MICB
**SH3BGRL**	MTHFD2
**SNAI2**	MYB
**SNORD116-14**	NAV2
**SNORD116-15**	NCAPD3
**SNORD116-16**	ODC1
**SNORD116-17**	ORC1
**SNORD116-18**	RNF168
**SNORD116-19**	RRM1
**SNORD116-20**	SBNO1
**SNORD116-21**	SLC3A2
**SNORD116-24**	SLC7A11
**SNORD116-3**	TFRC
**SNORD116-9**	TM4SF1
**SYTL4**	TNFAIP3
**TCP11L2**	UBE2C
**TGFBI**	UTP18
	WDHD1
	XPOT

Table S9 (related to Figure 6B): Pathways and corresponding target genes differentially regulated by SLC16A1-AS1 identified using KEGG Mapper tool.

KEGG pathway	SLC16A1-AS1 targets
**Metabolic pathways**	ASNS, GFPT1, KYNU, LPCAT2, MTHFD2, ODC1, RRM1
**Ferroptosis**	SLC3A2, SLC7A11, TFRC
**NOD-like receptor signaling pathway**	GBP3, TNFAIP3
**Ribosome biogenesis in eukaryotes**	GNL2, UTP18
**Glucagon signaling pathway**	PHKB, PPARA
**Insulin resistance**	GFPT1, PPARA
**Alanine, aspartate and glutamate metabolism**	ASNS, GFPT1
**Cell cycle**	MCM2, ORC1
**Glutathione metabolism**	ODC1, RRM1

Table S10 (related to Figure 6F): Construction of the consensus position weight matrix (PWM) of E2F1 from the three known PWMs of E2F1 (MA.00024.1, -2, -3; JASPAR).

matrix position	1	2	3	4	5	6	7	8	9	10	11	12
MA.00024.1	T	T	T	**G**	**G**	**C**	**G**	**C**				
	0	0	0	0	0	0	0	0				
	0	0	0	4	2	10	0	9				
	0	0	0	6	8	0	10	1				
	10	10	10	0	0	0	0	0				
MA.00024.2		C/G	G	**G**	**G**	**C**	**G**	**G/C**	**G/C**	**A**	G/A	G/A
		259	218	144	0	0	0	0	0	1059	508	305
		317	0	274	0	1059	0	337	286	0	0	269
		280	628	641	1059	0	1059	722	773	0	551	485
		203	213	0	0	0	0	0	0	0	0	0
MA.00024.3	T	T	T	**G**	**G**	**C**	**G**	**C**	**C**	**A**	A	A
	254	241	208	54	0	0	0	2	0	524	510	493
	115	46	36	71	32	599	1	588	577	171	80	59
	89	100	145	888	950	0	1009	17	46	20	58	112
	565	638	627	1	0	2	0	0	50	119	182	219
CONSENSUS (Sum)	T	T	T	**G**	**G**	**C**	**G**	**C/G**	**C/G**	**A**	A	A
	254	500	426	198	0	0	0	2	0	1583	1018	798
	115	363	36	349	34	1668	1	934	863	171	80	328
	89	380	773	1535	2017	0	2078	740	819	20	609	597
	575	851	850	1	0	2	0	0	50	119	182	219
CONSENSUS (%)	T	T	T	**G**	**G**	**C**	**G**	**C/G**	**C/G**	**A**	A/G	A/G
	24.6	23.9	20.4	9.5	0.0	0.0	0.0	0.1	0.0	83.6	53.9	41.1
	11.1	17.3	1.7	16.8	1.7	99.9	0.0	55.7	49.8	9.0	4.2	16.9
	8.6	18.1	37.1	73.7	98.3	0.0	100	44.2	47.3	1.1	32.2	30.7
	55.7	40.6	40.8	0.0	0.0	0.1	0.0	0.0	2.9	6.3	9.6	11.3

Figures

Figure S1 (related to Figure 2)

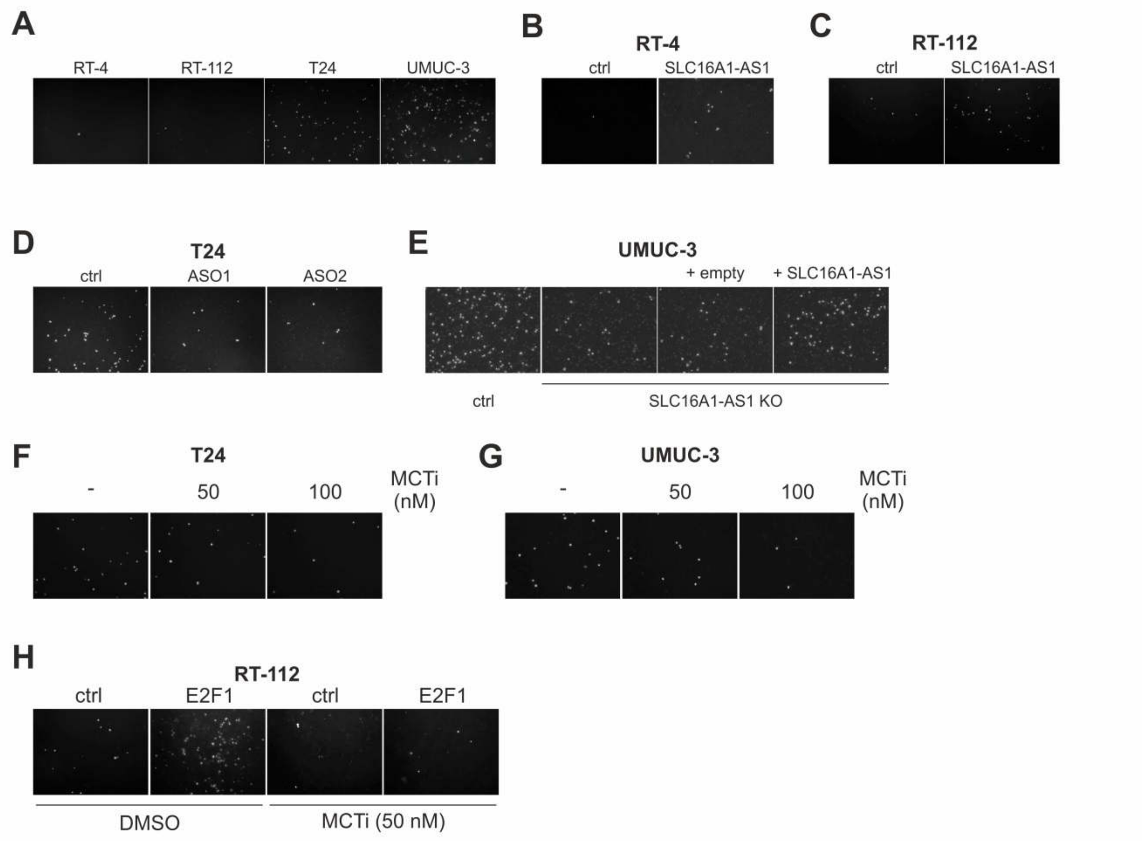

Figure S1. Representative images of migrated cells from Boyden chamber assays of indicated cell lines and conditions.

Figure S2 (related to Figure 3A)

HUMAN>NR_103743.1 Homo sapiens SLC16A1 antisense RNA 1 (SLC16A1-AS1), transcript variant 1, long non-coding RNA

```
GCGCGCTGCGCCCCTGCTGAGCGGCGCGGAGCTACCACGCCCTGCCAGACTCGCCCTTGGGTCCCTGTCTGACGAGCTCTAGG
CAATCTGCCCTCGTTCGGGACACAACCATCGGTCTCACCCCTCAGTTTCTGCCAGAGAAAAAGGAAAAGTCACCGAGAGGCCT
GACCCTGACGGGTGGGGGAGATGCGCGTGCGGAGTAGCGGGAAGCGACTGAGGAGCGGGGAATGGGCAGCATTTGAATGGATG
CGGGTGCCGCTGGCACCCGGGAAGACGCTGGGGGCCGGCGCTGTAGAGCCGGGCATGGGCTGGGATGTGTTTGGATTCCAATC
CGGGCCTGACACCAGTTCAGTGACCTCGGGAAGTTCCCCAACCCTCCGGGCCTGTTTCCTCCCTCTGAAGTGGCGACAGTAGT
AGAACCGACCTCGTAGGCTCATCGGGAGGTCCTGATGGGAGAACCCATGCAACTTGCCACCATAGAGCCAGGCCCGCGGCGGT
TGGCGCCTGGTGGGTATTAAAGACGAGTCGGGAAAGAAGAGCAGGTAAGAGGGTGGGGAGACTGGCCCAGTGGGTTGGGGTGT
GCACCTCGGCCACGTGGAAAAGCGAGAATGCAGAGGCCGGACGCTTATGGACGATGCATATGTGGGGCCGGATAGAATTGTGG
CAGGCGGCGCTGGGGCTGATGCTCCTCACCCGGATTATCCTCTCTAATCCTCAGCCCTGTGAAGTGGGCACTGCCCTGAACAT
TTTGTAAATAAGGGACCCGAGGCTCAGAGAGGTGAAGTAACTTTCCAGACAGCCCAGCTCGTCAGCGGCCAGCATTTGAAGTC
ACACTCCAGAGCCTGTTTTTAACATAATAGCCGCTGTGACCGCATAACCAACGTGTGTGTTGAAGTGATCAAATAACCATGGA
GTGTCTTTGATGTCCAGGACTTTTGAGAAATACCGAAAAATATGAGGCAAGGCCCCTGCCTTCAAAGAGCTTGCAAGTTTTTG
GGGGAGACTTAGGCACAAATTAACCAACATCTGTTGAGCACTTGCTGCTTGCCAGGACCCTGCCCTTCCCCTCGTGTCTTCCT
CCCTTCCAGGACTCACCCCCTCACTTACCCTCCCCCTCACTTACCCTCCCCCTCAATTCTCACAGCAACCCTGGGAGGTAGGC
CTTATGGTTTCTGTTTTGCAAATGAAGAACCTGAGATTGAAGAAGAGTAGAGATTGGAGGATCAGATCCTGAGAGGTCCCATG
TGCTTTCCAATAACAGTTCAGTATATTAGAAGACAGTGTGGCATCCACGGCCAAGTACTACTAGTTGATAGACTGGATGCTGC
TATGGTAATCTGCCTCAGGAAAATGCCGGACTGTTGTTTGCAAGCTGGTTAAGTGAGCAAATCTTGGGAAGATTTCAAGCACA
CCAACATGGCACATGTATACATATGTAACAAACCTGCACATTGTGCACATGTACCCTAAAACTTAAAGTGTAACAATAATAAA
ATTTTTTTAAAAAAAAAAAAAAAAAAAA
```

GORILLA>1 dna:chromosome chromosome:gorGor3.1:1:115899284:115901331:1

```
GCGCGCTGCGCCCCTGCTGAGCGGCGCGGAGCTACCACGCCCTGCCAGACTCGCACTTGGGTCCCTGTCTGACGAGCTCTAGG
CAATCTGCCCTCGTTCGGGACACAACCATCGGTCTCACCCCTCAGTTTCTGCCAGAGAAAAAGGAAAAGTCACCGAGAGGCCT
GACCCTGACGGGTGGGGGAGATGCGCGTGCGGAGTAGCGGGAAGCGACTGAGGAGGGGGGACTGGGCAGCATTTGAATGGATG
CAGGTGCCGCTGGCACCCGGGAAGACGCTGGGGGCCGGCGCTGTAGAGCCGGGCATGGGCTGGGATGTGTTTGGATTCCAATC
CGGGCCTGACACCAGTTCAGTGACCTCGGGCAGTTCCCCAACCCTGCGGGCCTGTTTCCTCCGTCTGAAGTGGCGACAGTAAT
AGAACCGACCTCGTAGGCTCATCGGGAGGTCCTGATGGGAGAACCCATGCAACTTGCCACCATAGAGCCAGGCCCGCGGCGGT
TGGCGCCTGGTGGGTATTAAAGACGAGTCGGGAAAGAAGAGCAGGTAAGAGGGTGGGGAGACTGGCCCAGTGGGTTGGGGTGT
GCACCTCGGCCACGTGGAAAAGCGAGAATGCAGAGGCCGGACGCTTATGGACGATGCATATGTGGGGCCGGATAGAATTGTGG
CAGGCGGCGCTGGGGCTGATGCGCCTCACCCAGATTATCCTCTGTAATCCTCACAGCCCTGTGAAGTGGGCACTGCCATGAAC
ATTTTGTAAATAAGGCACCCGAGGCTCAGAGAGGTGAAGTAACTTTCCAGACGGTCCAGCTCGTCAGCGGCCAGCATTTGAAG
TCACACTCCAGAGCCTGTTTTTATCATAATAACCGCTGTGACCGCATAACCAACGTGTGTGTGTTGAAGTGATCAAATAACCA
TGGAGTGTCTTTGATGTCCAGGACTTTTGAGAAATACCGAAAAAGTATGAGGCAAGGCCCCTGCCTTCAAAGACCTTGCAAGT
TTTTGGGGGAGACTTAGGCACAAATTAACCAACATCTGTTGAGCACTTGCTGCTTGCCAGGACCCTGCCCTTCCCCTCGTGTC
TTCCTCCCTTCCAGGACTCACCCCCTCACTTACCCTCCTCCTCAATTCTCACAGCAACCCTGGGAGGTAGGCCTTATGGTTTC
TGTTTTGCAAATGAAGAACCTGAGATTGAAGAAGAGTAGAGATTGGAGGATCAGGTCCTGAGAGGTCCCATGTGCTTTCCAAT
AACAGTTCAGTATATTAGAAGACAGTATGGCATCCACGGCCAAGTACTACTAGTGAGTAATACAGTAATTGCTTTTTACATTT
GATAAACACGGAGAAAGGGAAGGCATTTCGTGTCATTGGGGCAGCTAGGACTTATAAAAGCAGTGCTAGGGTGGCAGCAGCAT
TCAGGTAGATGGTAAGGAGAATTAAGTTAGAGAGACAGGATCCTCAGTCTGCTTCCGAAGTAAATATATAAGGGCCTGTTCTA
TGGATTGTAGTGAGAAGTGGTTAAATAAGATGAATCCCAGGAAACCTCAAAAGAAGGCTTATAGAGCCCCATAGGGTGGTAGA
GTCACACTATAGACCTGAGAGCCAGATCTGTTGAGTTCCAAATTTGATTCTTGCACTTACCAGCTCTGTGTCCTCAGGCAAGT
CATTTTACCTTTCCAAGCCTCACTCCCAACTCTGTAAAACAAGGATAATACCTACCTCAGGGTTATTATGATAATAAAACTAG
TATTTATTTCAGGCCGGCATGGTGGCTCAAGCCTGTAATCCCAGCACTTTGGGAGGCCGAGGTGGGCGGATCACTTGAGGCCA
GTATTTTGAGACTAGCCTGGCCAACATGGTGAAACCCTGCCAATACTAAAAATACAAAAATTAGCCATGTGTAGTGCATAGTA
CCAGCTACTCAGGAAGTTGAGACAGGAGAATTGTTTGAACCCGTGAGGCAGAGATTGCAGTGAGCCAATATCTCATCATTGCA
CTCCAGCCTGGGCAACAGAGTGCGATTCCATCTCAAAAAAAAAAAAAAAAAAAAAA
```

ORANGUTAN>1 dna:chromosome chromosome:PPYG2:1:115286199:115288261:-1

```
GCGCGCTGCGCGGGTGCTGAGCCGCGCGGAGCTACCGCGCCTGTCAGACTCTCCCCTGGGTCCTGTCTGACGAGCTCTAGGCA
ATCTGCCCTTCGTTTCGGGACACAAATGTCGATCTCACCCCTCAGTTTCTGCCATAGAAANNNNNNNNNNNNNNNNNNNNNNN
NNNNNNNNNNNNNNNNNNNNNNNNNNNNNNNNNNNNNNNNNNNNNNNNNNNNNNNNNNNNNNNNNNNNNNNNNNNNNNNNNNN
NNNNNNNNNNNNNNNNNNNNNNNNNNNNNNNNNNNNNNNNNNNNNNNNNNNNNNNNNNNNNNNNNNNNNNNNNNNNNNNNCAT
```

GGGCTGGGATTGTTGGATTCAATCTGGGCTGACACCAGTTCAGTGACCTCGGAAGTTCCCCAACCTCCGGGCTGTTTCCTCCC
TCTGAAGTGGCGACAGTAATAGACCGACCTCGTAGGCTCATCGGGAGGTCTGATGGGAGAACCCATGCAACTTGCCACCACAG
AGCCAGGCCCGCGGCGGTTGGCGCCTGGTGGGTATTAAAGACGAGTCGGGAAAGAAGAGCAGGTAAGAGGGTGGGGAGACTGG
CCCAGTGGGTTGGGGTGTGCACTCGGCCACGTGGAAAAGCGAGAATGCAGGGGCCAGACGCTTATGGACGATGCATATGTGGG
GCCGGTTAGAATTGTGGCAGGAGGCGCTGGGGCTGATGCGCCTCACCCGGATTATCCTCTGTAATCCTCACAGCCCTGTGAAG
TGGGCACTGCCATGAACGTTTTGTAAATAAGGGACCCGAGGCTCAGAGAGGTGAAGTAACTTTCCAGACGGCCCAGCTCGTCA
GCGGCCAGCATTTGAAATCACACTCCAGAGCCTGTTTTTAACATAATAGCCGCTGTGACCGCATAACCAACGTATGTGTGTTG
AAGTGATCAAATAACCATGCAGTGTCTTTGATGTCCAGGACTTTTGAGAAATACCTAAGAAGTATGAGGCAAGGCCCCTGCCT
TCAAAGAGCTTGCAAGTTTTTGGGGGAGACTTAGGCACAAATTAACCAACATCTGTTGAGCACTTGCTGCTTGCCAGGACCCT
GCCCTTCCCCTCGTGTCTTCCTCCATTCCAGGACTCACCCCCTCACTTACCCTCCCCCTCAATTCTCACAGCAACCCTGGGAG
GTAGGCCTTATGGTTTCTGTTTTGCAAATGAAGAACCTGAGATTGAAGAAGAGTAGAGATTGGAGGATCAGGTCCTGAGAGGT
CCCATGTGCTTTCCAATAACAGTTCAGTATATTAGAAGACAGTATTGCATCCACGGCCAAGTACTACTAGTGAGTAATACAGT
AATTGCTTTTGACATTTGATAAACACGGAGAAAGGGAAGGCATTTCGGGTTATTGGGGCAGCTAGGACTTATAAGAGCAGTGC
TAGGGTGGCAGCAGCATTCGGGTAGATGGTAAGGAGAATTAAGTTAGAGAGACACGATCCTGAGTCTGGTTCAGAAGTAAATT
TATAAGGGCCTGTTCTATGGAATGTAGTGAGAAGTGGTTAAATAAGATGAATCCCAGGAAACCTCAAAAGAAGGCTTATAGAG
CCCCATAGGGTGGTAGATTCACACTATAGACCTGAGAGCCAGATCTGTTGAGTTCCAAATTTGATTCTTGCACTTACCTGCTC
TGTGTCCTCAGGCAAGTCATTTTACCTTTCCAAGCCTCACTCCCAACTCTGTAAAACAAGGATAATACCTACCTCAGGGTTAT
TATGATAATAACACTAGTATTTATTTCAGGCCGGGGTGGTGGCTCAAGCCTGTAATCCCAGCACTTTGGGAGGCCGAGGTGGG
CGGATCACTTGAGGCCAGGATTTTGAGACCAGCCTGACCAACATGGTGAAACCCTACCAATACTAAAAATACAAAAGTTAGCC
GTGCGTAGTGCATAGTACCAGCTACTCAGGAAGTTGAGACAGGAGAATTGTTTGAACCCATGATGCAGGGATTGCAGTGAGCC
AAGATCTCATCATCGCACTCCAGCCTGGGCAACAGAGTGAGATTCCACCTCAAAAAAAAAAGAAAAGAAAA

MACAQUE>chromosome:Mmul_8.0.1:1:113937657:113939708:1

GCGCGCTGCGCCCCTGCAGAGCGACGCGGAGCTACCGCACCCTTCCAGACTCGCCCGTTGGTCCTTGTCTGACGAGCTCTAAG
CAACCTGCCCTCCTTCGGAACACAAATGTCGGTCTCACCCCTCAGTTTCTGCCAGAGAAATAGGAAAAGTCACCTAGAGGCCT
GACCCTGACGGGTGGGGGGAGACGAGCGCGCGGAGTAGCGGGAAGCGTCTGAGGAGCGGGGACTGGGCAGCGTTTGAATGGAT
GCGGGTGCCGCTGGCACCCGGGAAGACGCCGGGGGCCGGCGCTGTGGAGCCGGGCACGGGCTGGGATGTGTTTGGATTCCAAT
CCGGGCCTGACACCAGTTGAGTGACCTCGGAAAGTTCCCCAACCCTCCGGGCCTGTTTCCTCCCTCTGAAGTGGCGACAGTAA
TAGAACTGACCCCGTAGGCTCATCGGGAGGCCCTGAAGGGGGAACCCATGCAACTTGCCACCGCAGAGCCAGGCCCGCGGCGG
TTGGCGCCTGGTGGGTATTAAAGACGAGTCGGGAAAGAAGAGCAGGTAAGAGGGCGGGGAGACTGGCCGGTGGGTTGGGGTGT
GCACCTCGGCCACGTGGAGAAGCGAGAATGCAGGGGCCGGACGCCTATGGACGATGCGTGTGTGGGGCGGGATAGAATCGTGG
CAGGCGGCGCTGGGGCTGATGCGCCTCACCCGGATTAGCCTCTGTAATCCTCACAGCCCTGTGAAGCGGGCGCTGCCGTGAAC
ATTTTGTAAAAAGGGACCCGAGGCTCAGAGAGGTGAAGTAACTTTCCAGACGGCCCAGCTTGTCAGCGGCCAGCATTTGAAGT
CACACCCCAGAGTCTGTTTTTATCGTAATAGCCGCTTTGACCGGATAACCAACGTGTGTGTGTTGAAGTGATTAGATGACCAT
GGAGTGTCTCTGATGTCCAGGACTTTTGAGAAATACTGAAAAAGTATGAGGCAAGGCCCCTGCCTTCAAAGAGCTTGCAAGTT
TTTGGGGGAGACTGTCAGGCACAAATTAACCAACATCTGTTGAGCACTTACTGCTTGCCAGAACCCTGCCCTTCCCTCGTGTC
TTCCTCCCTTCCAGGACTCACCCCCTCACTTACCCTCCCCCTCAATTCTCATAGCAACCCGGGGAGGTAGGCCTTATGGTTTC
TGTTTTGCAAATGAAGAGCTTGAGATTGAAGAAGAGTACAGATTGGAGGATCAGGTCCTGAGAGGTCCTATGTGCTTTCCGAT
AACAGTTCAGTGTGTTAGAAGACAGTATGGCATCCACGGCCGAGTACTACTAGTAATACAGTAATTGCTTTTTACATTTACAT
AAACACGGAGAAAGGAAAGGCATTTCGGGTCATTGGGGCAGCCAGGACTTACAAGAGCAGTGCTAGGGTGGCAGCAGCATTCA
GGTAGATGGTAAGCAGAATTAAGTTAGAGAGAGAGGATCCTGAGTCTGATTTAGATGTACATTTATAAGGCCCTGTTCTATGG
AATGTAGTGAGAAGTGGTTAAATAAGATGAATCCCAGGAAACCTCAAAAGTAGGCTTATAGAGCCCCATAAGGTGGTAGATTG
ACAATACAGACCTGAGAGCCAGATCTGTTGGGTTCCAGATTTGATTCTTGCACTTACCAGCTCTATGTCCTCAGGCAAGTCAT
TTTACCTTTCCAAGCCTGACTCCCAACTCTGTAAAACAAGGATAATACCTACCTCAGGGTTATTATGATAATAAAACTAGTAT
TTATTGCAGGCCGGCGTGGTGGCTCAAGCCTGTAATCCCAGCACTTTGGGAGGCTGAGGTGGGCGGATCAATTGAGGCCAGGA
TTTTGAGAGCAGCCTGGCCAACATGGTGAAACCCTGCCAATACTAAAAATACAAAAATTAGCCATGCGTAGTGGCACACGCCT
GTAGTACCAGCTACTCAGGAAGTTGAGACAGGAGAATTGTTTGAACCCGTGAGGCAGAGATTGCAGTGAGCCAAGATCTCACC
ATTGTACTCCAGCCTGGGCAACAGAGTGAGATTCCATCTCAAAAAAAAAAAAAAAAAAAA

MARMOSET>chromosome:C_jacchus3.2.1:7:148928200:148930498:1

TCGCGGAAAGCCTGGTCGGGGCTTCCCATAGCCGCGCGTTCTGACTCATTCTCCCCTGCGTGTCTCCCGGGATCGTCGCGGCC
ACCTGCACGCGTTCGGGACAAACGTCGGTCTCACCCCTCAGTTTCTGGCAGAGAAAAAGGAAGAGCCACCGAGAGGCCTGCCC
CTGAGGGGTGGGGGACGATGCGCGCGCGGAGTAGCGGGAAGCGACTGGAGAGGGGGACTAGGGCAGCATTTGAATGGACGCGG
GTGCCACTGGGACCCCAGAAGACGGCTGGGGGCCGGCGCTGTGGAGCCGGGCTTGGACTGGGATGTGTTTGGATTCCATTCCG
GGCCTGACACCAGTTCAGTGACCTCGGGAAGTTCCCCATCCCTCCGGGACTGTTTCCTACCCCTGAAGTGGCGACAGTAACAG

AACCGACCTCGTAGGCTCATCGGGACGTCCTGATGGGAGACCCCATGCAACTTGCTCACCACAGAGCCAGGCCCGCGGCGGTT
GGCGCCGGGTGGGTATTAAAGACGAGTCGGGGCCGGGCGCGGTGGCTAACGCATGTAATCCCAGCACTTTGTGAGGCCAAGGC
GGGCATATCATCAGAGGTCGGGAGTTCGAGACCAGCTTGACCACCATGGAGAAACCGTGTCTCTACTAAAAATACAAAATTAG
CTGGGCGTGGTGGCTCATGCCTGTAGTCCCAGCTACTCGGAAGGCTGGAGCAGGAGAATCGCTTGAACCCGGGAAGCGGAGGT
TGTGGTGAGCCGAGATCGTGCCATTGCACTCCAGCCTGGGAAACAGAGTGAGAGACTCTGTCTCAAAATAAATAAATAATGAG
TAAGTAAATACGAATCAGGAAAGAAGGGCAGGTAAGACGGTGGGGAGACTGGCCCGGTGGGGAGGGGTGTGCACGTCGGCCAC
GTGGAGAAGCGAGAATGCAGGGGCCGGAGGCTTATGGGCGATGCCTGTGTGGATAGAATTGTAGCAGGCGGCGCTGGGGATGC
GTCTCACCCGGATTATCCTCTGTAATCCTCGCAGCCCTGTGAAGTAAGCACTGCCGTGAGCATTTTGTAAATAAGGGACCCGA
GGCCCAGAGAGGTGGAGTAACTTTCCAGACGGCCCAGCCAGTTAGCGGCCAGCATTTGAAGCCACCAGAGCCTGTTTTTATCG
TAAGAGCCGCCGTGACTGGATAACCAACGTGTGTGTGTTGAAGTGATCAAATAACCATGGAGTGTCTCTGATGTTCAGGACTT
TTGAGAAATACCGAAAAAGTATGAGGCAAGGCCCCTGCCTGCAAAGAGCTTGCCCGTTTTGGGGTGGGGGGGAGACTGAGTCA
GGCACAAATTAAGGAACATCTGTTGAGCACTTGCTGCTTGTCAGGACCCTGCCCTTCCCCTCAGTCTTCCTCCTTTACCCTCC
CCCTCAATTCTCATAAAAACCCTGGGAGGTAGGCCTTAGTTTGCAAATGAAGAACCCGAGATTGAAGAAGAGTAGAGATTGGA
GGATCAGGTCCTGAGAGGTCCCAGGTGCTTTCCAATAACAGTTCAGTATGTTAGAAGACAGTATGGCATTCATGGCCAAGTAC
TACTTGTGAGTAATACAGTAATTGCTTTTTACATTTGATAAACACAGAGAAAAGAAGTCACTGGGGCAGCCAGGACTTCTAAG
AGCAGTGCCAGGGTGGCAGCAGCAGTCAGGTAGATGGTAAGGAGAATTAAGTTAGAGAGACTGGAGAGTCTGGTTGAGATGTA
AACTTACAAGGGCCTGTTCTATGGTATGTAGTGAGAAGTGGTTAAATAAGGAGCCGCAAAAGAAGACTTACAGAGCCCCATAG
TGTGGTGATTCACACTATAGACCTGGGAGCCGGATCTGTTCAGTTCCAAATTTGATTCTTGCACTTACCGGCTCTATGCCCTC
AGGCAAGTCATTTTACGGTTCCAAGCCTCTCCACTCTGTAAAACAAGGGTAATATCTACCTCAGGGTTATTATGATAATAAAA
TTAGCATTTATTTCAGCCCAGCACAGTGGCTCAAGCCTCTAATTTCGGCACTTTGGGAGGCTGAGGCAGGTGAATCACTTGAG
GCCAGGAGTTGGAGACCAGCCCGGACATGGTAAAGCCCTGCCAATACTATAAATATAAGAATTAGCAAGGCATAATGGCACAC
GTTTGTAGCCCTAGCCACTCGGGAGGCTGAGACAGGTGAATTGCCTGAACGTGTGAGGCAGAGGTTGCAGTGAGTTGAGATCT
CACCACTGCATTCCAGCCTGGGCAACAGAGCAAGACTCCATCTCAGAAAAAAAAAAAAA

MOUSE>3 dna:chromosome chromosome:GRCm38:3:104637458:104638493:-1

GCGCGCCGGCGCCCCCCTTCCCGCCACGTGACCCGGCGCCGCGGCCCGGAGACCCCGCACATCCTTATCAGCGCTCTGAGGGG
GTAGCTTCTTTTGGCGCGTGCCGGGGACGCCATCGTGGGCCTCACCCCTCAGGCTCTGGAGGAGAAAAGGGGAAAAAAAAACT
GCCCTGAGGGCGGACCCTGAGGTGCAGAGGGCGAAGGCGATGGACCGTCGGAGTCCGGGGACTGAACAGCATTTTGAATGGAC
GGACACTGGTAGACTCCGATGTGGGCCTGGAACCCGGGCTCGGGGACCCTGGGAAGTTGCTGGAACCCTCCGGGCCTGTTTCC
TTGCGCTACATCAGCCACTATGGAGGCGTTAGGAAGTTTAATGTGCGATTTTACGCAAGTATCTCGTCACAGAGCCTGACCTG
TGGCATTTGATGAACGCTTAGTTTAAGGACAAGCCGGTCAGGAGAAACTGGCCCCGGGGCAGAGAGTGTGTACGTTGGAAACG
CTGATATGAGCATGCTCTTAGGCCAGAAGTTTATGAACACTGGATTTGGGGGACGGGTCGGGATTATGGCTAGTGGGATTAGG
ACAGTTGCATGCTAAGTAAGCACTGTACCAGAATTATCTTGTATGATCTTCACAACCGTATTAGGTGCCAAGAAGATCTTGTA
ACTAAGGAACCCCGGGCTTCACGAGGTTAACTGGCAGAAGTCACCAGGCTAGGAAGTCGCCAACATTTAAAGTCAAAGCCACA
GAGCCTGGTGTCACAAGAGCCATTTTCGGTTCTTCACCAAGCCCAGATTCCAAAGTGTCTTGAAGTGGTAAACTAGTGAGTAC
TCTTGATGGCCAGGACCCTTGATATTTTTATTATTACGAGATGGGTATTTGGTTGGTTGTTTTTGATTTCTTGTTTTTTTTTT
TTTTTTTTTTTTTTTTTTTGAGATAGCCTCGTGTGTGTGTATAGCTCCACCTGGCCTGGAACTCATCATGTAGATCAGGCTG
GCTTCGAACTAAGAGAGCAAACATGAAAAAAAAAAAAAAA

RAT>2 dna:chromosome chromosome:Rnor_6.0:2:207107357:207108366:-1

GCGCGCCAGCGCCCCCCCTTCCCGCCACGTGACTAGGCGCTGCGGCCCGGAGACCCCGCATCGCCCCGGGCGCTTATCCGCGC
GCTCTGGGCTAGCTGCTTTTGGCGCGTGCCCGGGACGCCATCGCGGGCCTCACCCCTCAGGCTCTGGAGGAGAAAAGAGAAAA
ACTGCCCCGAGACCGACCCTGAGGTGCAGAGGGCGATGGCGATGGACGGGCAGAGTCGAGGGACTGGACAGCATTTGAATGGA
CGCTGGTAGACTCCAACATGGGCCTGGAACCCAGGCTCGGGGACCTGGGGAAGTTGCTGGAACCCTCCGGGCCTGTTTCCTCG
CGCTACATCAGCCACTGTGGAGGCGTTAGGAAGTTTAATGTGCAATTTTACGCAAGTATCTCGTCACAGAGCCTGACCTGTGG
CATTTGATGAACGCTAAGTTTAAGGACAAGCCGGTCAGGAGAAACTGGCCCCGGGGCGGAGGGTGTGTACGCTGGAAACGCAG
TTCTGAGCATGCTCTCAGGCCAGAAGTTTATGAACACTGGATTTTGGGAACCGGTTGCAATTTTATAGCTAGTGGGATTAGGA
CAGTTTCATGCCAAGCACTGCACCAGAATTATCTTGTGTGATCTTCACAACCAAATGAGATAGGTGCTAAGAAGATTTTATAA
ATAAGGAACCCCGGGCTTCACGAAGTTAACTGGCAGAAGTCACCAGGCTAGGAAGTGGCCAACATTTAGTCAAAGCTACTGAG
CCTGATGTCATAAGAGCCATTTTCGGTTCTTCAACAAGCCCAGATACCAAAGTCTCTTGAAGTGGTAAACTAGTGAATATTAA
TGACTAGGACCCTTAATATTTTTATAGTATTATGATATGGGTATTTGCTTGGCTGGTTTTGATTTCTTATTTTGTTTTTTGAG
ATAGCCTCGTGTGTATATAGCTCCACCTGGCTTGGAACTCACTACGTAGATCAAGCTGGCTTCGAACTAAGAGATCAATCTGA
AAAAAAAAAAAAAA

DOG>17 dna:chromosome chromosome:CanFam3.1:17:62639193:62641707:-1

CCCTGCGCGACCCGCGCTGGGCGCCCCGGCTCCGCGCGCCTCTGCCGAGCCAGCCCCTCGGGTCCCCGGTCCCAGGAGTTCGC
GGTGACCTGCGCGCGTGCGGGGCCCCGGCCCCGGCCCCGGCCCCGGCCCCCGCCCTCAGCCCCGGGCGGAGCAAAGGGAAAAG
CCGCCGAGGACGGAGGGGCGCACCCCGGGGGCCGACTCAGGTGTGGGGGGCGACGGGCGCGCGGAGGAGCGGGGAAGCGCCTG
CAGAGGAGGGACGGGGCCGGGCGTGCGTCGGCTCGGGGCACCTCGGGCCCGGGAAGCGGCCGCGGGGCGGGCGGTGAGGGCGA
GGGCGAGGGCGCGGGCGCGGGCGCGGGCGCGGGCTGTGGCCTCCGCGGAGTTCGGGGAACTCGGGGAAGTCGCTCGAACCCTC
AGGGCCTGTTTCCTGTTCTTGAAAACGGCAACAGCAGTAGGACAGACCTTACGGGCGCTTTTGGTGGTTTCGATAAGAGAACT
GATTGAACTTCCTCCACGCAGAGCCTGCCAGCCTCCCGGGGCGCTGGACCAATGCTGGGCGCTCGTTACGCGCAGAAGTCGGG
GAAGAAGAGCAGGTCAGCGCGGGGCGCCTGAGGCGGGTCCCTGAGGAGAAGGGCTGTGAGTGTCGGAAACACCGAGACGCGAG
AACGCGGAGGCCAAGCTTTTAGGGACACTGAGCGTTCGGGACAGGATATAATTAGAGCCGGCAGTTTTGAGGGACAGTTCGCC
GGAATTATCCTCCTAACCGTATGAGGTAGCCGACTATTATTAACATTTCACAAATAAGGGGGCTTGAGGCTCAGAGAGGTTAA
GTGACTTCCCAGAGGCCACAGAGTAATTGGCCAACACCCAAAGTCAGACTCCAGAGCCTGGTTTTGTTCTCAGAGCCATTGTA
AGTTCTTGAGCAAGTTAGAGCACTGGAGGTGTTATCAGCTCAAGTGATCCGCTTCAGTAAACACGAGGTGGCTCTTATGTTGG
CTTTTGGGAAATAACCAAAAACGTATGAAGGCCTCCCTGCCTTCCAAGAGTCTGCAGGTTCTTTGGGGAGATTGAGTCAGGGA
GAAAACAACCGACACCGGATGACCACTTGCTATTTGCCCCCCAAACCAGCCCCTTCTACCCTGAATTGCCACAGCCCTGGGGA
GTCGGCCTTAAGGTTTTGTCTTGCAAATGAAGAACTGAGATTGAGGAGTGAGGATTGAGGGGTCAGGTCCCGAGATGTAAATC
TGCTTTACCCTTCACCCATTCTGTCTCTGTCTCTGTTTCTTTTTCTTTCTTTTCTTTTTTTTTTTTTTTTTTTAAGATTTTATT
TGTTTATTCACGAGAGACACAGGCAGAGGGAGAAGCAGGCTCCAGGCTGGGAGCCCAATCTGGGACTCGATCCTGAGACTTCA
GAATCACGCCCTGAGCAGAAGGCAGGGGCTCAACCGCTGAGCCCCCCAGGTGTCCCTCTGCCTCTGTTTCACACAAAGAAGGT
TTTCGGTGATAGTTCAGTGCCTCAGAAGACCCTATGGAATCTGTGGCCATGCAGTGCATGTGAGCGGTAACTGTTTTGCACCT
TGGATAAATGCAGAGGAGGAAGTCATTTCAGGATTTAGAACAGCAGTGCTTCAGGAATGATGGGGTGGTAGCAGCATTGATGG
TGGATGGTAAGAGAACTGAGTTAGGGAGACAAAATGGGACTGGTTCAGACGTGAACTTTATCAGAGCCAGAACTATAGAATGT
AGCGGGAAGTGGTAGAGTAAGTTGATTCCCAGGAAACCTGAATAGGAACGGAGTGGGGTAGCTTTATAATATCAACCCAGAAA
CCAGATCTGAGTTCCAATTTTGATACTTGGCATCTACAAGCTCTGTATTGTTAGACAAATTATTTTGCTCTCCAAGCCTCATT
CTCTGCTCTGTGAAACAAGGACAATACCTACCTCATAGGGTTTTTATAATAATAAAAATAGTATTTTACTTAAGTCTGGCATA
CAGTAAGCACTAAATAATTGTATAACTATGGAAAACCATGAAACTGGATCCTAGAGGTTAATCCTAAAAGCACTTCATTTGAA
TAGATTCCTTCCATGGCCCTAAAGGGGGCCCTAACAATGGGTTCACAGGATCATATTGGTCTTCTGTGGTAATTCTGTATTCC
TTTTCTTGAAAATGTCCACCAAAATTGTATACACCTCAGGCTGCACAAAATCTGGATCTGCTCTTAGTAGGAACTGTAGTGTT
GTAGAAAGAGTATAGATGTTGGAATGAGAAGGTTTGTTCCCATATCTTATTATTTGTGTTACTTTGGACAATTAATTCATTGA
ACTCTTCTGGGCCAGGTAAAATCAAGGTAATATATGCCAGCCTGAGCTGTTATGATAATCAAATAAGAGTGTTTGCAGAAGGG
GTTGGTAAAACTTGGAAAGCACTCGACAAGTGTTTGTGAGTGCTTACAATTACAATACAGTTAATATTGTAATGTGTTTATAA
AGTGCTTAAGAGGAAAAGGTCAGAA

COW>3 dna:chromosome chromosome:UMD3.1:3:30531596:30533610:-1

GCGCGCGGCGCCCCCGCTATGCGGCAGGGACCTTGTAGCCGCGCTCCTCTGCGGAGTCTCCCCTTCGGCCCGCCGTCGGAGGA
CCTCGGGGTAATCTACTCGCATCTGGGACACCAGCGTCGATCTCACCCTTAGTCTAACTGCCAAGGATGGATTGGGCGTGCAG
CCTGGGGCCTGACTCAGGTGTGGGGGGCGATGGTGCGCGGAGAAGCTGGGAACTGACTGGAAGTGAGGGATTGGGCGGGGTTT
GAATGGACGCGGGTGCATCTCGGACCGGGGAAGAGGCCGAAAGCCGGCAGTTTAGAGCGCGGGCTGCGGCCTCGCTTCTTGAG
ATCAGTGACCTCGGGGAAGTCACTCAAATCCTCAGGGCCTGCTTCCTGCTCTCTGAAATGAAAACAGTAATACAGGCGACCTC
ATAAGGGCTTTTGGTGATTTTGATGAGAGAATTTATGGAACTTTTTTACATTTGATGAATGGTGAGTATGAACACAGAAGACG
GGAGAAAAGAGCTGGTCTGACGGTGGTGACACTGGCCCCACGAGAGGATCTGTGTGTCGGAAATATGAAGAAATGAGACTGCA
GAGAGGCCAAATTTTTATGGACGCTGAGGGTTTGGAACAAAATATAATTATAATTAAAGGTTCTGAAGGGCCGATTATATACT
TAGCAGTTCACCTGAACTATCCTCAGGACCCTATGATGTTGAAGTGATCAAACTGAATAAACATGGAGTGCCTCTGATGTCCG
AGACTTTTGAGAAGTACCGAAAATGTATGGATCAAGGCGCCTAACAAAGATTTTGGGGGGAGGTCGAGTCAGATGGAGATAAA
CCAACGTTGACCAGCACAGGAAGCCTGCGTTCCTCTCCTCCCCTCACCCCTCCTTACTTAATTCTCTTTACAATCCCGAGTGG
TTCAGCCTTTTGTTTTGCAAATGAAGAATCTAGATTGTAATGGAATGGGCCCTGAGGGATACGATTCTGAAAGTTTCCAAATA
CTTTCTCCCTCTCACCCACTCCGCCTCTGTTTCAGGCTAGAAAAAGTTTTCAGTAACAGTTAAACATGTTAGAAAACAGCATG
GAATCTACAGCCAAGTGCTACTTATGAGCAATAATTGCTTTTTACCTTTGATAAATACAGAGAAGGAAGGCTGTTCAGGTCCT
TCAGTTGGTCAGGACTTCAGCAGCACTTCAGGACTGTGACGGGTATAAGCAGCATTCAGGGTAGATGGAAGAGAGTTAGGAAG
ACAAACTAGGACTGGTTCAGATATGAACTGTCAGGACCTGAACGGTGGAATGTAGGAGGAGCATAGTATGGTAGATTCACAAT
ATGGACCCAGGATCAGATCTGAATTCGAGCTTGAATACTTGGCACTTACCACGTCTTTGTCCTTAGGTAAATCACCCCCGCTC
CCACGCCCCCGCCGCTGCTCTGAGCCTCATTCATTCTCTGCTTTGTAAAACAAGGACAGTACCTTCCTCATAAGGTTGTTATA
ATAATAAAATTAATATTTACTTTAGTGTCTGGCATATAGTAAGAACTAAATAAATGTATAACTAGGGGAAACTGTGAAATTGA
ATGCTAAAGATTAACCCTAAAGTTCCCTCACTTGGTCCTAACAGTGTAGTCACAGGATCATACTTAGTGATGTATTGTACTTT
TGTATTCCTTTTCTTGAAGATGTCCACCAAAAATATATATACCACAAGCCCCATAAAATCTGGATCTGCCCCTAGTAGGCTTT
CTAATGTGAAAAGAATACAGGCTTTGGAATCAAAAGACTTGTTCCCAAAACTTATCCCTGTTACTTTGGGCAATTAATTCACT
GACCTATTCTGGGCCTGTTTTTTTTAACCAGGTAATTTGAAGATAGTATCCATCAATCTGAATTGTTGTGACAGTCAGGTAAG

ATAGTATTTGCAAAAGTGATTGGTGGAACTTGATAAGTGCCCTATAAATGTTTATGAATTAATATTATAATTTTTCCACAAAG
TTTATAAAAGGCAAAGATAAAAA

OPOSSUM>chromosome:BROADO5:2:483438127:483440716:1

AGCCTCAGGCACTTACTAGCTGCATGACCCTGGGCAAGTCACTTAACCTCGTTTGCCTCAGTTTCCTCCTCTGTAAAATGAGC
TAGAGAAGTAAATGGCAAACCACTCTAATCTCTGCCTAGAAAACCCCAAATGGAGTCACAAGTTGAACATGAACTCAACAATG
AGAATGGTAAAAGGTTTCCAGTGCAAACTGCAAAAATGCAACAGCTTTGAAGACACTTATTGTTCCCATTAATCGGGACCTGG
CTCCAATTCTTTGTAGAGGCTGTAAATTCCATTAGATCACCACCGTTCTGTACAGTCTAGGCAGTAGCCATTAGGATCAATTT
AAAATTCGTTCCCAGCATGGCCCAGGAAGCTGTGTTCCCTTGAAAGCAATGATGCATGCAAAGCTCGGGTGCTCCGAGTTCAA
ATCTAGCCTTTGGCCGTTCGTTACCCGAGTGATCTTGGGCTCGAGCTACGGGCCTCAGTTTCCTCCAGAGTAAAAAAGGGAGG
AGCCTGACCACCATGCCTCCCCAGTCTCTTCCCGCGCTCTCGAAGTGGAGTGACAAAAAGGCCTTTCTGGCAGTCCCAGGCAG
AAGGTTAGGCTCATCCATCATCCCGCCTCGGCCCCACGCTAAGCCCCACTGCTAACGTCGGGGCAGGGAATGCCTGGAAGCCG
GACGGGGCGCCACGTGGAGGAGGCCTCTCCAATCGCGGCCAGAAGAAACTAGGAGGGCTAGTACCATTCCGCGTGCCAGTGTG
GGAGCACTGGGGGCCTTTCCCTGGGTCCCCACCACTCCACAGGTGCCCGCCCGGGCCGCCTCCCTTTGTGCGAGGGCGGGAAG
GCGCCACCCCTTAGACCACCCCCCCTCCCTCACCGGGCCGATTGGCGTCTCGCCGCCCTTCCTTTTACCTCCCTCCTTCCCTC
GTTCCTCGCCCAGGCCCCCGCTAGCTCGGGATGCGGGCACGGAGGAAACGCGCGCGCGCGCGGACCGAACAAATGACCGCTTA
CGAGGCAGCCGCTACCCAACTAACCGTTATGTGCGCGACCGGGAGAGAGAGCGTGGGCGTGAGGGGAGAGCTCGCGATCCGCT
GGGCTCCGGCTCGGGTCACGTCACTCCGCCGCTCGCTCACTCCCTCCCTCGGCAGGATTAGCGGCAGCGGCCCCGAGCCCGCG
GTGCTCTCTCCCGTCGGAAGCGCCCGCTCAGCTCTGACCACCGCTGCCTCCTGCCACTGGCTTATAATGCACCCGGGGCGGGA
GCATGCGCACAGGGGGCGGAGCATGGTCCGCCCCGCCCTCTCCCCGCACCCCTCCCAAGACTCACGTCTGCACACAGACACAC
GAACATGCGGCTCCCCACGACCCGACCACGTGATCCCTACTGGGAAGAGCTGCTCATCAGGGCAACCCCACACGGGTGTCGGA
CCTCCTCCACGGAGGACACCCATGAGATGGTTACCCGCCCCTGGGGTGGGAGGTGTGCTTAACCTCCCAGTGCCTCCTGTTTC
CCACTCCAAGCTTGAAGTCACTTAATGTCTACACTTGCCAGAGCAGGAAATTTCCCCCACTCCGCTGCTCATGACATGGAAAG
TTTACACAGATGGATGCTCAGGAATCGTCGAAGAGACACGCTGCGTGGAACACAGGGGTTTGGAAGGATTCTAGGGAAGGCAG
TGCACAGCCGTGGAGTGCTTAAGCTAAACGCTAGGGAAGGCAAAGGAGCTCCCTTTTTAAGCAGGAAACTATGTAAATAGGTA
CTTGCAAGACAGACACAGAGCAGCGTTGGTTGGGTCGTTGCAGTCGGGTCTGATTCCTCAAGACGCCATCTGGTTTTCTTGGC
AGAGATACTGGAGTGGTCTGCCCCATCCCAGCTCATTTTACAGATGAGGAAACTGAGGCAAACAGGCTTAAATAACTTGTCCA
GTGTCACACAGCTAGTATTTTCCATTTCCTTCTCCAACCTATTTTACAGATGAGGAAACTAAAGCAAACAGGATCAAATGACA
TGCCCAGGGTCACACAGTAAGCATCTGAGATCAGATTTGAATGCAGGTTTTCCTGATTCCAGGTTGAGCACTGGTATCTACTG
TGCCACCTAGCTGCCCCAGATAGAGTAATCTCATGGGAGAAAACCTGATATAGAATTTGGGGCTTGGACTGAGTCCAGAAGCA
TGCCAGGAAAAAGGCCGGAGGTGAGTATTCCATCCATTAAAAGAGGAAGGAGGACACCCAGTGCAAAATTATGGAAACAGGAG
ATGGAATAACCTCCGTAAAGAGCAGCAGGAATGATTGTATCTCTGGATCACAGTGTCTGAAAGGGAGAAGGCGAGAAAAGACA
AGGATTCAAGTTACAAAGAACTTCCTTACATTGCCAAATACAGCATTTTATATAGTTGATCCTGGAAAACATTTAAAAATAAA
TATATATGTATATATTTAATCCTGGAGCTAATGGAGTTATTGAAAGGGATGTGGAGGTGACACATGGCCAGATCTACACTTGA
GGAAAGTGACTTTGGCACCTAAATTTGGGGAGTGATTTGAGGTAATCCGCAGGCCAATTAGAAGGCTATTTCATTTGTCCAAA
AGGGCTATCATGAGGAA

Figure S2. Sequences of SLC16A1-AS1 homologues in representative eutheria and metatheria species.

Figure S3 (related to Figure 3A)

```
HUMAN        ---ccctccccgccacacagacatccgaact-gcagcccgcgcctccacac-------------------
GORILLA      ---ccctccccgccacacagacatccgaact-gcagcccgcgcctccacac-------------------
ORANGUTAN    ---ccctccccgccacacagacatccgaact-gcagcccgcgcctccacac-------------------
MACACA       ---ccctccccgccacacagacatccgaact-gcagcccgcatctccacac-------------------
MARMOSET     ---attacactccagcctgggtaacaagagc-gaaactc-cgtctcaaaaaaaaaaaaaaaaaaaaaaaa
COW          ---ccctcccccctacacttacacccgaacc-gcgactcgcgcctccaca--------------------
DOG          ---ccccccccccccccccggacgccaaatc-gcgactcgcg-ctccaca--------------------
MOUSE        ---ccctccctgccatacatactcccagctcggcatctcgcgcctccacac-------------------
RAT          ---ccctccctgccatacatacaccctgctcggcatctcgcgcctccacaa-------------------
position in block: -131 to -85

HUMAN        --------------------gctttcagccgcgcgc--------gccctctagctcgcccgcgcgcgccg
GORILLA      --------------------tctttcagccgcgcgc--------gccctctagctcgcccgcgcgcgccg
ORANGUTAN    --------------------gctttcagccgcgcgc--------gccctctagctcgcccgcgcgcgccg
MACACA       --------------------gctctcagccgcgcgc--------gccctctagctcgcccgcgcgcgccg
MARMOSET     aaaagaagaagaagaagggacctctccgctgcgtgat----aacgcgccccgggccgagcctgcgctccg
COW          --------------------gctctcaggcgcgcgcccgcagagtcgctttctctcgctcgcgcgcgctg
DOG          --------------------cgtttgaggcgcgcgc--------cgcccgcgctt----tgcgcgcgccg
MOUSE        --------------------gctgcgggctgcgcgt--------gcccgctcgctcgctcgggcg-----
RAT          --------------------gctgcggtctgcgcgt--------gcccgctcgctcgctcgggcg-----
position in block: -84 to -43

HUMAN        gcg-ccccctccccgccacgtgaccggcgtcccagtccg-accc
GORILLA      gcg-ccccctccccgccacgtgaccggcgtcccagtccg-accg
ORANGUTAN    gcg-ccccctccccgccacgtgaccggcgtcccagtccg-accc
MACACA       gcgcccccctccccgccacgtgaccggcgtcccagtccg-accc
MARMOSET     ggg-gcggagtcggccctcgcgctcgctgctctggcagacaggc
COW          gcgcccccctccccgccacgtgacccgcggcccggcaag-accc
DOG          ccg-ccccctcccagccacgtgacccgcg--------------g
MOUSE        -------------------------------------------c
RAT          -------------------------------------------c
position in block: -42 to -1
```

Figure S3. SLC16A1-AS1 promoters from depicted species bear putative E2F1 binding sites (highlighted in yellow).

Figure S4 (related to Figure 3E)

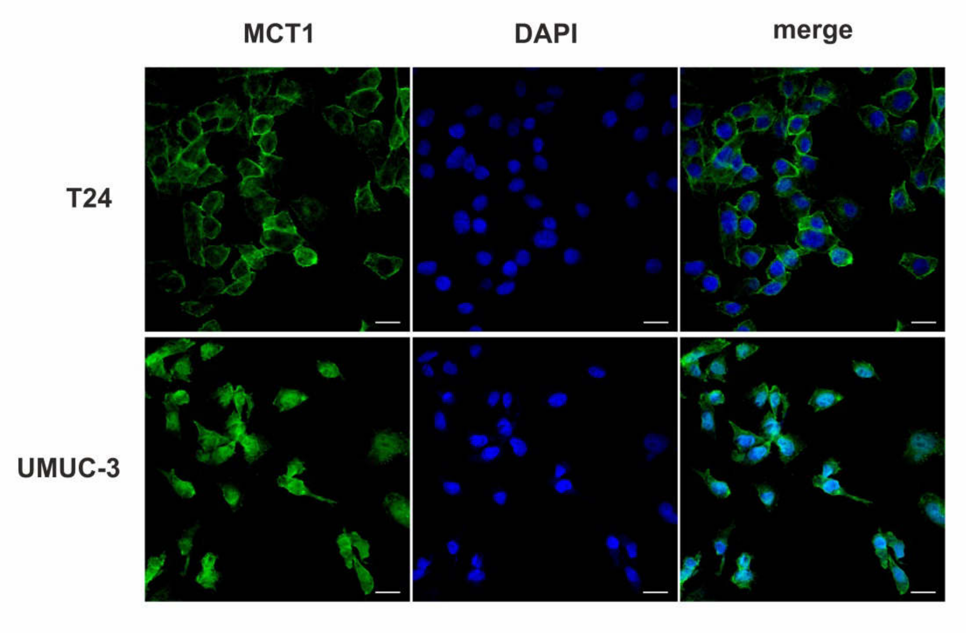

Figure S4. Immunofluorescence staining of MCT1 in bladder cancer cells. MCT1 (green) localizes in the cytoplasm and the cell membrane. DAPI (blue) represents nuclei. Bar graph: 20µm

Figure S5 (related to Figure 4A, B)

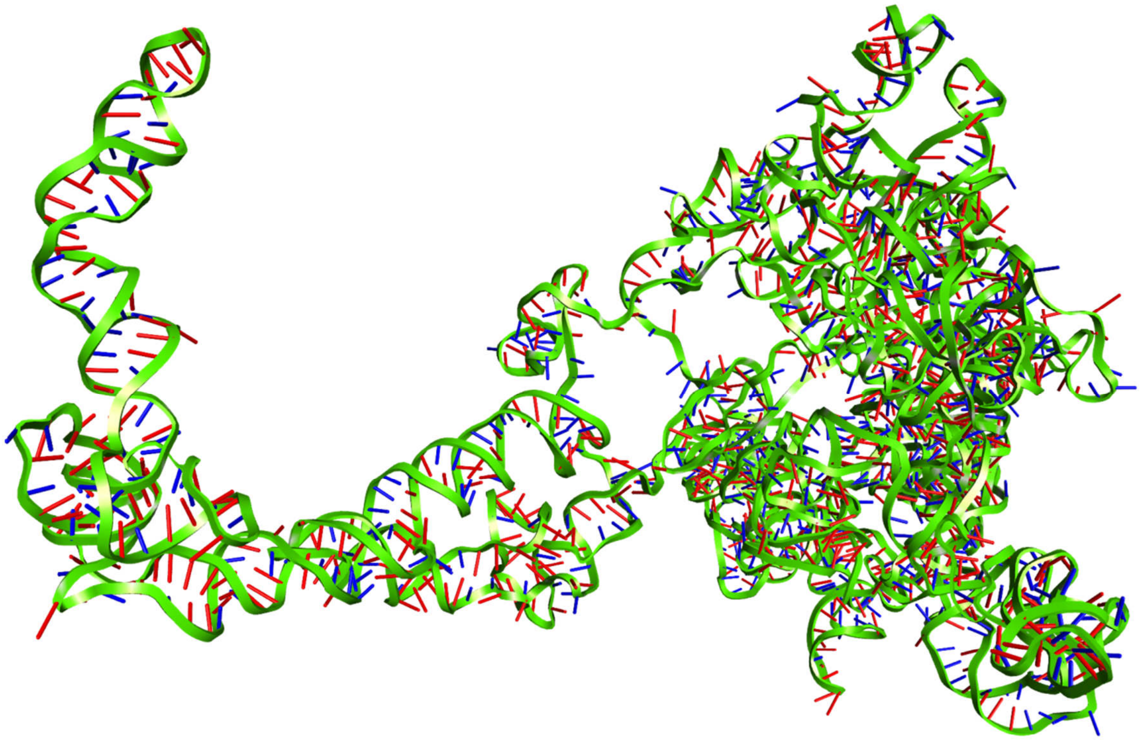

Figure S5. 3D model of SLC16A1-AS1. Based on secondary structure folding a 3D model was generated using 3DRNA v2.0 software tool which is based on a fragment assembly approach to build RNA 3D structures. After manually cross-checking for bond length and missing interactions the model of SLC16A1-AS1 was optimized using 'Smart Minimizer protocol' available in DS2017 using CHARMM force field.

Figure S6 (related to Figure 4F, G)

A

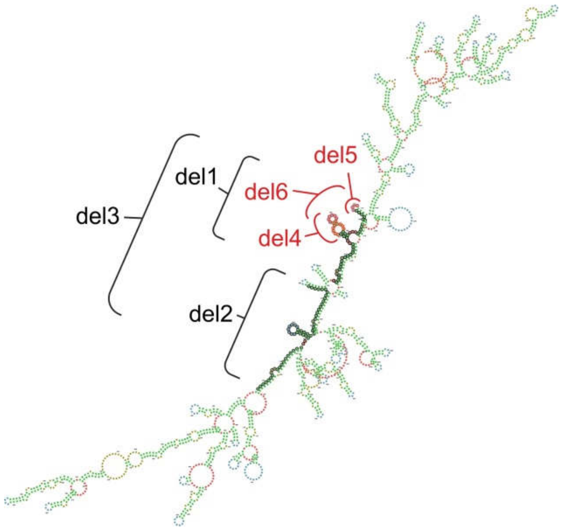

B

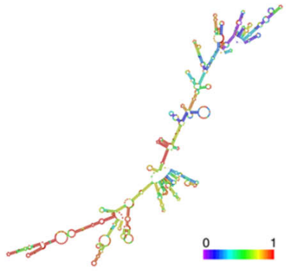

Wild Type SLC16A1-AS1 lncRNA. Minimum Free Energy: -525.60 kcal/mol

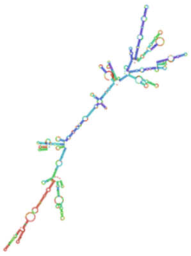

del1: bases 172-233
MFE: -492.30 kcal/mol

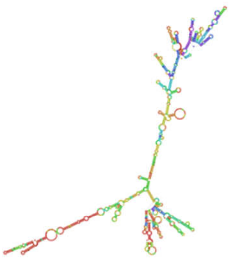

del2: bases 262-323
MFE: -480.70 kcal/mol

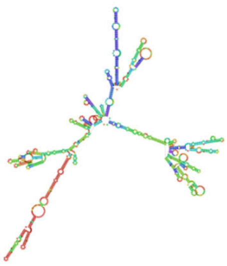

del3: bases 172-233 & 262-323
MFE: -449.70 kcal/mol

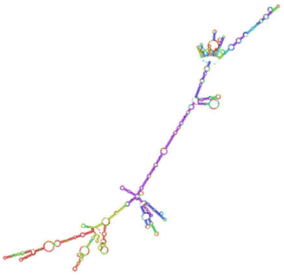

del4: bases 193-209
MFE: -518.50 kcal/mol

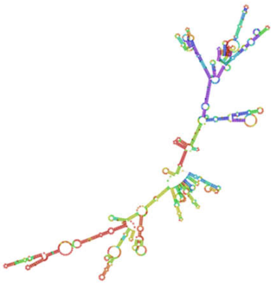

del5: bases 172-175
MFE: -524.80 kcal/mol

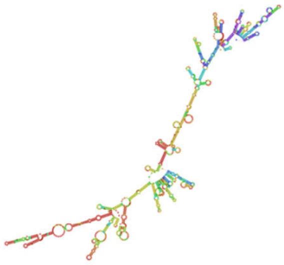

del6: bases 172-175 &193-209
MFE: -517.30 kcal/mol

Figure S6. (A) Positions of the deletions in SLC16A1-AS1. (B) Minimum free energy (MFE) fold structures from wild type and *in silico* mutated SLC16A1-AS1. In all deletions MFE per mol of the lncRNA is reduced in comparison to wild-type. We calculated number of hairpin, interior and multiloops (junction) with at least three bases. For all mutants, the numbers of hairpin loops are reduced. In *del1*, *del2*, *del4* and *del6* the amount of interior loops is slightly increased in comparison to wild-type.

Figure S7 (related to Figure 4F, G)

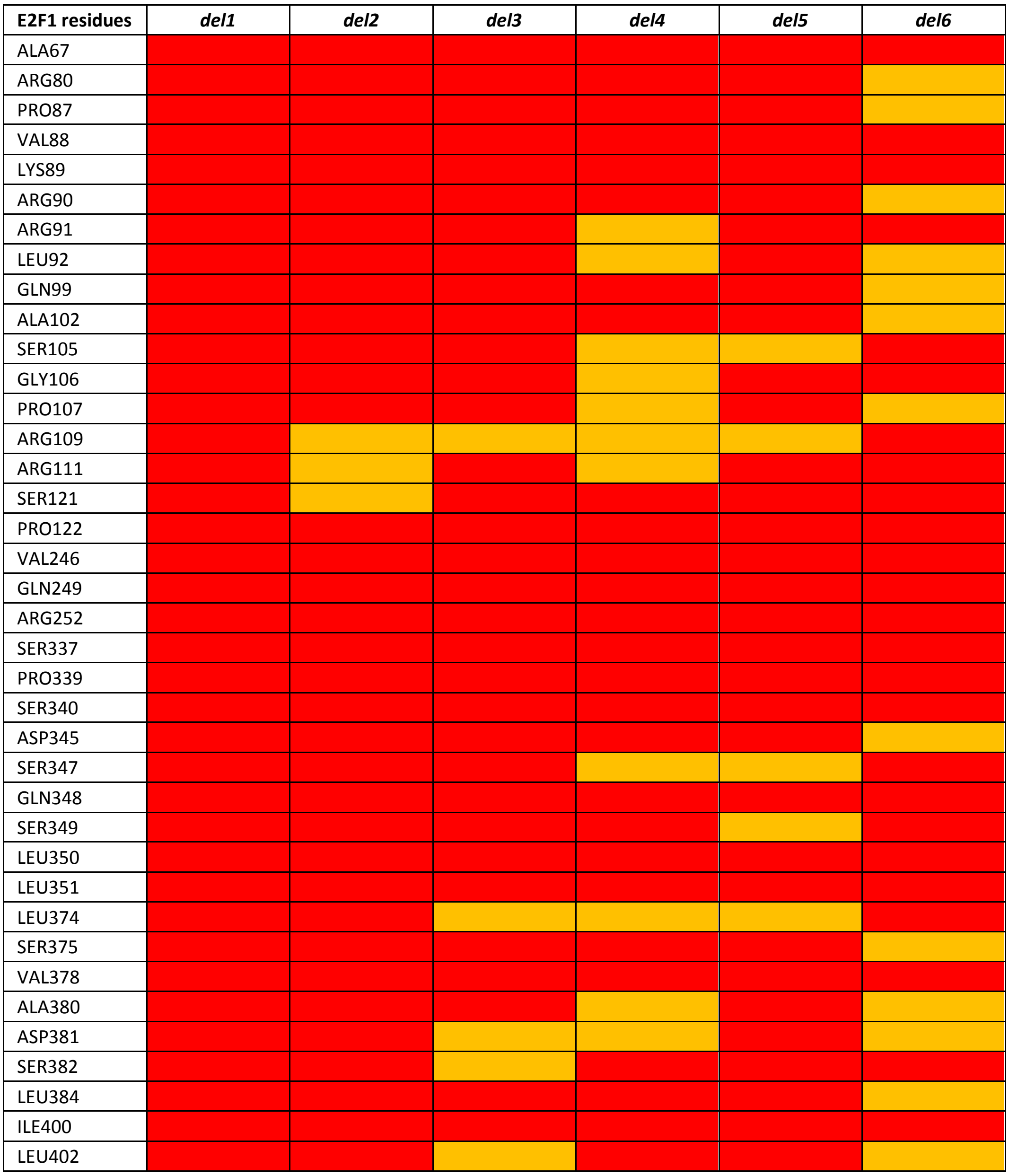

Figure S7. Pattern of E2F1 interacting residues with SLC16A1-AS1 wild-type compared to the mutants. E2F1 residues that are interacting with wild-type SLC16A1-AS1 appear in the first column. Orange indicates that the residue is involved in the same wild-type specific lncRNA interaction with E2F1whereas red shows non-interacting residues. All deletions lead to a strong reduction of protein:RNA associated groups. Especially, for *del1* no binding was predicted between E2F1 residues and the mutated lncRNA model, supporting a relevant role of this region for a functional E2F1 binding.

Figure S8 (related to Figure 5A, B)

A

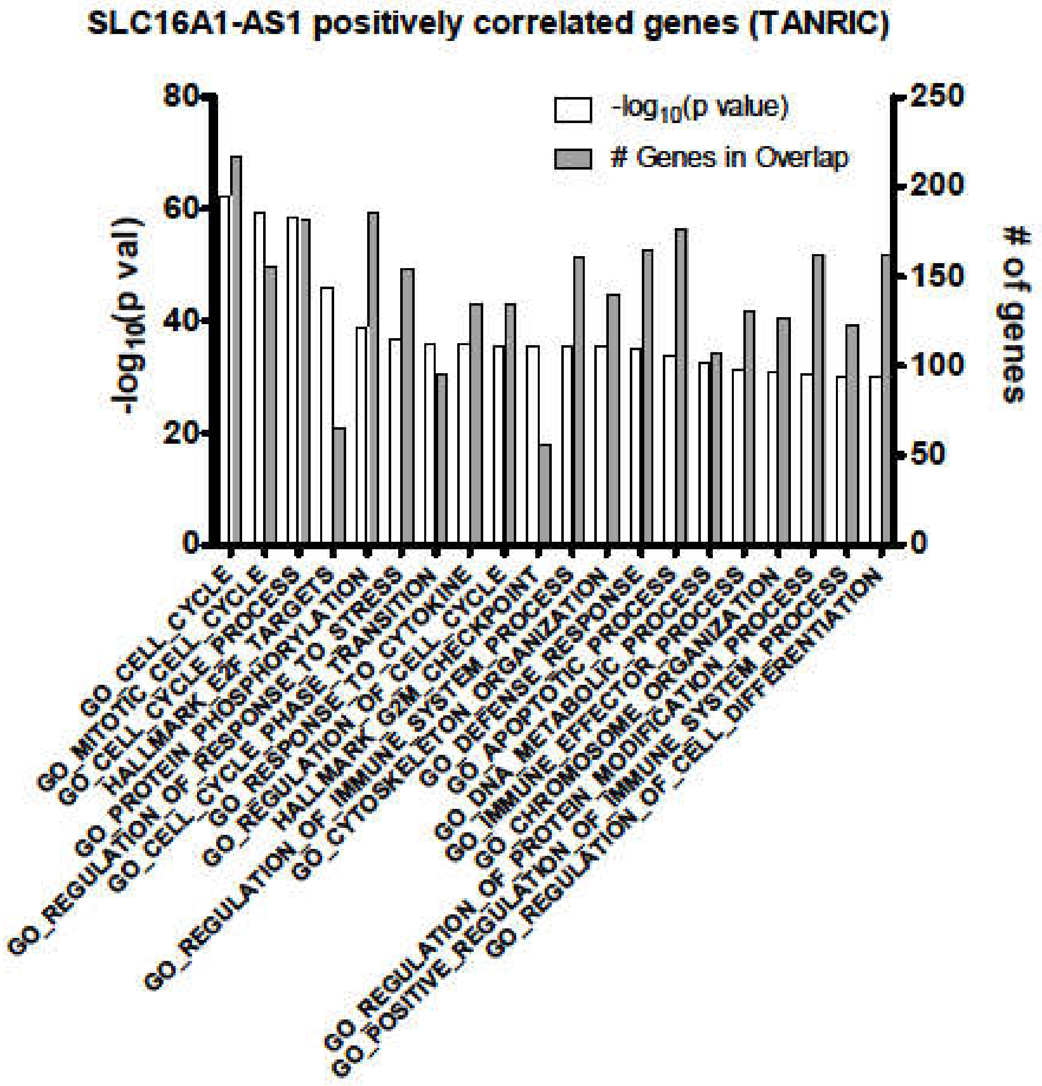

B

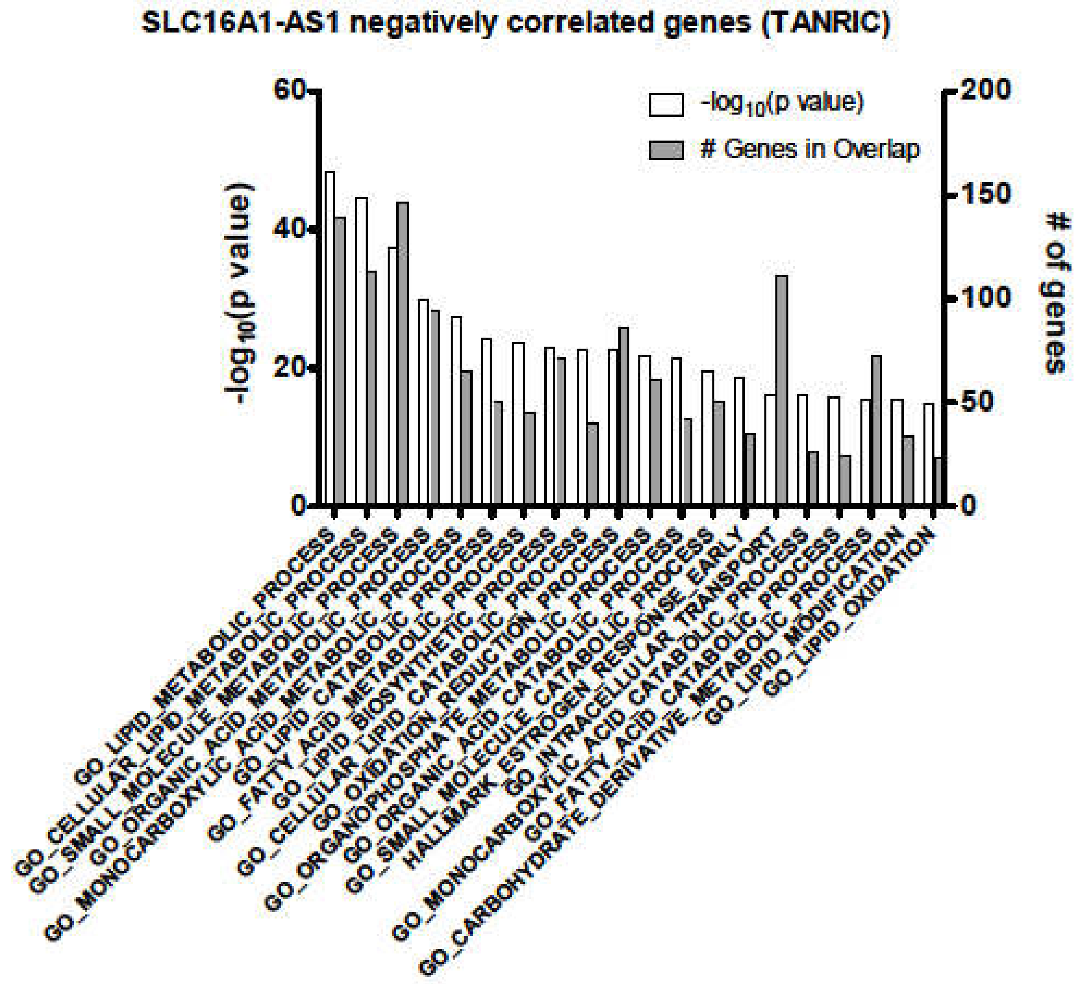

Figure S8. GSEA of genes from TANRIC atlas (A) positively (n = 1245) and (B) negatively (n = 991) correlated (|k| ≥ 0.4) with SLC16A1-AS1.

Figure S9 (related to Figure 5E, F)

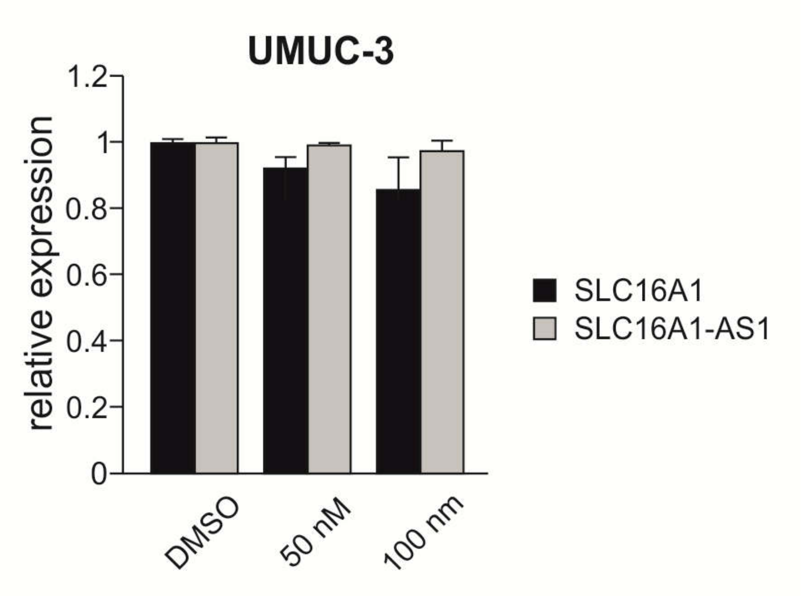

Figure S9. Quantification of SLC16A1 and SLC16A1-AS1 in UMUC-3 cells. Cells were treated with MCT1 inhibitor (AR-C155858) at indicated concentration and mRNA/lncRNA level were measured by qPCR 24 h later. The expression was normalized (GAPDH) and compared to the DMSO control (set as1). Bar graphs are represented as means ± SD (n=3).
